# Lipophilicity trends upon fluorination of isopropyl, cyclopropyl and 3-oxetanyl groups

**DOI:** 10.3762/bjoc.16.182

**Published:** 2020-09-02

**Authors:** Benjamin Jeffries, Zhong Wang, Robert I Troup, Anaïs Goupille, Jean-Yves Le Questel, Charlene Fallan, James S Scott, Elisabetta Chiarparin, Jérôme Graton, Bruno Linclau

**Affiliations:** 1 School of Chemistry, University of Southampton, Highfield, Southampton SO17 1BJ, UK; 2 Université de Nantes, CNRS, CEISAM UMR 6230, F-44000 Nantes, France; 3 Medicinal Chemistry, Oncology R&D, AstraZeneca, Cambridge CB4 0WG, UK

**Keywords:** aliphatic fluorination, cyclopropane, isopropyl, isostere, lipophilicity, oxetane

## Abstract

A systematic comparison of lipophilicity modulations upon fluorination of isopropyl, cyclopropyl and 3-oxetanyl substituents, at a single carbon atom, is provided using directly comparable, and easily accessible model compounds. In addition, comparison with relevant linear chain derivatives is provided, as well as lipophilicity changes occurring upon chain extension of acyclic precursors to give cyclopropyl containing compounds. For the compounds investigated, fluorination of the isopropyl substituent led to larger lipophilicity modulation compared to fluorination of the cyclopropyl substituent.

## Introduction

The introduction of small alkyl groups onto bioactive compounds as space filling groups is a common strategy in the drug optimization process. It is typically employed to ensure proper fitting of the part(s) of a ligand that interact with a receptor [1–2]. Apart from volume considerations, shape complementarity is regarded as important as well [3–5]. Hence, the modification of existing appendages is also commonly employed. For example, an isopropyl and a trifluoromethyl group have very similar volumes, but a very different shape [6].

However, even the introduction of relatively small methyl groups can impart profound consequences (“the magic methyl effect”) [7–9] on the activity and conformational profile, which can be very beneficial if this promotes the population of the desired bioactive conformation(s). The introduction or extension of alkyl groups generally leads to an increase in lipophilicity, which is more often than not undesired. Hence, this has led to an interest in how to add volume without adding lipophilicity, i.e., how to extend a carbon chain without a concomitant increase in log*P*.

**Figure 1:**
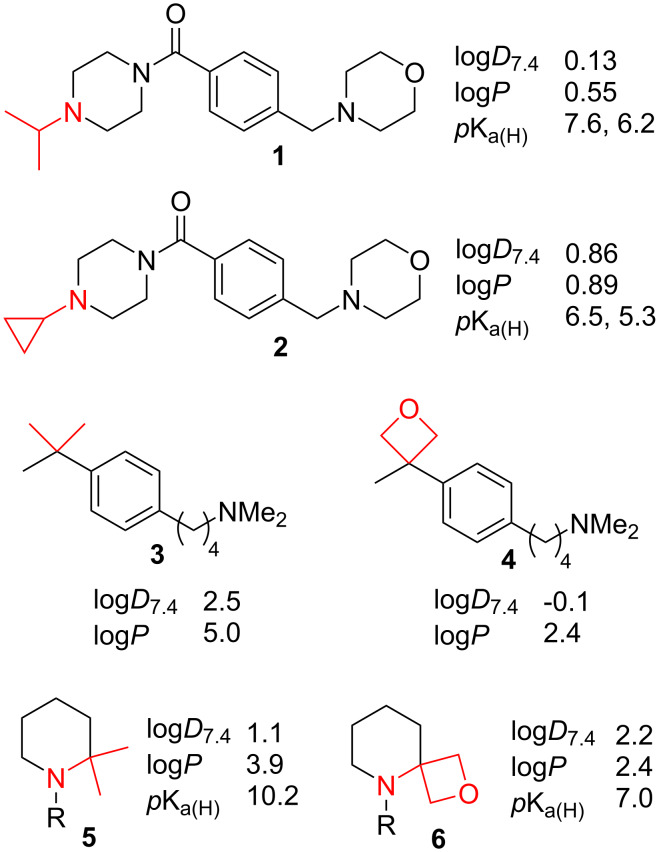
Examples of lipophilicity modulation for geminal dimethyl to cyclopropyl and oxetane modifications (measured experimentally via shake-flask method).

A much-used isosteric replacement is the change of an isopropyl group, or more commonly an α,α-disubstituted geminal dimethyl group [10], into a cyclopropyl group [11–12]. Apart from a slight shape/volume alteration caused by the significant change in bond angles and the deletion of two hydrogen atoms, this bioisosteric modification also usually results in improved metabolic stability, increased rigidity and, due to its electron-withdrawing nature, p*K_a_* modulation of adjacent acid/base groups. Importantly, it also leads to a lipophilicity reduction, as illustrated by the Hansch π-values of isopropyl (1.53) [13] and cyclopropyl (1.14) [14]. This compares to π = 1.98 for a *tert*-butyl group [13]. However, exceptions are known, as illustrated for **1** and **2** (Figure 1) [15]. In this case, the expected lipophilicity reducing effect is offset by the electron-withdrawing effect on the nitrogen (Δlog*P* +0.34). The reduction in amine basicity also shifts the distribution of free base and protonated species, resulting in a higher proportion of free base in solution at pH 7.4, which leads to a large log*D*_7.4_ increase (Δlog*D*_7.4_ +0.73).

The modification of a geminal dimethyl moiety to an oxetanyl group has also proven to be a useful biomimetic replacement leading to a significant log*P* decrease (compare **3** with **4**) [16–18]. However, when an amino group is located in the α-position (**5**), the introduction of the oxetanyl group leading to **6** induces a significant p*K_a(H)_* decrease. While the reduction in log*P* is still observed, the larger proportion of unprotonated substrate leads to a log*D*_7.4_ increase.

**Figure 2:**
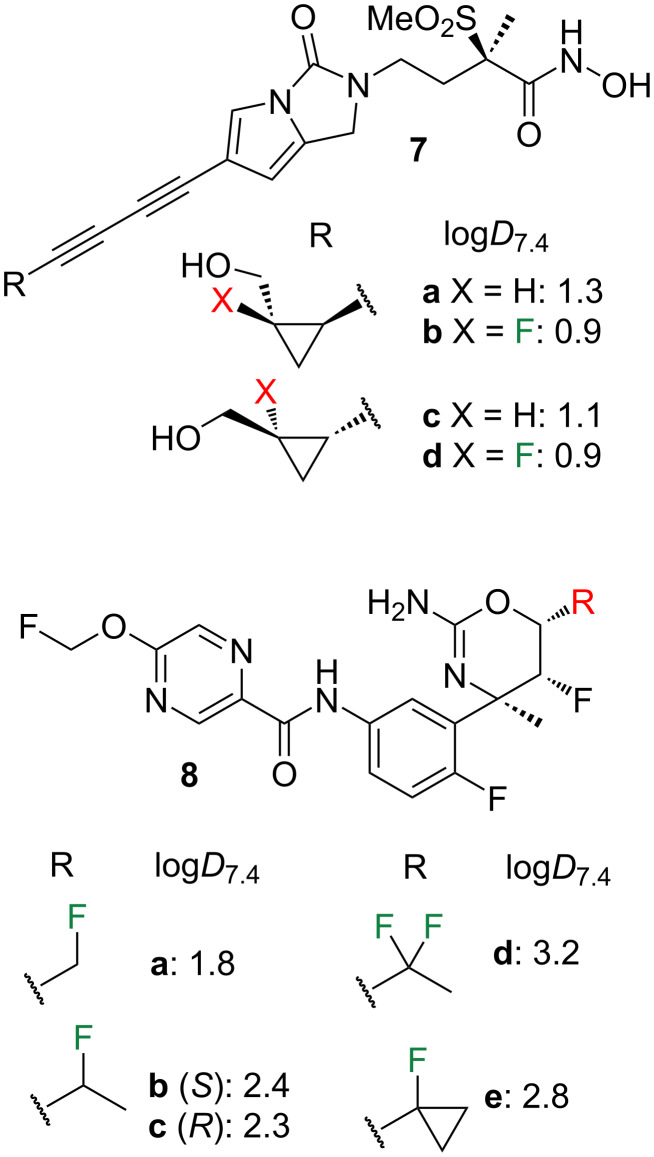
Lipophilicity modulation examples involving fluorinated cyclopropane derivatives (measured experimentally via shake-flask method).

Aliphatic fluorination can also be employed to decrease lipophilicities [6,19–22]. However, there is comparatively less precedence of lipophilicity comparisons between fluorinated isopropyl groups, cyclopropanes and oxetanes. Examples of bioactive compounds are given in Figure 2.

During the optimization of the LpxC inhibitor **7a** [23], it was shown that fluorination to give analogue **7b** reduced the lipophilicity. Interestingly, while the nonfluorinated **7a** and **7c** diastereomers have different log*D*_7.4_ values, this is not the case for their fluorinated analogues **7b** and **7d**. Oxazine derivative **8** featured in the development of centrally active β-secretase (BACE1) inhibitors [24]. Although the log*D*_7.4_ of the unsubstituted cyclopropyl containing analogue was not provided, it can be seen that two-carbon extension of **8a**, and one-carbon extension of **8b**,**c** to give the cyclopropyl derivative **8e**, cause a lipophilicity increase. Conversely, changing a CF_2_Me for a cPrF group (**8d** to **8e**) gives a lipophilicity reduction.

**Figure 3:**
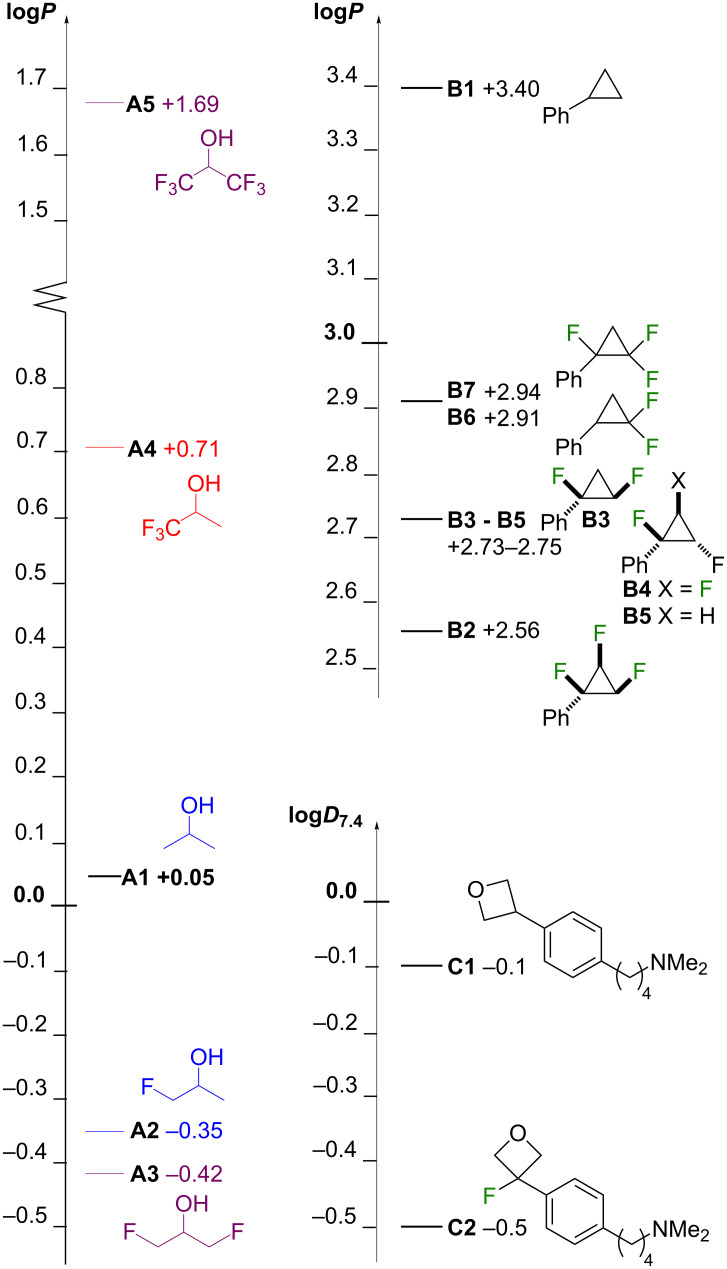
Lipophilicity changes upon fluorination of isopropyl, cyclopropane and oxetane rings (Series **A**, **C**: measured experimentally via shake-flask method; series **B**: measured experimentally by reversed-phase HPLC).

Finally, a number of model compound series have been reported (Figure 3). In the isopropanol series **A** [22], monofluorination led to a 0.4 log*P* decrease, which is further extended upon a second monofluorination at the other methyl group (compare **A1** with **A2**, **A3**). With one (**A4**) and two (**A5**) trifluoromethyl groups, large increases in lipophilicity are observed. An interesting series (**B**) of phenylcyclopropanes was reported by O’Hagan [25–26]. All-*cis* vicinal trifluorination as in **B2** led to a significant reduction in lipophilicity compared to the nonfluorinated **B1**, which was explained by the facially polar motif caused by the C–F dipoles present on the same side of the ring. Consequently, the diastereomer **B4** possessed a higher lipophilicity, although the lipophilicity of the vicinally difluorinated diastereomers **B3** and **B5** was identical, and also the same as the trifluorinated **B4**. Geminal difluorination caused an increase in lipophilicity compared to vicinal fluorination (compare **B6** with **B3** and **B5**, and **B7** with **B4** and **B2**). Interestingly, in analogy with the same lipophilicity of **B3**–**B4**, **B6** and **B7** also were equivalent in lipophilicity. Finally, the 3-fluorinated oxetanyl derivative **C2** has a lower log*D* value than the nonfluorinated parent **C1** [16–17].

**Figure 4:**
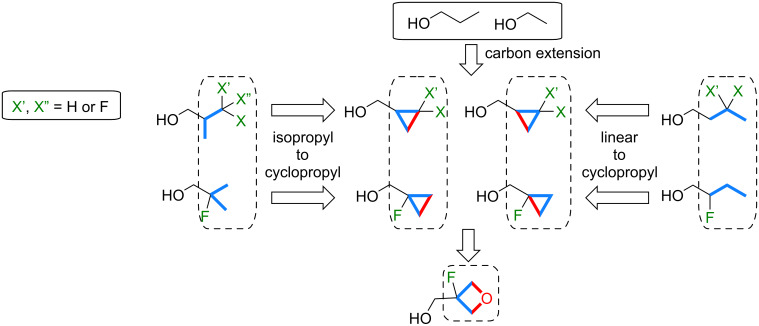
Lipophilicity modulations discussed in this contribution.

In this contribution, we describe a systematic study on the lipophilicity modulations of the isopropyl, cyclopropyl and 3-oxetanyl groups and their various possible analogues, featuring fluorination at a single carbon atom (Figure 4), using the directly comparable isobutanol, cyclopropylmethanol and 3-oxetanylmethanol scaffolds. All compounds are either commercially available or are easily synthesized from available fluorinated building blocks. In addition, comparison of lipophilicities of relevant linear butanol derivatives is discussed, as well as that of the introduction of an isopropyl/cyclopropyl/oxetanyl group from 1-propanol and ethanol, which represents carbon extensions (volume increase).

## Results and Discussion

### Lipophilicity data and discussion

**Figure 5:**
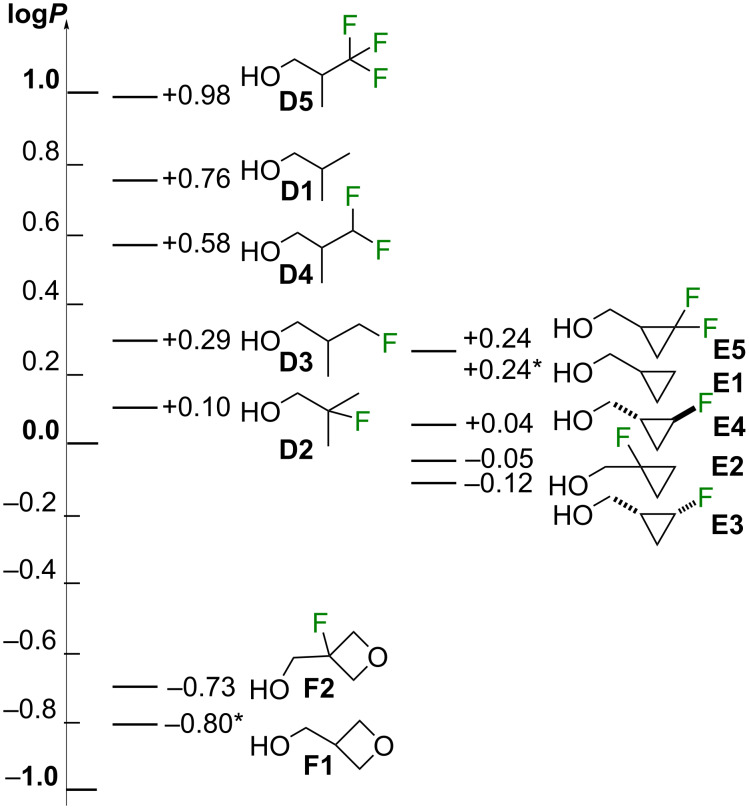
Distribution of the experimental lipophilicity values of series **D**, **E** and **F** (* denotes an estimated value, see Supporting Information File 1).

The lipophilicities of the nonfluorinated **E1** and **F1** have not been reported, and have been estimated by a range of calculation methods as described in Supporting Information File 1 (section 2). The average log*P* value of the data from those estimation approaches for **E1** was determined to be 0.24 ± 0.03, and for **F1** −0.80 ± 0.23. The experimental lipophilicity values of the fluorinated isobutanol, cyclopropylmethanol and 3-oxetanylmethanol derivatives are given in Figure 5. They were measured by a ^19^F NMR-based method developed by our group [22,27], which is suitable for measuring the octanol/water partition coefficients *P* of (fluorinated) non-UV active substrates.

As expected, monofluorination decreases the lipophilicity compared to the nonfluorinated parent **D1**, with a much larger decrease for the β-fluorohydrin **D2** than for the γ-fluorohydrin **D3**. Interestingly, for the linear propanol, β-fluorination and γ-fluorination lead to a similar log*P* decrease (−0.29 vs −0.26 for 2- and 3-fluoropropanol) [22], while for the higher alkanols, β-fluorination leads to more lipophilic compounds than γ-fluorination [28]. The higher log*P* of β-fluorinated alcohols has been explained both by the occurrence of conformations with opposing dipole moments of the C–F and C–O bonds, as well as the reduction of polarizability of the oxygen lone pairs due to the fluorine electronegativity. However, while **D2** is a β-fluorohydrin, its fluorine is substituted at a tertiary position, with the C–F bond able to polarize the six C–H bonds of the methyl groups, which has a lipophilicity lowering effect. The electron-withdrawing effect of the fluorine is evident from the ^1^H NMR chemical shift values of the CH_3_ groups in **D1** (0.91 ppm) [29], **D3** (0.98 ppm), and **D2** (1.37 ppm) [30].

Compared to the monofluorinated **D3**, difluorination (**D4**) and trifluorination (**D5**) at the same carbon atom increases lipophilicity. There is a notable difference in lipohilicity between **D4** and **D5**. Aliphatic compounds containing a CF_2_H group are frequently less lipophilic than to their nonfluorinated equivalent [20,31] (except in presence of a vicinal C–O bond [22,32]), which is what is found here for **D4**. The CF_3_-containing **D5** is more lipophilic than the parent **D1**, and these values are consistent with the trends seen for 1-propanol [22].

For cyclopropylmethanol **E1**, β-fluorination (**E2**) leads to a smaller lipophilicity decrease compared to that seen for **D1**→**D2**, leading to a log*P* value in between those of the two γ-fluorinated diastereomers **E3** and **E4**. The decrease seen for **E2** is in accord with the observations for the β-fluorinated analogues of **7a** and **7c** (see above, Figure 2). The *cis*-isomer **E3** has a slightly lower lipophilicity than the *trans*-isomer **E4**. The lower lipophilicity decrease upon monofluorination in series **E** compared to series **F** could be related to the hybridization state of cyclopropyl carbon atoms. However, β-fluorination of the oxetanyl derivative **F1** led to a lipophilicity increase. With the caveat that the value for **F1** is not a measured value and the difference is well within the standard deviation (0.23 log*P* units), this increase is in contrast to the result observed for **C1** (see above, Figure 3).

**Figure 6:**
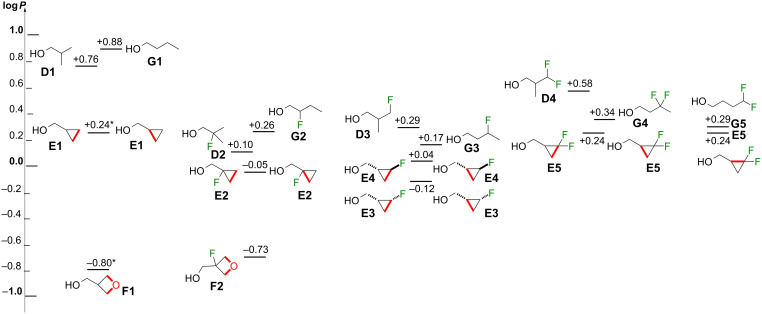
Comparison of lipophilicities between the linear alkyl, isopropyl, cyclopropyl, and 3-oxetanyl substituents by fluorination level (exchange two C–H bonds by a C**–**C bond or C**–**O**–**C bonds, same carbon count).

As discussed in the Introduction, converting acyclic alkanes to cyclopropane equivalents is a frequently used tactic in medicinal chemistry. The pairwise comparison of the isopropyl with the cyclopropyl substructures is best discussed via Figure 6, with the bold red bond emphasizing the structural change (cf **D1** vs **E1**). For completion, the change observed from the corresponding linear 1-butanol equivalents with equidistant alcohol and fluorine groups, by connecting C2 and C4 as shown (cf **G1** to **E1**), is also considered (see Supporting Information File 1, Figure S1 for a summary figure showing changes in log*P* between linear alkanols and cyclopropylmethanols).

Firstly, the isobutanol (**D**) and their corresponding 1-butanol (**G**) series are compared. It is known that branching causes a reduction in log*P*, which has been explained by a less energetically unfavorable contact with water due to the resulting reduction in hydrophobic surface [33] (compare **D1** with **G1**, Δ 0.12). This difference is similar upon β-fluorination (**D2** vs **G2**, Δ 0.16) with a slightly greater magnitude which, as discussed above, is potentially due to the polarization of C–H bonds by fluorine. Interestingly, for γ-mono- and γ,γ-difluorination the linear butanol derivatives **G3** and **G4** are now less lipophilic than the branched **D3** and **D4**, respectively. A possible explanation is that branching in **D3** and **D4** results in shielding of the polar C–F bonds preventing dipolar interactions.

Regarding the conversion of the iso- and *n*-butyl chains to the cyclopropylmethyl arrangement, as expected a significant lipophilicity decrease is observed for the nonfluorinated derivatives (compare **D1**, **G1** with **E1**), with another large decrease when converting the cyclopropyl group to an oxetanyl moiety (compare **E1** with **F1**). A much lower lipophilicity decrease is seen when converting the acyclic β-fluorinated compounds **D2** and **G2** to the cyclopropyl analogue **E2**, but an appreciable log*P* decrease is still seen going from **E2** to the oxetanyl derivative **F2**. With γ-fluorination, the log*P* decreases are similarly small, both for monofluorination (compare **D3**, **G3** with **E3**, **E4**) and for geminal difluorination (compare **D4**, **G4** with **E5**). Finally, converting the linear 4,4-difluorobutan-1-ol (**G5**) to the corresponding difluorinated cyclopropylmethanol **E5** only results in a minor lipophilicity decrease. Nevertheless, in all cases, for the same fluorination motif, the cyclopropane derivatives have a lower lipophilicity compared to their acyclic equivalents.

**Figure 7:**
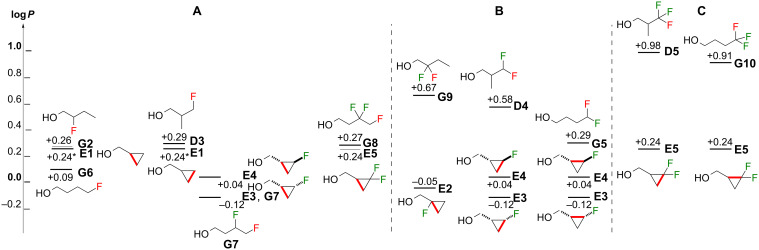
Comparison of lipophilicities between isopropyl and cyclopropyl substituents grouped by exchange of C–H/C–F bonds by a C**–**C bond (same carbon count). Organised by the exchanged fluorine being the only fluorine substituent on the carbon involved (**A**) or as part of a CF_2_ (**B**) or CF_3_ (**C**) group.

It is also useful to compare lipophilicities of acyclic and cyclopropane derivatives in which the isosterism represents conversion of a C–H and C–F bond into a C–C bond (Figure 7), hence transforming an acyclic to a cyclic group. Some interesting trends are identified. When the chain ring is closed with the loss of a C–F that was part of a single fluorine motif (Figure 7A), only very minimal lipophilicity differences were then observed. For 4-fluorobutan-1-ol (**G6**), and 3,4-difluorobutan-1-ol (**G7**), which have the lowest lipophilicities of the linear butanol series measured so far [28], the corresponding cyclopropyl isosteres **E1** and **E4** have a slightly higher lipophilicity, while **E3** has the same lipophilicity as **G7**. The lipophilicity-reducing power of the vicinal 1,2-difluoromotif in **G7** is well-described [34–35], but at least for series **D**, the motif present in **E3** is another efficient candidate when a log*P* reduction operation is in order. In contrast, other such ring closures (e.g., starting from **G2**, **D3**, and **G8**) lead to a minute log*P* decrease.

The situation is different when the “cyclisation” operation is achieved from geminal motifs (Figure 7B). Significant log*P* reductions are observed, especially from the internal geminal fluorination motif as in **G9** to **E2** (0.72 log*P* units). Starting from a trifluoromethyl group (Figure 7C), similar log*P* reductions are achieved (e.g., **D5**→**E5** and **G10**→**E5**) which interestingly, are of the same magnitude as for the nonfluorinated derivatives (compare **D1**, **G1** to **E1**, Figure 6). It is worth noting that “cyclisation” to give cyclopropanol derivatives substituted with a fluorinated methyl group (not shown) can also be considered. Marketed drugs with such substructures include Voxilaprevir and Glecaprevir.

Finally, it is useful to consider the lipophilicity changes upon extending a propyl or ethyl chain to a cyclopropylmethyl moiety, which represents a one-carbon and a two-carbon extension, respectively. It is known that adding a spiro-cyclopropyl moiety onto a methylene group of an aliphatic chain, which represents a two-carbon extension, can lead to a lipophilicity reduction [16–17].

**Figure 8:**
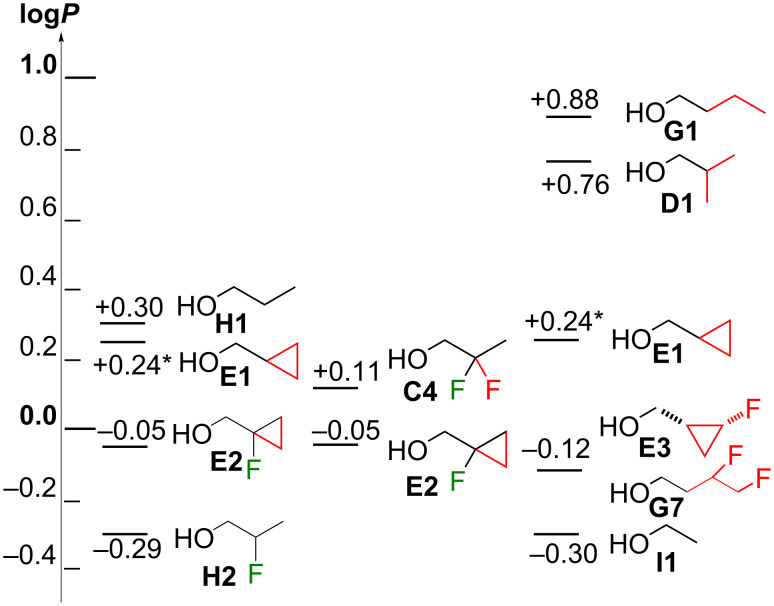
Carbon extensions.

The one-carbon extension from **H1** to give **E1** results in a small lipophilicity decrease (Figure 8), which is slightly extended with further β-fluorination (**E2**). In contrast, the one-carbon introduction of a cyclopropyl group in **H2** to give **E2** leads to a lipophilicity increase. Replacing the fluorine substituent by a cyclopropyl carbon (compare **H2** with **E1**) also leads to a lipophilicity increase, but not when starting from a geminal difluoro motif (compare **C4** with **E2**).

The two-carbon extension from **I1** to the acyclic derivatives **D1** and **G1** leads to a large lipophilicity increase, which is much smaller for a two-carbon cyclopropyl extension (**E1**). This lipophilicity increase is somewhat attenuated with concomitant β-fluorination (**E2**) and γ-fluorination (**E3**). Interestingly, a two-carbon extension of **I1** with concomitant vicinal difluorination (**G7** [28]) or fluorinated cyclopropyl (**E3**) leads to a much smaller increase in log*P* compared to two-carbon extension as a cyclopropyl (**E1**).

### Computational results

A number of open-access fragment-based calculation methods, as well as the internal AstraZeneca method, were used to obtain clog*P* values of the fluorinated butanol, isobutanol, cyclopropylmethanol and 3-oxetanylmethanol derivatives mentioned above (Supporting Information File 1, Table S6). For each method, the correlations with the experimental data were obtained (Supporting Information File 1, Figures S7–S11). Three methods gave excellent correlations (with *r*^2^ between 0.86 and 0.91, Supporting Information File 1, Table S7), but other methods gave only low correlations (*r*^2^ = 0.36, 0.49). This is perhaps due to the training sets used, but it clearly demonstrates that even for a set of relatively simple compounds, fragment-based clog*P* calculations are not guaranteed to give reliable lipophilicity data.

Next, theoretical lipophilicities were obtained using DFT calculations, based on the notion that the partition coefficient of a given solute between two phases relates to the difference in Gibbs energy of the free ligand conformations of this solute in the respective solvents. Quantum chemistry calculations of Gibbs energies in octanol and water thus provide theoretical estimations of lipophilicities. Such estimations require systematic conformational analyses and geometry optimizations. Ho and co-worker showed that the use of the lowest-energy conformations as calculated in the respective solvents gave more accurate lipophilicities than the use of the lowest energy conformations in the gas phase [36]. Somewhat surprisingly, implicit solvent models proved superior than explicit models, despite their shorter calculation times, with the SMD implicit solvent model being superior compared to the IEF-PCM and C-PCM models [36–37]. However, the implicit octanol solvent model does not take into account the large amount of water present in saturated octanol [36].

We have previously used the SMD implicit solvation model with the MN15 functional in theoretical lipophilicity predictions of linear fluorohydrins [27–28], which gave an excellent correlation with the experimental values (*r*^2^ = 0.957), although the absolute values were much higher (a slope of >2 was obtained).

**Figure 9:**
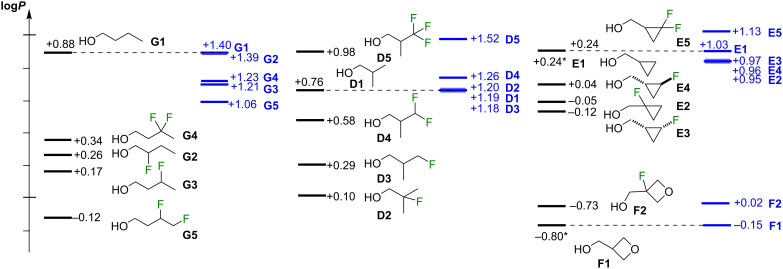
Experimental and theoretical (in blue) values (calculated at the MN15/aug-cc-pVTZ//MN15/cc-pVTZ level of theory). Values for **G1**, **G4**, **G7**, **G8** taken from the literature [28]. The distance between the lines relate to the relative lipophilicity differences. Experimental and theoretical data sets normalized towards the parent compound.

Unfortunately, the theoretical lipophilicity values computed in the present work using the same methodology for series **D**, **E**, and **F**, as well as for the linear butanols **G1**–**G4**, **G7**, **G8**, pictured in Supporting Information File 1, Figure 9 and given in Supporting Information File 1, Table S11, did not provide any useful approximation in terms of rank order or absolute magnitude of effect.

For series **D**, **E**, **G**, the DFT-log*P* values cluster together with minimal lipophilicity differences, and within mean absolute error limits (estimated to be around 0.8 log*P* units), making detailed considerations meaningless. This was a surprise, as series **D** and **E** are relatively rigid, which simplifies conformational analysis. The remarkably similar DFT-log*P* values suggest that the influence of the fluorination is underrepresented in the calculations. The following observations are noteworthy. The trifluorinated **D5** was calculated to have the largest log*P* value, in accord with the experiment. The geminally difluorinated derivatives are calculated to be more lipophilic than the monofluorinated derivatives, except in series **G**: **G4** was calculated to be less lipophilic than **G2**. The **E3**/**E4** diastereomers were calculated to have essentially the same lipophilicities.

**Figure 10:**
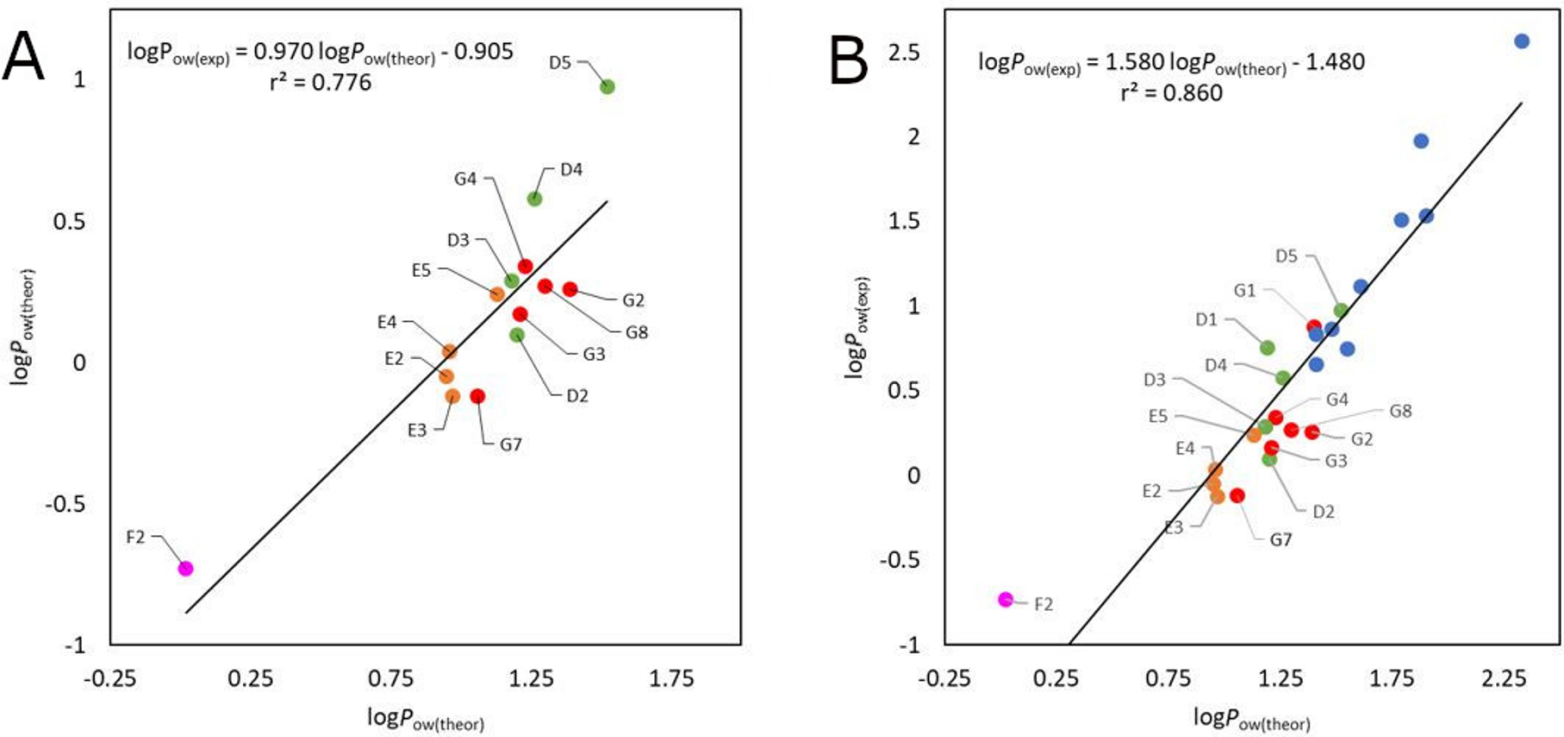
Correlation of the DFT-calculated lipophilicities with the experimental values. A) fluorinated series **D**, **E**, **F**, **G2**–**4**, **G7**, **G8**, B) Compilation with other fluorohydrins (taken from reference [28]).

The correlation with the experimental values shows a coefficient of determination value of 0.776 (Figure 10A), which is worse than many of the investigated fragment-based clog*P* methods (see above), for which such clustering is not observed. When these values are combined with the full available set of fluorohydrins calculated with the MN15 functional (Figure 10B), the correlation is now reduced from *r*^2^ = 0.957 to 0.860.

**Figure 11:**
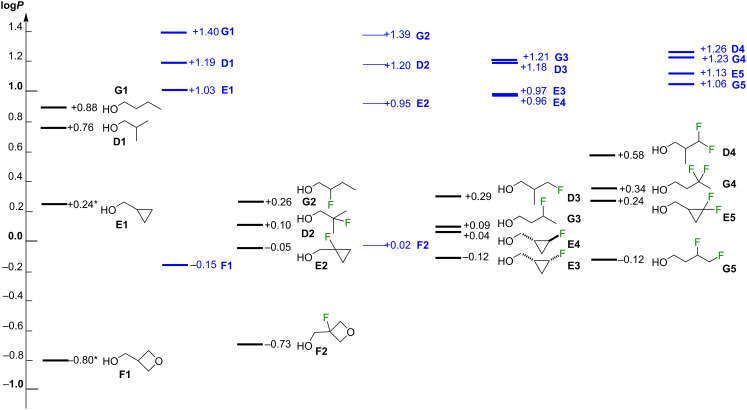
Experimental (in black) and theoretical (in blue) values (calculated at the MN15/aug-cc-pVTZ//MN15/cc-pVTZ level of theory) of all compounds, organized by motif.

Interestingly, the theoretical and experimental values, while much different in absolute value and in relative difference, correspond almost perfectly regarding lipophilicity order when arranged by motif (Figure 11). As a point of interest, the calculations correctly estimate a lower lipophilicity for the branched **D1** and **D2** vs **G1** and **G2**, but cannot significantly distinguish between the linear and branched γ-mono- and difluorinated **G3**/**D3** and **G4**/**D4**. Cyclopropyl structures are always less lipophilic than their corresponding acyclic counterparts.

## Conclusion

Monofluorination of isobutanol significantly decreased the lipophilicity, with β-fluorination leading to a lower log*P* value than γ-fluorination. As expected, geminal difluorination also decreased the log*P*, but to a lesser extent compared to monofluorination. For the corresponding cyclopropyl derivative, cyclopropylmethanol, monofluorination at the β or γ position only led to a minor decrease in log*P*, and no log*P* difference is observed with the geminal difluorinated analogue. The typically observed lower lipophilicities of branched isomers vs their linear isomers are also observed for β-fluorohydrin containing compounds, but not when γ-mono- or difluorinated. Equally, converting (acyclic) alkyl groups to the corresponding cyclopropane equivalents (C–H/C–H→C–C) typically leads to a lipophilicity decrease, which remains the case when fluorination is present. For a C–F/C–H→C–C ‘cyclisation’, only minimal lipophilicity differences are observed (in either direction) when the C–F moiety was part on a monofluorinated motif. However, when the C–F moiety was part of a geminal (CF_2_ or CF_3_) motif, a large log*P* reduction is observed.

Fragment based clog*P* methods show great variation in their ability to estimate the lipophilicities of the small alkanol compounds described herein. To our surprise, theoretical lipophilicity predictions using the MN15 functional with the SMD implicit solvation model also performed poorly, despite the fact that most of the substrates are small and relatively rigid compounds. It was found that for a given series, the DFT-log*P* values are clustered together, indicating the influence of the fluorination is underrepresented in the calculations. This clearly indicates that further research towards theoretical lipophilicity prediction based in relative Gibbs energies in water and (wet) octanol is required.

## Supporting Information

File 1 Synthesis, characterisation and copies of spectra of the novel compounds, details of the calculations, log*P* determinations of the nonfluorinated parents **E1** and **F1**, and experimental measurements of the lipophilicities of the fluorinated derivatives.

## References

[1] Persch E, Dumele O, Diederich F (2015). Angew Chem, Int Ed.

[2] Mecozzi S, Rebek J (1998). Chem – Eur J.

[3] Bissantz C, Kuhn B, Stahl M (2010). J Med Chem.

[4] Zauhar R J, Moyna G, Tian L, Li Z, Welsh W J (2003). J Med Chem.

[5] Kumar A, Zhang K Y J (2018). Front Chem (Lausanne, Switz).

[6] Smart B E (2001). J Fluorine Chem.

[7] Schönherr H, Cernak T (2013). Angew Chem, Int Ed.

[8] Kuntz K W, Campbell J E, Keilhack H, Pollock R M, Knutson S K, Porter-Scott M, Richon V M, Sneeringer C J, Wigle T J, Allain C J (2016). J Med Chem.

[9] Barreiro E J, Kümmerle A E, Fraga C A M (2011). Chem Rev.

[10] Talele T T (2018). J Med Chem.

[11] Talele T T (2016). J Med Chem.

[12] Novakov I A, Babushkin A S, Yablokov A S, Nawrozkĳ M B, Vostrikova O V, Shejkin D S, Mkrtchyan A S, Balakin K V (2018). Russ Chem Bull.

[13] Hansch C, Leo A, Unger S H, Kim K H, Nikaitani D, Lien E J (1973). J Med Chem.

[14] Skagerberg B, Bonelli D, Clementi S, Cruciani G, Ebert C (1989). Quant Struct-Act Relat.

[15] Letavic M A, Aluisio L, Apodaca R, Bajpai M, Barbier A J, Bonneville A, Bonaventure P, Carruthers N I, Dugovic C, Fraser I C (2015). ACS Med Chem Lett.

[16] Wuitschik G, Carreira E M, Wagner B, Fischer H, Parrilla I, Schuler F, Rogers-Evans M, Müller K (2010). J Med Chem.

[17] Wuitschik G, Rogers-Evans M, Müller K, Fischer H, Wagner B, Schuler F, Polonchuk L, Carreira E M (2006). Angew Chem, Int Ed.

[18] Bull J A, Croft R A, Davis O A, Doran R, Morgan K F (2016). Chem Rev.

[19] Böhm H-J, Banner D, Bendels S, Kansy M, Kuhn B, Müller K, Obst-Sander U, Stahl M (2004). ChemBioChem.

[20] Huchet Q A, Kuhn B, Wagner B, Fischer H, Kansy M, Zimmerli D, Carreira E M, Müller K (2013). J Fluorine Chem.

[21] Müller K, Haufe G, Leroux F (2018). Fluorination patterns in small alkyl groups: their impact on properties relevant to drug discovery. Fluorine in Life Sciences: Pharmaceuticals, Medicinal Diagnostics, and Agrochemicals.

[22] Linclau B, Wang Z, Compain G, Paumelle V, Fontenelle C Q, Wells N, Weymouth-Wilson A (2016). Angew Chem, Int Ed.

[23] Panchaud P, Surivet J-P, Diethelm S, Blumstein A-C, Gauvin J-C, Jacob L, Masse F, Mathieu G, Mirre A, Schmitt C (2020). J Med Chem.

[24] Nakahara K, Fuchino K, Komano K, Asada N, Tadano G, Hasegawa T, Yamamoto T, Sako Y, Ogawa M, Unemura C (2018). J Med Chem.

[25] Fang Z, Cordes D B, Slawin A M Z, O'Hagan D (2019). Chem Commun.

[26] Thomson C J, Zhang Q, Al-Maharik N, Bühl M, Cordes D B, Slawin A M Z, O’Hagan D (2018). Chem Commun.

[27] Wang Z, Jeffries B F, Felstead H R, Wells N J, Chiarparin E, Linclau B (2019). J Visualized Exp.

[28] Jeffries B, Wang Z, Felstead H R, Le Questel J-Y, Scott J S, Chiarparin E, Graton J, Linclau B (2020). J Med Chem.

[29] Siebum A H G, Woo W S, Lugtenburg J (2003). Eur J Org Chem.

[30] Scott J S, Bailey A, Buttar D, Carbajo R J, Curwen J, Davey P R J, Davies R D M, Degorce S L, Donald C, Gangl E (2019). J Med Chem.

[31] Zafrani Y, Sod-Moriah G, Yeffet D, Berliner A, Amir D, Marciano D, Elias S, Katalan S, Ashkenazi N, Madmon M (2019). J Med Chem.

[32] Kubyshkin V, Budisa N (2017). Beilstein J Org Chem.

[33] Bazzini P, Wermuth C G, Wermuth C G (2008). Substituents and Functions: Qualitative and Quantitative Aspects of Structure–Activity Relationships. The Practice of Medicinal Chemistry.

[34] Huchet Q A, Kuhn B, Wagner B, Kratochwil N A, Fischer H, Kansy M, Zimmerli D, Carreira E M, Müller K (2015). J Med Chem.

[35] Erdeljac N, Kehr G, Ahlqvist M, Knerr L, Gilmour R (2018). Chem Commun.

[36] Kundi V, Ho J (2019). J Phys Chem B.

[37] Michalík M, Lukeš V (2016). Acta Chim Slovaca.

