# Supplementary material for: Lipophilicity trends upon fluorination of isopropyl, cyclopropyl and 3-oxetanyl groups
Source: Beilstein J Org Chem. 2020 Sep 2;16:2141–50. doi: 10.3762/bjoc.16.182 (PMC7476584; doi:10.3762/bjoc.16.182)
Supplement: File 1 — Synthesis, characterisation and copies of spectra of the novel compounds, details of the calculations, logP determinations of the nonfluorinated parents E1 and F1, and experimental measurements of the lipophilicities of the fluorinated derivatives. [file Beilstein_J_Org_Chem-16-2141-s001.pdf]

BEILSTEIN JOURNAL OF ORGANIC CHEMISTRY

Supporting Information

for

Lipophilicity trends upon fluorination of isopropyl, cyclopropyl and 3-oxetanyl groups

Benjamin Jeffries, Zhong Wang, Robert I. Troup, Anaïs Goupille, Jean-Yves Le Questel, Charlene Fallan, James S. Scott, Elisabetta Chiarparin, Jérôme Graton and Bruno Linclau

Beilstein J. Org. Chem. **2020,** *16,* 2141–2150. doi:10.3762/bjoc.16.182

Synthesis, characterisation and copies of spectra of the novel compounds, details of the calculations, log*P* determinations of the nonfluorinated parents E1 and F1, and experimental measurements of the lipophilicities of the fluorinated derivatives

License and Terms: This is a supporting information file under the terms of the Creative Commons Attribution License (https://creativecommons.org/licenses/by/4.0). Please note that the reuse, redistribution and reproduction in particular requires that the authors and source are credited. The license is subject to the *Beilstein Journal of Organic Chemistry* terms and conditions: (https://www.beilstein-journals.org/bjoc)

Contents

1 Comparison between linear, isopropyl and cyclopropyl (Figure S1) S5

2 Determination of the clog*P* of the parent compounds cyclopropylmethanol E1 and (3-oxetanyl)methanol F1 ....S6

2.1 Experimental log*P* values[a] for selected nonfluorinated alkanols (Table S1) S6

2.2 Clog*P* values for selected nonfluorinated alkanols (Table S2) S7

2.3 Correlation of clog*P* with experimental data for nonfluorinated alkanols (Figures S2–S6) S9

2.3.1 Molinspiration (Figure S2) S9

2.3.2 Biobyte (Figure S3) S9

2.3.3 Marvinsketch (Figure S4) S10

2.3.4 ACDlog*P* (Figure S5) S10

2.3.5 AZlog$D_{7.4}$ (Figure S6) S11

2.3.6 Trendlines (Table S3) S11

2.4 Estimated log*P* values: Cyclopropylmethanol (Table S4) S12

2.5 Estimated log*P* values: (3-oxetanyl)methanol (Table S5) S12

3 clog*P* values of the fluorinated derivatives and correlation with the experimental values S13

3.1 Table with the clog*P* values of five calculation methods (Table S6) S13

3.2 Correlation of clog*P* with experimental data (Figures S7-S11) S15

3.2.1 Molinspiration (Figure S7) S15

3.2.2 Biobyte (Figure S8) S15

3.2.3 Marvinsketch (Figure S9) S16

3.2.4 ACDlogP (Figure S10) S16

3.2.5 AZlog$D_{7.4}$ (Figure S11) S17

3.2.6 Trendline (Table S7) S17

4 Experimental log*P* data S18

4.1 Determination of log*P* S18

4.2 Standard NMR settings S18

4.3 Tables S8-S10: data of all individual measurements S19

4.3.1 Isobutanol series (Table S8) S19

4.3.2 Cyclopropylmethanol series (Table S9) S20

4.3.3 Oxetan-3-ylmethanol series (Table S10) S20

4.4 Example of NMR spectra for log*P* determination, with detailed NMR parameter information and integration details after reprocessing S21

4.4.1 Octanol sample (Figure S12) S21

4.4.2 Water sample (Figure S13) S23

5 Conformational analysis in water and *n*-octanol medium. S25

5.1 General S25

5.2 All values (Table S11) S25

5.3 Conformational analysis: relative energies and populations S26

5.3.1 Table S12. Relative Gibbs energy and populations of the energetic minima of **D1** (2-methylpropan-1-ol) identified at the SMD/MN15/aug-cc-pVTZ//MN15/cc-pVTZ level of theory in water and octanol medium. S26

5.3.2 Table S13. Relative Gibbs energy and populations of the energetic minima of **D2** (2-fluoro-2-methylpropan-1-ol) identified at the SMD/MN15/aug-cc-pVTZ//MN15/cc-pVTZ level of theory in water and octanol medium. S26

5.3.3 Table S14. Relative Gibbs energy and populations of the energetic minima of **D3** (3-fluoro-2-methylpropan-1-ol) identified at the SMD/MN15/aug-cc-pVTZ//MN15/cc-pVTZ level of theory in water and octanol medium. S27

5.3.4 Table S15. Relative Gibbs energy and populations of the energetic minima of **D4** (3-fluoro-2-methylpropan-1-ol) identified at the SMD/MN15/aug-cc-pVTZ//MN15/cc-pVTZ level of theory in water and octanol medium. S28

5.3.5 Table S16. Relative Gibbs energy and populations of the energetic minima of **D5** (3,3,3-trifluoro-2-methylpropan-1-ol) identified at the SMD/MN15/aug-cc-pVTZ//MN15/cc-pVTZ level of theory in water and octanol medium. S29

5.3.6 Table S17. Relative Gibbs energy and populations of the energetic minima of **E2** (2-fluoro-2-*c*-propyl-ethanol) identified at the SMD/MN15/aug-cc-pVTZ//MN15/cc-pVTZ level of theory in water and octanol medium. S29

5.3.7 Table S18. Relative Gibbs energy and populations of the energetic minima of **E3** (2-(2’-*syn*-fluoro-*c*-propyl)-ethanol) identified at the SMD/MN15/aug-cc-pVTZ//MN15/cc-pVTZ level of theory in water and octanol medium. S30

5.3.8 Table S19. Relative Gibbs energy and populations of the energetic minima of **E4** (2-(2’-*anti*-fluoro-*c*-propyl)-ethanol) identified at the SMD/MN15/aug-cc-pVTZ//MN15/cc-pVTZ level of theory in water and octanol medium. S30

5.3.9 Table S20. Relative Gibbs energy and populations of the energetic minima of **E5** (2-(2’,2’-difluoro-*c*-propyl)-ethanol) identified at the SMD/MN15/aug-cc-pVTZ//MN15/cc-pVTZ level of theory in water and octanol medium. S31

5.3.10 Table S21. Relative Gibbs energy and populations of the energetic minima of **F2** (2-fluoro-2-oxetanyl-ethanol) identified at the SMD/MN15/aug-cc-pVTZ//MN15/cc-pVTZ level of theory in water and octanol medium. S31

5.3.11 Table S22. Relative Gibbs energy and populations of the energetic minima of **G2** (2-fluoro-butan-1-ol) identified at the SMD/MN15/aug-cc-pVTZ//MN15/cc-pVTZ level of theory in water and octanol medium. S32

5.3.12 Table S23. Relative Gibbs energy and populations of the energetic minima of **G3** (3-fluoro-butan-1-ol) identified at the SMD/MN15/aug-cc-pVTZ//MN15/cc-pVTZ level of theory in water and octanol medium. S33

6 Synthesis S34

6.1 General methods. S34

6.2 Compounds available. S34

6.3 Synthesis of novel compounds S34

6.3.1 Synthesis of 3,3-difluoro-2-methylpropan-1-ol (**D4**) S34

6.3.2 Synthesis of (1-fluorocyclopropyl)methanol (**E2**) S35

6.3.3 Synthesis of (*rac*-(1*R*,2*R*)-2-fluorocyclopropyl)methanol (**E3**) S35

6.3.4 Synthesis of (*rac*-(1*R*,2S)-2-fluorocyclopropyl)methanol (**E4**) S36

6.3.5 Synthesis of (2,2-difluorocyclopropyl)methanol (**E5**) S36

7 NMR spectra for novel compounds S38

7.1 3,3-Difluoro-2-methylpropan-1-ol (**D4**) S38

7.1.1 3,3-Difluoro-2-methylpropan-1-ol (**D4**) (^{1}H NMR, $CDCl_3$, 400 MHz) S38

7.1.2 3,3-Difluoro-2-methylpropan-1-ol (**D4**) (^{19}F NMR, $CDCl_3$, 376 MHz) S39

7.1.3 3,3-Difluoro-2-methylpropan-1-ol (**D4**) (^{19}F{^{1}H} NMR, $CDCl_3$, 376 MHz) S40

7.1.4 3,3-Difluoro-2-methylpropan-1-ol (**D4**) (^{13}C NMR, $CDCl_3$, 101 MHz) S41

7.2 (1-Fluorocyclopropyl)methanol (**E2**) S42

7.2.1 (1-Fluorocyclopropyl)methanol (**E2**) (^{1}H NMR, $CDCl_3$, 400 MHz) S42

7.2.2 (1-Fluorocyclopropyl)methanol (**E2**) (^{13}C NMR, $CDCl_3$, 101 MHz) S43

7.2.3 (1-Fluorocyclopropyl)methanol (**E2**) (^{19}F NMR, $CDCl_3$, 376 MHz) S44

7.2.4 (1-Fluorocyclopropyl)methanol (**E2**) (^{19}F {1H} NMR, $CDCl_3$, 376 MHz) S45

7.3 *rac*-(1*R*,2*R*)-(2-Fluorocyclopropyl)methanol (**E3**) S46

7.3.1 *rac*-(1*R*,2*R*)-2-Fluorocyclopropyl)methanol (**E3**) (^{1}H NMR, $CDCl_3$, 400 MHz) S46

7.3.2 *rac*-(1*R*,2*R*)-(2-Fluorocyclopropyl)methanol (**E3**) (^{13}C NMR, $CDCl_3$, 126 MHz) S47

7.3.3 *rac*-(1*R*,2*R*)-(2-Fluorocyclopropyl)methanol (**E3**) (^{19}F NMR, $CDCl_3$, 471 MHz) S48

7.3.4 *rac*-(1*R*,2*R*)-(2-Fluorocyclopropyl)methanol (**E3**) (^{19}F {1H} NMR, $CDCl_3$, 471 MHz) S49

7.4 *rac*-(1*R*,2*S*)-(2-fluorocyclopropyl)methanol (E4) S50

7.4.1 *rac*-(1*R*,2*S*)-(2-fluorocyclopropyl)methanol (**E4**) (^{1}H NMR, $CDCl_3$, 400 MHz) S50

7.4.2 *rac*-(1*R*,2*S*)-(2-fluorocyclopropyl)methanol (**E4**) (^{13}C NMR, $CDCl_3$, 101 MHz) S51

7.4.3 *rac*-(1*R*,2*S*)-(2-fluorocyclopropyl)methanol (**E4**) ^{19}F NMR, $CDCl_3$, 471 MHz S52

7.4.4 *rac*-(1*R*,2*S*)-(2-fluorocyclopropyl)methanol (**E4**) ^{19}F {1H} NMR, $CDCl_3$, 471 MHz S53

1 Comparison between linear, isopropyl and cyclopropyl (Figure S1)

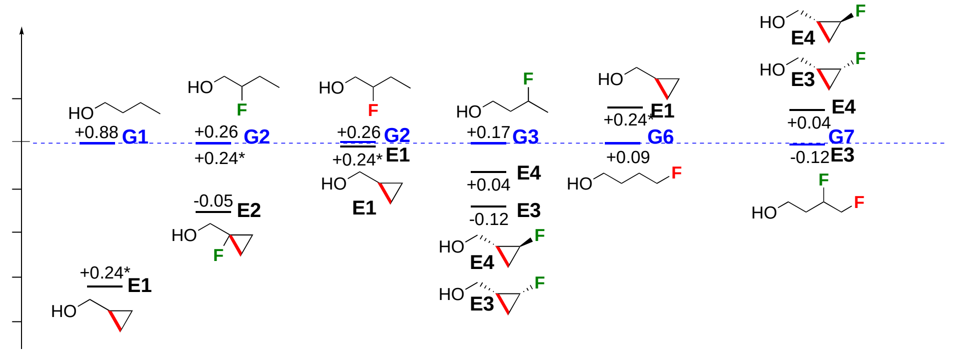

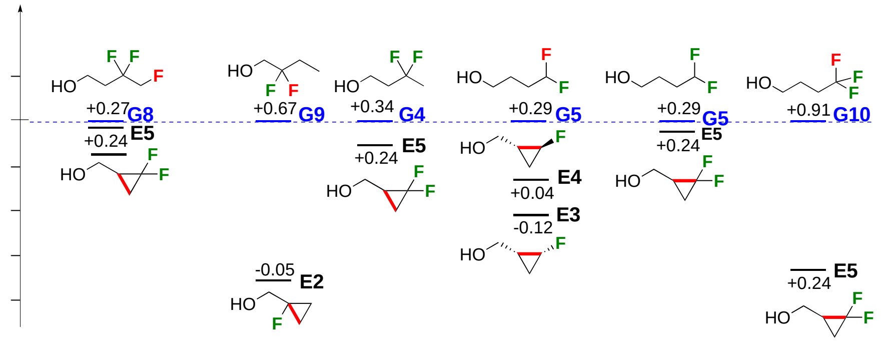

Full Figure:

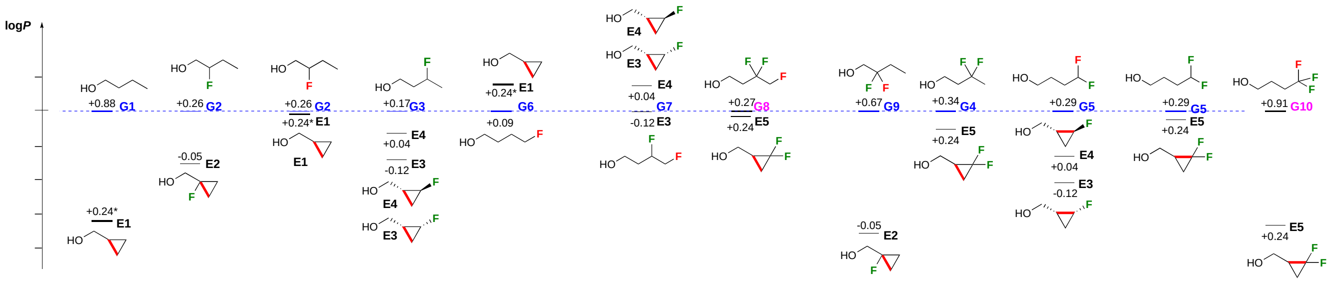

2 Determination of the clog*P* of the parent compounds cyclopropylmethanol E1 and (3-oxetanyl)methanol F1

No experimental log*P* values of these compounds are available in the literature. Instead, their values were estimated using the correlation between published experimental log*P* values for various alkanols, and their calculated values using various prediction tools. Experimental log*P* was plotted against c*log*P for each predictor tool, and a linear regression performed. This provided an equation to obtain an estimated log*P* based on that calculator's clog*P* value.

2.1 Experimental log*P* values[a] for selected nonfluorinated alkanols (Table S1)

Compound	log*P*	Compound	log*P*
**methanol**	-0.77	**3-hexanol**	1.65
**ethanol**	-0.31	**cyclohexanol**	1.23
**1,2-ethanediol**	-1.36	**1-heptanol**	2.72
**1-propanol**	0.25	**2-heptanol**	2.31
**2,2-dimethylpropan-1-ol**	1.31	**3-heptanol**	2.24
**2-propanol**	0.05	**4-heptanol**	2.22
**1,3-propanediol**	-1.04	**1-octanol**	3.00
**1-butanol**	0.88	**2-octanol**	2.90
**2-butanol**	0.61	**4-octanol**	2.68
***tert*-butanol**	0.35	**3,3-dimethyl-2-butanol**	1.48
**1,4-butanediol**	-0.83	**3-methyl-1-butanol**	1.16
**1-pentanol**	1.56	**3-methyl-2-butanol**	1.28
**2-pentanol**	1.19	**2-methyl-2-butanol**	0.89
**3-pentanol**	1.21	**2,3-butanediol**	-0.92
**1-hexanol**	2.03	**2-methylpropan-1-ol**	0.76
**2-hexanol**	1.76	**1,2-propanediol**	-0.92

[a] C. Hansch, A. Leo, and D. Hoekman, *Exploring QSAR: Hydrophobic, Electronic, and Steric Constants*, American Chemical Society, 1995

2.2 Clog*P* values for selected nonfluorinated alkanols (Table S2)

Compound	Molinspiration[a]	Biobyte[b]	Marvin sketch[c]	ACD log*P*[d]	AZ $logD_{7.4}$[e]
**methanol**	-0.32	-0.76	-0.57	-0.72	-0.43
**ethanol**	0.06	-0.24	-0.22	-0.19	0.10
**1,2-ethanediol**	-0.95	-1.37	-1.31	-1.69	-0.62
**1-propanol**	0.56	0.29	0.25	0.34	0.64
**2,2-dimethylpropan-1-ol**	1.38	1.09	1.15	1.04	1.32
**2-propanol**	0.42	0.07	0.19	0.16	0.40
**1,3-propanediol**	-0.68	-1.04	-1.26	-1.04	-0.32
**1-butanol**	1.12	0.82	0.64	0.88	1.20
**2-butanol**	0.92	0.60	0.66	0.69	0.87
***tert*-butanol**	0.87	0.47	0.27	0.51	0.57
**1,4-butanediol**	-0.41	-1.16	-0.81	-1.02	-0.20
**1-pentanol**	1.62	1.35	1.04	1.41	1.42
**2-pentanol**	1.48	1.13	1.06	1.22	1.35
**3-pentanol**	1.43	1.13	1.13	1.22	1.20
**1-hexanol**	2.13	1.88	1.43	1.94	1.70
**2-hexanol**	1.99	1.66	1.45	1.75	1.84
**3-hexanol**	1.98	1.66	1.52	1.75	1.69
**cyclohexanol**	1.59	1.27	1.02	1.34	1.32
**1-heptanol**	2.63	2.41	1.83	2.47	2.05
**2-heptanol**	2.49	2.19	1.85	2.29	2.23
**3-heptanol**	2.49	2.19	1.92	2.29	2.20
**4-heptanol**	2.54	2.19	1.92	2.29	2.18
**1-octanol**	3.14	2.94	2.23	3.00	2.43
**2-octanol**	3.00	2.72	2.24	2.82	2.63

Compound	Molinspiration[a]	Biobyte[b]	Marvin sketch[c]	ACD log$P$[d]	AZ log$D_{7.4}$[e]
**4-octanol**	3.05	2.72	2.32	2.82	2.73
**3,3-dimethyl-2-butanol**	1.75	1.40	1.57	1.39	1.53
**3-methyl-1-butanol**	1.33	1.22	0.97	1.22	1.22
**3-methyl-2-butanol**	1.17	1.00	1.06	1.04	1.14
**2-methyl-2-butanol**	1.37	1.00	0.74	1.04	0.87
**2,3-butanediol**	-0.23	-0.75	-0.48	-0.99	0.10
**2-methylpropan-1-ol**	0.80	0.69	0.65	0.69	1.06
**1,2-propanediol**	-0.59	-1.06	-0.90	-1.34	-0.38

[a] molinspector: http://www.molinspiration.com/cgi-bin/properties

[b] Daylight/Biobyte ClogP Pomona College and BioByte, Inc., Claremont, CA (bio-loom version 1.6, program version 5: http://www.biobyte.com/)

[c] MarvinSketch – ChemAxon: https://docs.chemaxon.com/display/docs/logP+Plugin#logPPlugin-Method.

[d] **Version: 2018.1.1** ACDlogP was calculated using ACD/Labs software, version 2018 (see www.acdlabs.com).

[e] AstraZeneca internal method.

2.3 Correlation of clog*P* with experimental data for nonfluorinated alkanols (Figures S2–S6)

Each set of clog*P* values from the individual calculators were plotted against the corresponding experimental values. The correlation was then determined for each data set.

2.3.1 Molinspiration (Figure S2)

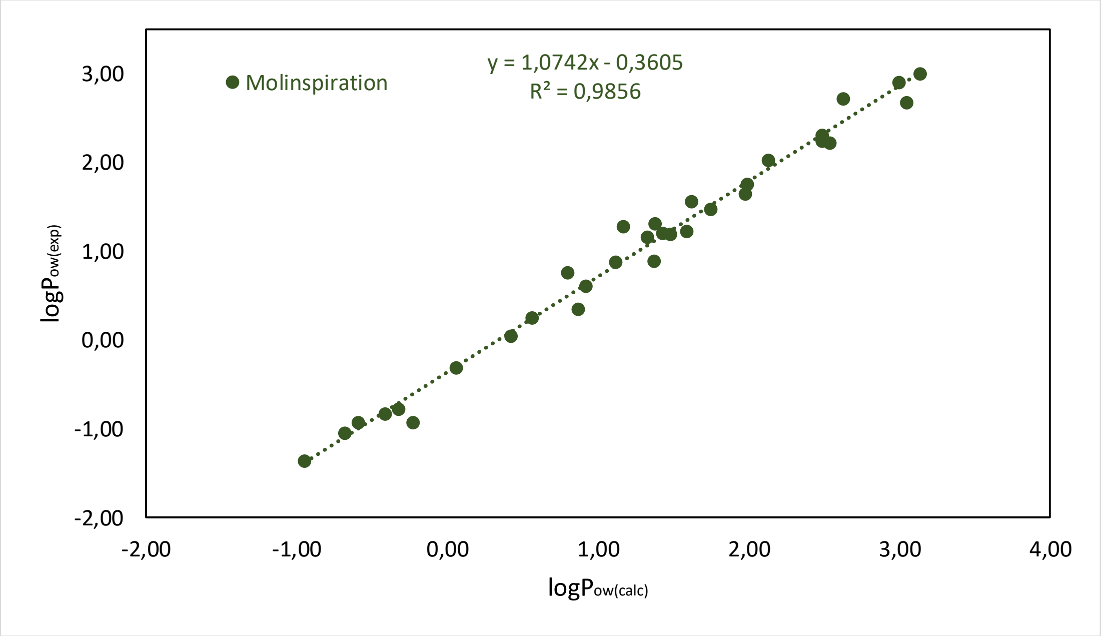

Figure S2: Correlation of the experimental data with clog*P* values obtained with Molinspiration.

2.3.2 Biobyte (Figure S3)

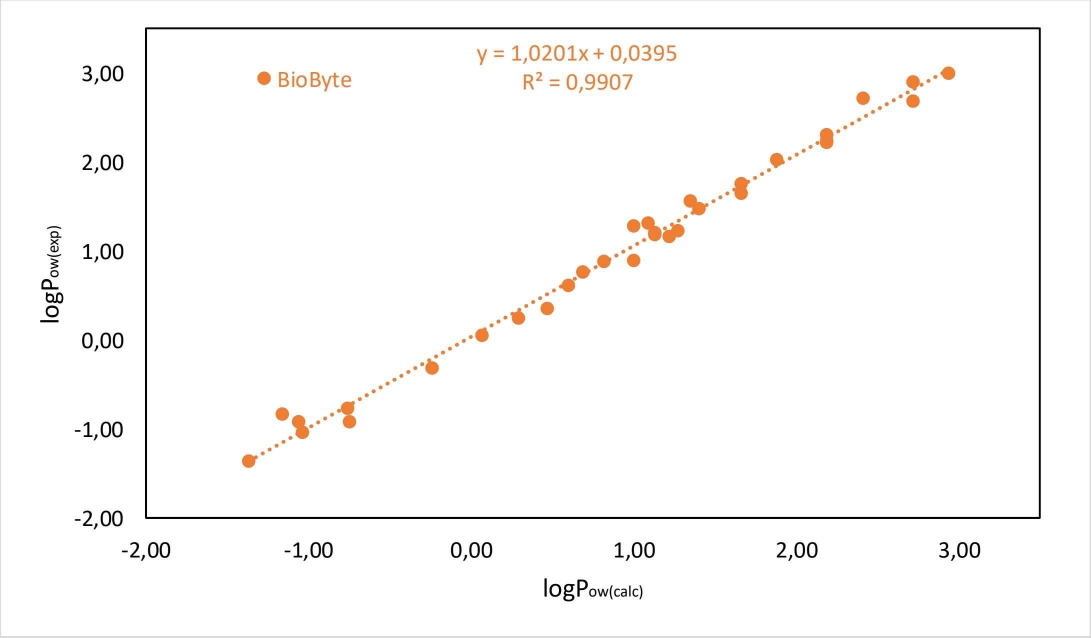

Figure S3: Correlation of the experimental data with clog*P* values obtained with Biobyte.

2.3.3 Marvinsketch (Figure S4)

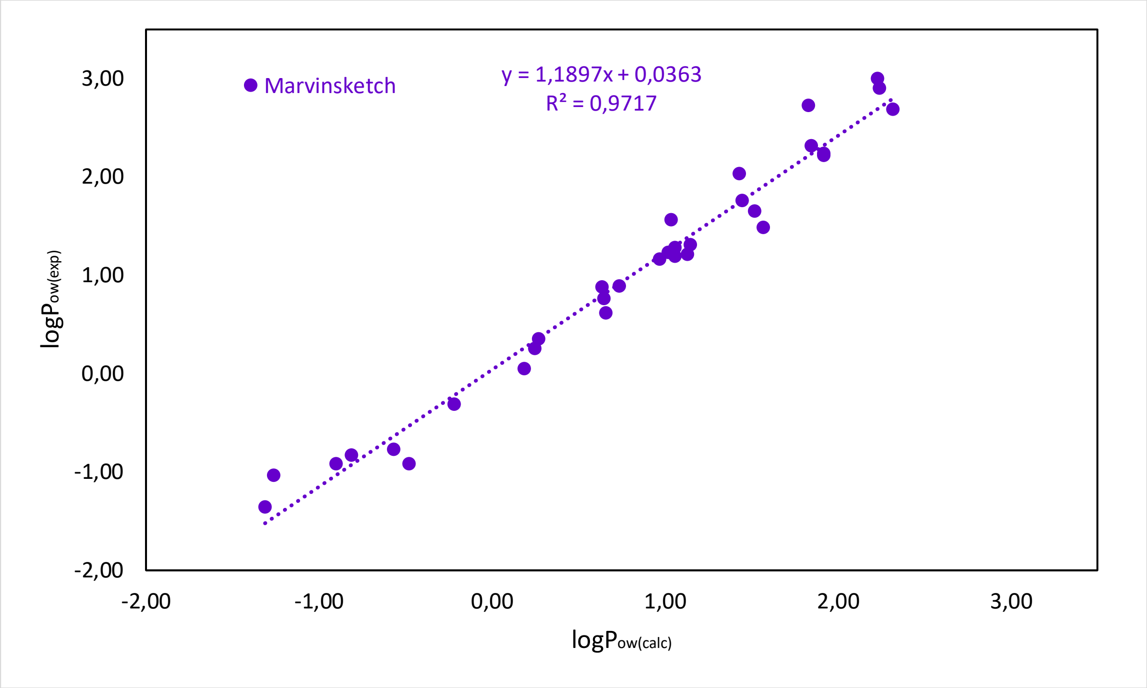

Figure S4: Correlation of the experimental data with clog*P* values obtained with Marvinsketch.

2.3.4 ACDlog*P* (Figure S5)

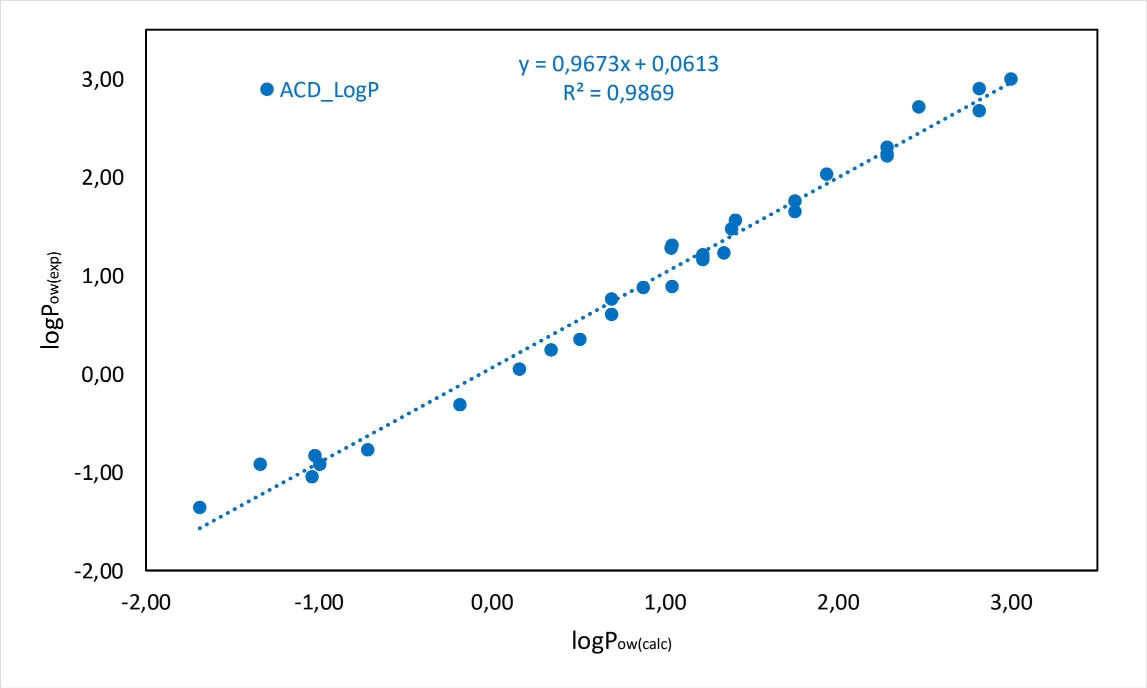

Figure S5: Correlation of the experimental data with clog*P* values obtained with ACDlogP.

2.3.5 AZlogD$_{7.4}$ (Figure S6)

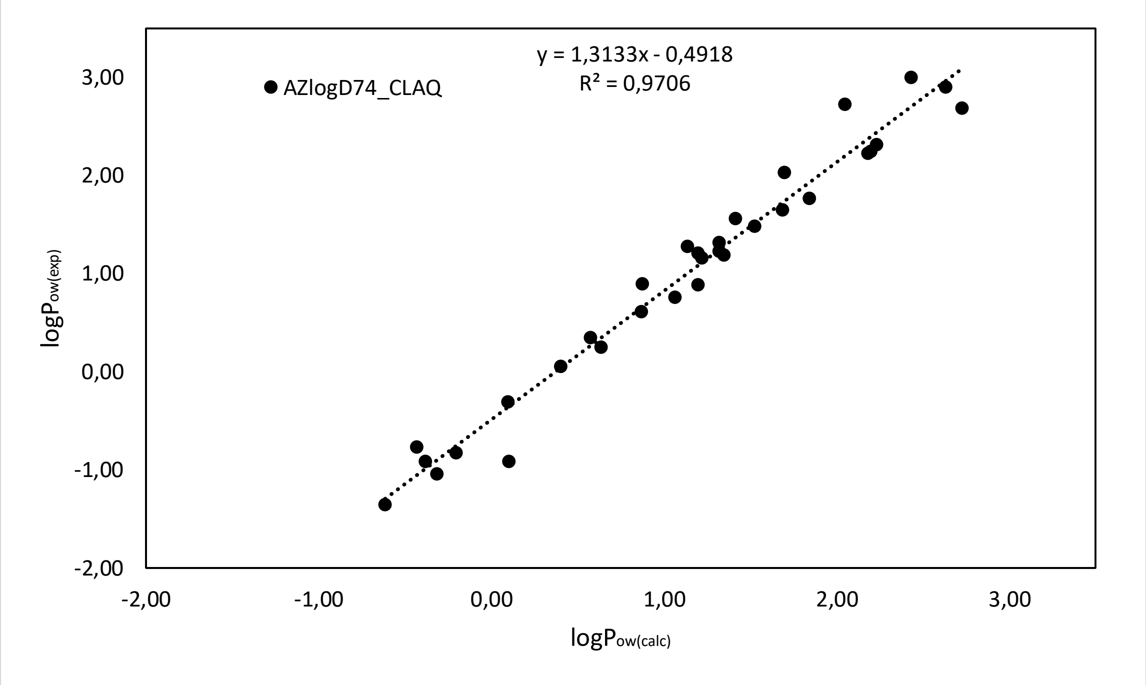

Figure S6: Correlation of the experimental data with clog*D* values obtained with the internal AstraZeneca log*D* calculation programme.

2.3.6 Trendlines (Table S3)

Predictor	Trendline equation	R^2 value
Molinspiration	$y = 1.0742x - 0.3605$	0.9856
Biobyte	$y = 1.0201x + 0.0395$	0.9907
Marvinsketch	$y = 1.1897x + 0.0363$	0.9717
ACDlog*P*	$y = 0.9673x + 0.0613$	0.9869
AZlogD$_{7.4}$	$y = 1.3133x - 0.4918$	0.9706

These trendline equations were used to estimate the log*P* value of each compound for each prediction tool. All methods showed high predictive power (from R^2 value), but there is considerable variation in the generated straight-line equations, and thus their estimated log*P* values. Therefore, the mean average across all methods was used as the final estimated value.

The standard deviation (σ) was calculated using the formula:

$$\sigma = \sqrt{\frac{1}{N}\sum_{i=1}^{N}(x_i - \bar{x})^2}$$

Where x_i are the individual estimated log*P* values and $\bar{x}$ is the mean average.

2.4 Estimated log*P* values: Cyclopropylmethanol (Table S4)

Predictor	Calculated log*P*	Estimated log*P*
Molinspiration	0.55	0.23
Biobyte	0.21	0.25
Marvinsketch	0.14	0.20
ACDlog*P*	0.18	0.24
AZlog$D_{7.4}$	0.60	0.30

The average of the estimated values was 0.24, and the standard deviation was 0.03.

The log*P* of cyclopropylmethanol was taken to be 0.24 ± 0.03.

2.5 Estimated log*P* values: (3-oxetanyl)methanol (Table S5)

Predictor	Calculated log*P*	Estimated log*P*
Molinspiration	-0.10	-0.47
Biobyte	-0.61	-0.58
Marvinsketch	-0.82	-0.94
ACDlog*P*	-1.15	-1.05
AZlog$D_{7.4}$	-0.36	-0.96

The average of the estimated values was −0.80, and the standard deviation was 0.23.

The log*P* of (3-oxetanyl)methanol was taken to be −0.80 ± 0.23.

3 clog*P* values of the fluorinated derivatives and correlation with the experimental values

3.1 Table with the clog*P* values of five calculation methods (Table S6)

Compound (exp log*P*)		Molins piration[a]	Biobyte[b]	Marvin sketch[c]	ACD log*P*[d]	AZ $logD_{7.4}$[e]
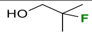	**D2** (0.10)	0.79	0.42	0.09	0.23	0.54
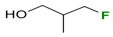	**D3** (0.29)	0.72	0.12	0.15	0.18	0.74
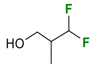	**D4** (0.58)	1.00	0.14	0.34	0.06	0.84
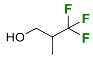	**D5** (0.98)	1.35	0.96	1.06	0.50	1.24
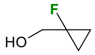	**E2** (-0.05)	0.41	0.05	-0.27	-0.26	0.40
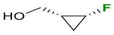	**E3** (-0.12)	0.58	-0.02	-0.27	-0.41	0.25
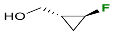	**E4** (0.04)	0.58	-0.02	-0.27	-0.41	0.25
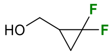	**E5** (0.24)	0.93	-0.11	0.23	-0.74	0.58
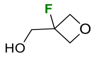	**F2** (-0.73)	-0.12	0.13	-0.94	-0.89	-0.04
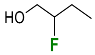	**G2** (0.26)	0.84	0.55	0.48	0.41	0.62
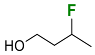	**G3** (0.17)	0.61	0.25	0.06	0.18	0.41
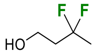	**G4** (0.34)	0.97	0.27	0.55	0.06	0.62
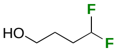	**G5** (0.29)	0.79	-0.03	-0.01	-0.15	0.76
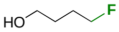	**G6** (0.09)	0.52	0.10	0.10	0.17	0.51
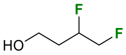	**G7** (-0.12)	0.53	0.08	-0.12	-0.29	0.27
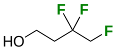	**G8** (0.27)	0.89	0.38	0.67	-0.17	0.75
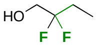	**G9** (0.67)	1.20	0.87	0.88	0.52	0.83
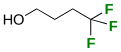	**G10** (0.91)	1.15	0.38	0.90	0.10	1.07

[a] molinspector: http://www.molinspiration.com/cgi-bin/properties

[b] Daylight/Biobyte ClogP Pomona College and BioByte, Inc., Claremont, CA (bio-loom version 1.6, program version 5: http://www.biobyte.com/)

[c] MarvinSketch – ChemAxon: https://docs.chemaxon.com/display/docs/logP+Plugin#logPPlugin-Method.

[d] **Version: 2018.1.1** ACDlogP was calculated using ACD/Labs software, version 2018 (see www.acdlabs.com).

[e] AstraZeneca internal method.

3.2 Correlation of clog*P* with experimental data (Figures S7-S11)

3.2.1 Molinspiration (Figure S7)

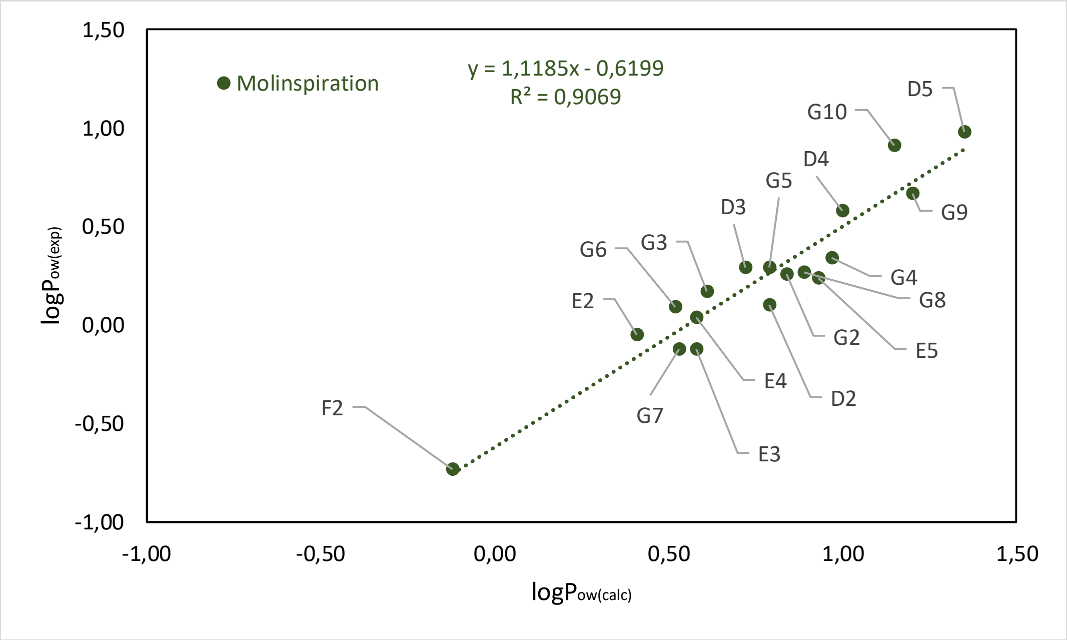

Figure S7. Correlation of the experimental data with clog*P* values obtained with Molinspiration.

3.2.2 Biobyte (Figure S8)

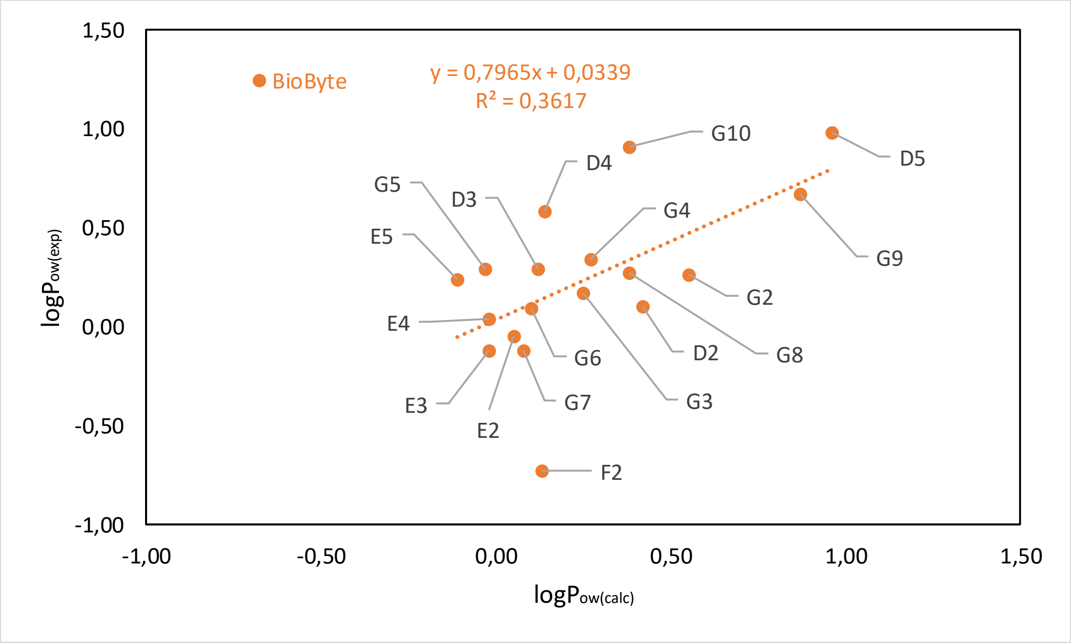

Figure S8. Correlation of the experimental data with clog*P* values obtained with Biobyte.

3.2.3 Marvinsketch (Figure S9)

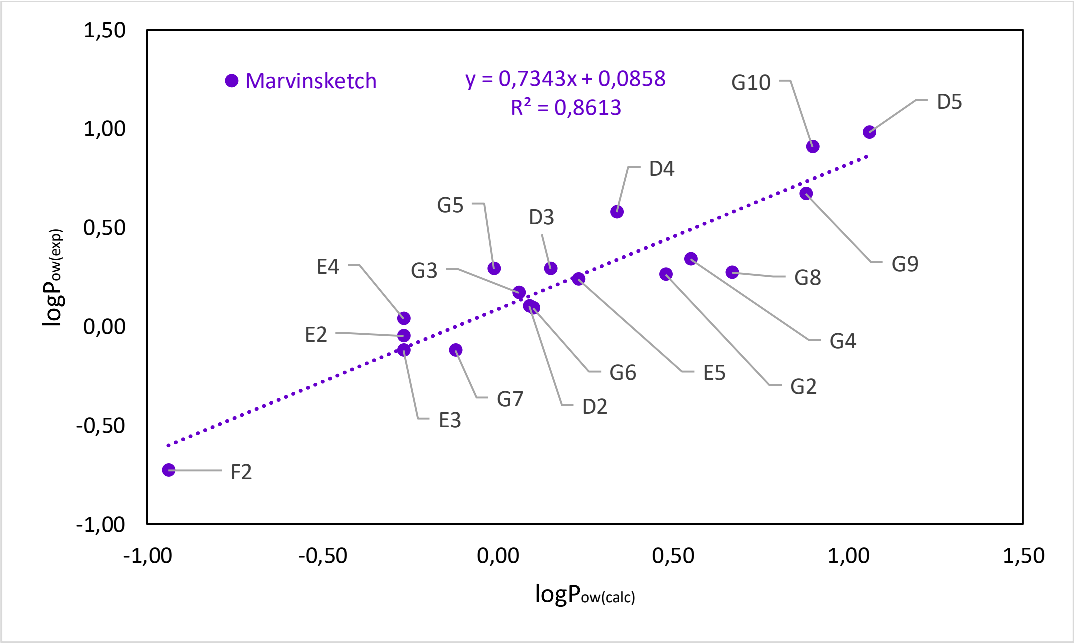

Figure S9. Correlation of the experimental data with clog*P* values obtained with Marvinsketch.

3.2.4 ACDlogP (Figure S10)

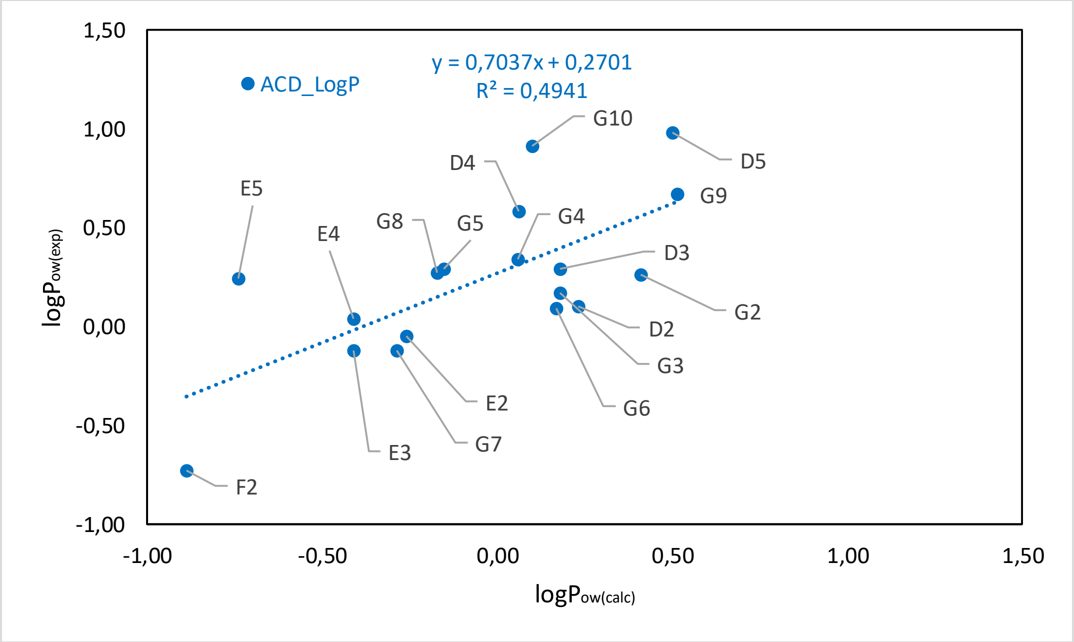

Figure S10. Correlation of the experimental data with clog*P* values obtained with ACDlogP.

3.2.5 AZlogD$_{7.4}$ (Figure S11)

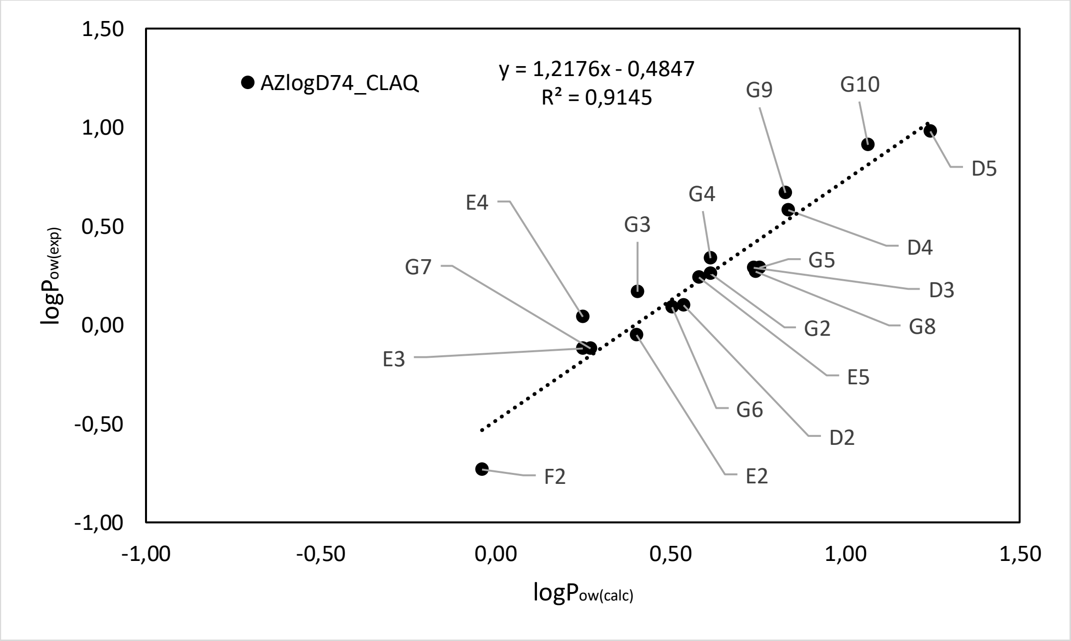

Figure S11: Correlation of the experimental data with clog*P* values obtained with the internal AstraZeneca log*P* calculation programme.

3.2.6 Trendline (Table S7)

Predictor	Trendline equation	R^2 value
Molinspiration	$y = 1.1185x - 0.6199$	$R^2 = 0.9069$
Biobyte	$y = 0.7965x + 0.0339$	$R^2 = 0.3617$
Marvinsketch	$y = 0.7343x + 0.0858$	$R^2 = 0.8613$
ACDlogP	$y = 0.7037x + 0.2701$	$R^2 = 0.4941$
AZlogD$_{7.4}$	$y = 1.2176x - 0.4847$	$R^2 = 0.9145$

The AZlogD$_{7.4}$ method gives the best predictive power (highest R^2 value), followed closely by Molinspiration.

4 Experimental log*P* data

4.1 Determination of log*P*

Lipophilicities of the fluorinated alkanols were determined using a previously published protocol:[1] to a 10 mL pear-shaped flask was added the compound (1.0–10 mg) for log*P* determination, the reference compound (1.0–10 mg, with known log*P* value, e.g., 2,2,2-trifluoroethanol, log*P*: +0.36), water (2 mL) and *n*-octanol (2 mL). The resulting biphasic mixture was stirred (at 600 rpm) for 2 h at 25 °C, and then left without stirring for 16 h at 25 °C to allow phase separation. An aliquot of 0.5 mL was taken from each phase using 1 mL syringes with long needles and added to two separate NMR tubes. A deuterated NMR solvent (0.1 mL, e.g., acetone-d_6), or a capillary tube containing deuterated NMR solvent, was added to the NMR tubes to enable signal locking. Because of the volatility of the used compounds, the NMR tubes were sealed using a blowtorch. For NMR samples with directly added deuterated solvent, the tubes were inverted 20 times for mixing. For $^{19}F\{^1H\}$ NMR experiments, NMR parameters were set as follows: D1 30 s for the octanol sample, D1 60 s for the water sample; and O1P centered between two diagnostic fluorine peaks. If needed, an increased number of transients (NS) and/or narrower spectral window (SW) for a good S/N ratio (typically >300) was applied. After NMR data processing, integration ratios ρ_{oct} and ρ_{aq} (ρ_{oct} is defined as the integration ratio between the compound and the reference compound in the octanol sample; likewise, for ρ_{aq}) were obtained, and used in the equation ($\log P^{X} = \log P^{ref} + \log(\rho_{oct}/\rho_{aq})$) to obtain the log*P* value of the compound. The log*P* measurement of each compound was run in triplicate. Log*P* values of non-fluorinated compounds were taken from the literature.

Calculation of standard deviation was carried out using the equation shown on p S11.

4.2 Standard NMR settings

Standard NMR parameter setting: SW, 300 ppm; centered O1P; NS 64; D1 30 sec (octanol sample), D1 60 sec (water sample). Any change from standard setting was described for each compound. Unless specified otherwise 2,2,2-trilfuoroethanol (log*P*: +0.36) was used as the reference compound.

4.3 Tables S8-S10: data of all individual measurements

4.3.1 Isobutanol series (Table S8)

Compound	Nr	Experiments (octanol/water)	ρ_{oct}/ ρ_{wat}	log*P*	Average log*P*	Error
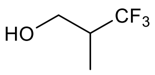	**D5** *	se0419zw1/ se0419zw2	1.3020/ 0.3142	+0.977	+0.98	+0.978 (±0.001)
		se0419zw4/ se0419zw5	1.3174/ 0.3175	+0.978		
		se0419zw6/ se0419zw7	1.2874/ 0.3095	+0.979		
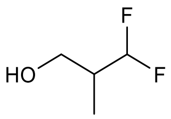		se0319zw1/ se0319zw2	0.9798/ 0.5859	+0.583	+0.58	+0.583 (±0.005)
	**D4** **	se0319zw3/ se0319zw4	0.9903/ 0.6016	+0.576		
		se0319zw5/ se0319zw6	0.9900/ 0.5845	+0.589		
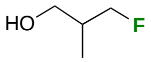	**D3**	au2616bj7/ au2616bj8	0.2390/ 0.2842	+0.28	+0.29	+0.289 (±0.004)
		au2616bj9/ au2616bj10	0.3091/ 0.3649	+0.29		
		au2616bj11/ au2616bj12	0.1631/ 0.1898	+0.29		
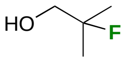	**D2**	au2416bj7/ au2416bj8	0.1647/ 0.2919	+0.11	+0.10	+0.104 (±0.006)
		au2416bj9/ au2416bj10	0.1587/ 0.2902	+0.10		
		au2416bj12/ au2416bj11	0.4046/ 0.7306	+0.10		

*Change from standard in NMR parameter setting: SW (120 ppm); octanol sample, D1 (20 sec); water sample, D1 (40 sec).

**Change from standard in NMR parameter setting: SW (200 ppm); octanol sample, D1 (20 sec); water sample, D1 (40 sec).

4.3.2 Cyclopropylmethanol series (Table S9)

Compound	Nr	Experiment (octanol/water)	r_o/r_w	log*P*	Average log*P*	error
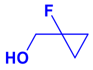	**E2**	ma0818bj16/ ma0818bj17	0.3196/ 0.8280	-0.05	-0.05	-0.054 (±0.004)
		ma0818bj18/ ma0818bj19	0.2721/ 0.7142	-0.06		
		ma0818bj20/ ma0818bj21	0.3051/ 0.7854	-0.05		
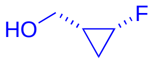	**E3**	ma0918bj12/ ma0918bj13	0.1806/ 0.5405	-0.12	-0.12	-0.123 (±0.005)
		ma0918bj14/ ma0918bj15	0.2152/ 0.6618	-0.13		
		ma0918bj16/ ma0918bj17	0.2742/ 0.8391	-0.13		
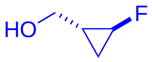	**E4**	ma0918bj6/ ma0918bj7	2.8391/ 0.4521	+0.05	+0.04	+0.042 (±0.004)
		ma0918bj8/ ma0918bj9	2.6565/ 0.4317	+0.04		
		ma0918bj10/ ma0918bj11	2.4055/ 0.3919	+0.04		
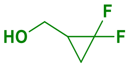	**E5**	ma0818bj9/ ma0818bj10	0.6074/ 0.8029	+0.24	+0.24	+0.242 (±0.004)
		ma0818bj11/ ma0818bj12	0.6010/ 0.7946	+0.24		
		ma0818bj13/ ma0818bj14	0.6163/ 0.7988	+0.25		

P2 and **P3** – Reference compound: 2-fluoroethan-1-ol (log*P*: -0.75)

4.3.3 Oxetan-3-ylmethanol series (Table S10)

Compound	Nr	Experiment (octanol/water)	r_o/r_w	log*P*	Average log*P*	error
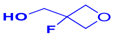	**F2**	au2416bj13/ au2416bj14	0.2429/ 2.8355	- 0.70	-0.73	-0.729 (±0.016)
		au2416bj15/ au2416bj16	0.1157/ 1.4632	- 0.74		
		au2416bj17/ au2416bj18	0.2416/ 3.0274	- 0.74		

4.4 Example of NMR spectra for log*P* determination, with detailed NMR parameter information and integration details after reprocessing

The figures below show the initial and processed spectra of 3,3-difluoro-2-methylpropan-1-ol (**D4**)

4.4.1 Octanol sample (Figure S12)

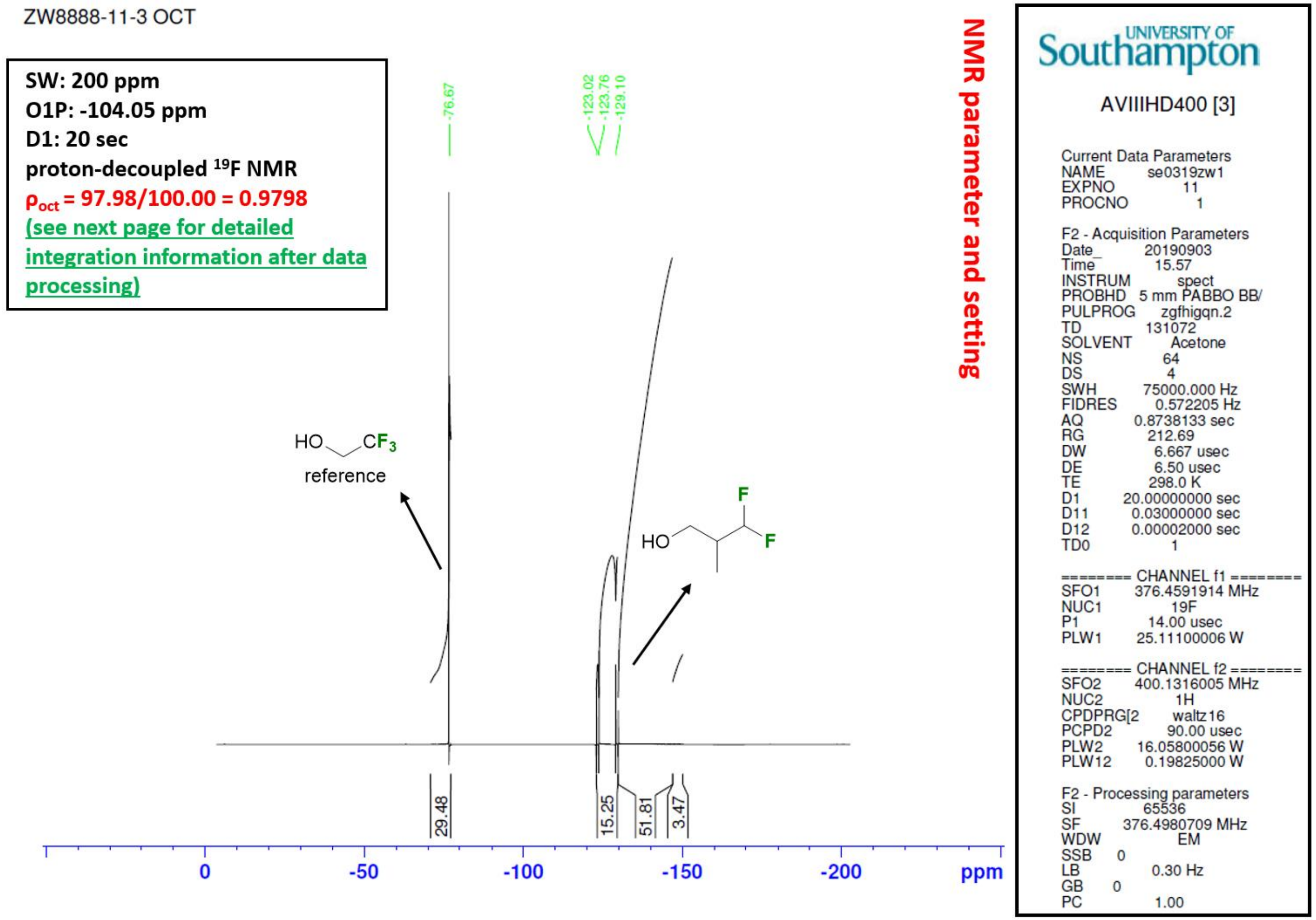

Acquisition Time (sec)	0.8738	***Comment***	ZW8888-11-3 OCT			***Date***	03 Sep 2019 15:58:08
Date Stamp	03 Sep 2019 15:58:08						
File Name	\\soton.ac.uk\ude\personalfiles\users\zw1m12\mydesktop\ZW8888 Labbook\ZW8888-11\se0319zw1\11\fid					***Frequency (MHz)***	376.46
Nucleus	19F	***Number of Transients***	64	***Origin***	spect	***Original Points Count***	65536
Owner	nmr	***Points Count***	262144	***Pulse Sequence***	zgfhigqn.2	***Receiver Gain***	212.69
SW(cyclical) (Hz)	75000.00	***Solvent***	Acetone	***Spectrum Offset (Hz)***	-39174.6172	***Spectrum Type***	STANDARD
Sweep Width (Hz)	74999.71	***Temperature (degree C)***	24.996				

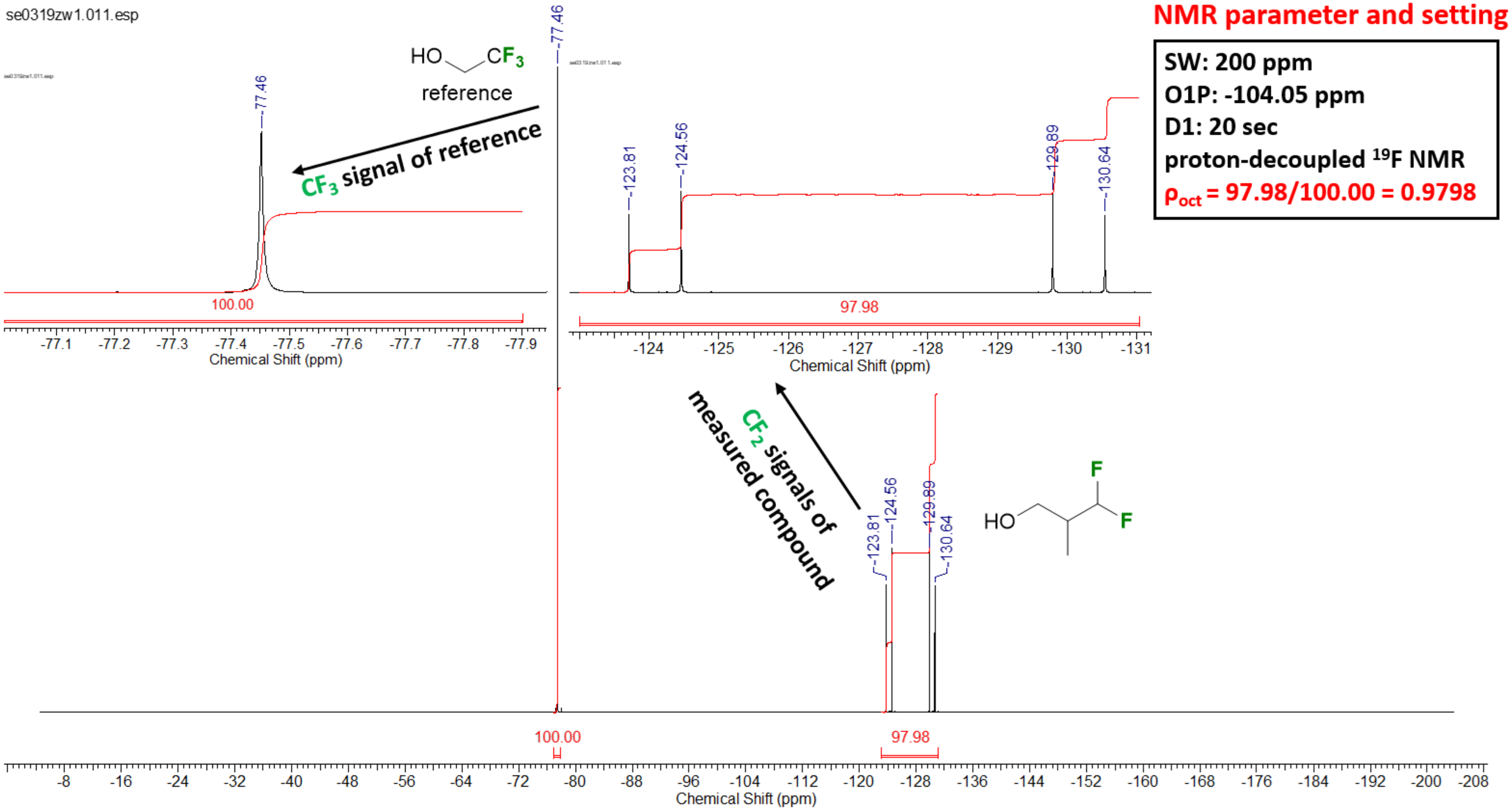

4.4.2 Water sample (Figure S13)

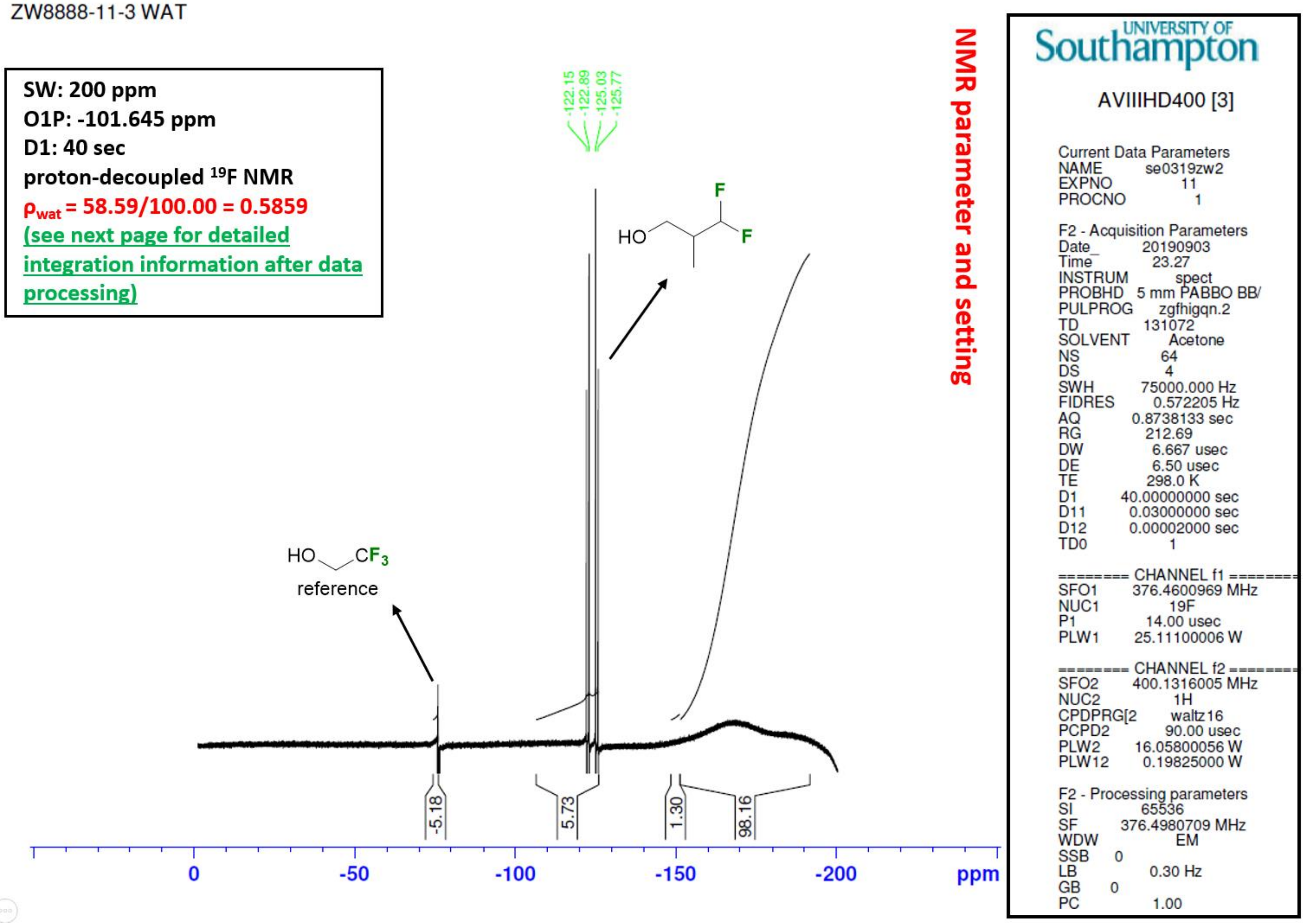

Acquisition Time (sec)	0.8738	***Comment***	ZW8888-11-3 WAT			***Date***	03 Sep 2019 23:28:16
Date Stamp	03 Sep 2019 23:28:16						
File Name	\\soton.ac.uk\ude\personalfiles\users\zw1m12\mydesktop\ZW8888 Labbook\ZW8888-11\se0319zw2\11\fid					***Frequency (MHz)***	376.46
Nucleus	19F	***Number of Transients***	64	***Origin***	spect	***Original Points Count***	65536
Owner	nmr	***Points Count***	262144	***Pulse Sequence***	zgfhigqn.2	***Receiver Gain***	212.69
SW(cyclical) (Hz)	75000.00	***Solvent***	Acetone	***Spectrum Offset (Hz)***	-38269.1406	***Spectrum Type***	STANDARD
Sweep Width (Hz)	74999.71	***Temperature (degree C)***	25.005				

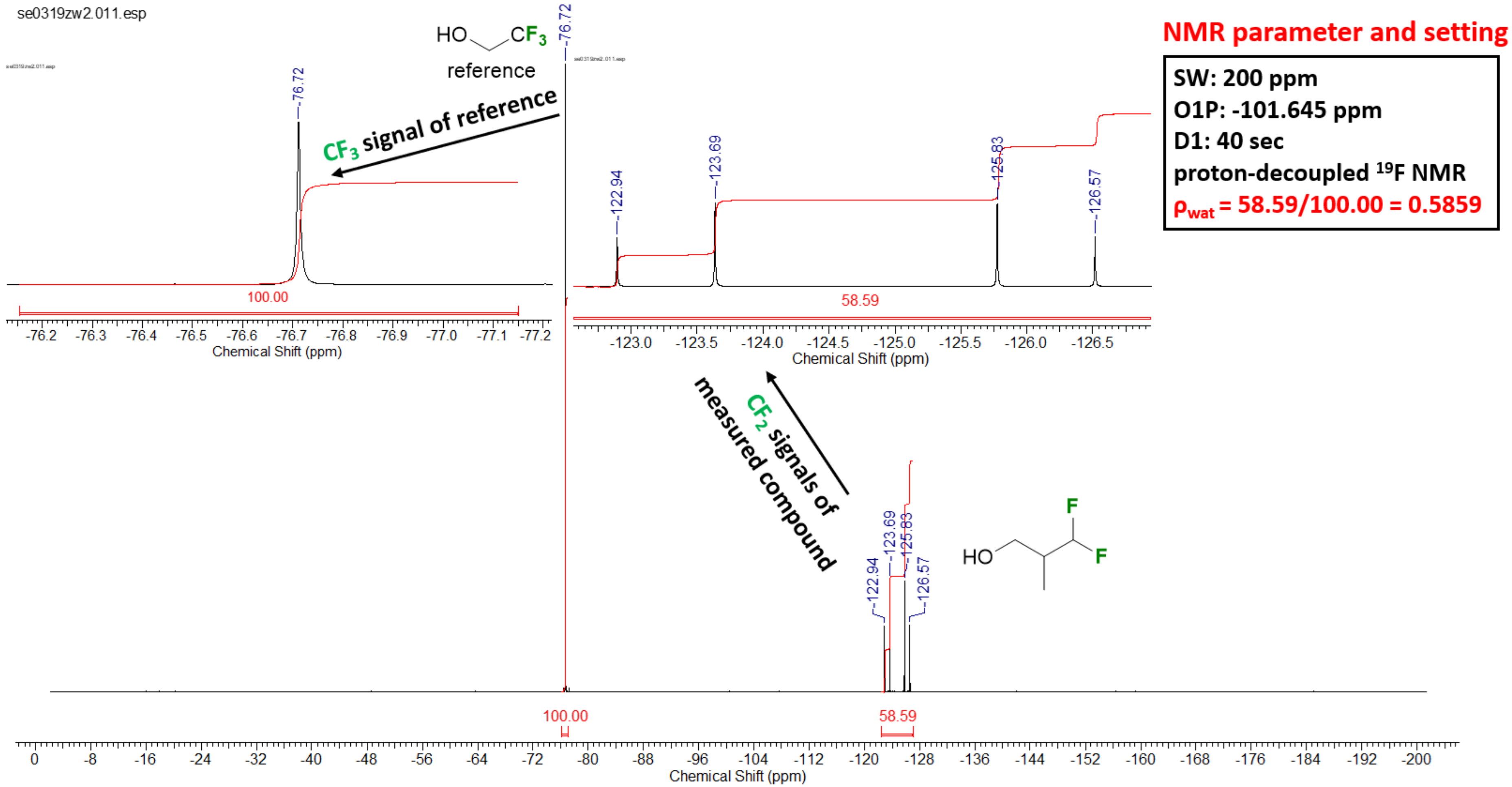

5 Conformational analysis in water and *n*-octanol medium.

5.1 General

Analogous as described in ref [7], the theoretical calculations were carried out with the Gaussian16 program.[2] The conformational analysis of the various compounds investigated was performed with the MN15 functional[3, 4] in combination with the triple-zeta quality aug-cc-pVTZ basis set. Scans along the various rotatable bonds: C–C, C–O, and O–H bonds of the compounds have systematically been conducted. The solvent effects (octanol and water) were taken into account using the SMD solvation continuum model.[5] The vibrational spectrum of each optimized conformer was computed to confirm its nature of true minimum and to obtain the correction to the free energies. Single point calculations at the SMD/MN15/aug-cc-pVTZ were finally carried out to obtain refined electronic energy values. The relative populations, p_i, of the various conformers were evaluated at 298 K from the computed free energies through a Boltzmann distribution:

$$p_i = \frac{e^{-\Delta G_i/RT}}{\sum_{i=1}^{n} e^{-\Delta G_i/RT}}$$

The theoretical molecular dipole moments were then computed for each conformer, and were weighted according to these populations (either for the compound as a whole or within a conformer series as in Tables 1,2). The fluorohydrin lipophilicity was then estimated from the above results through the calculations of the weighted SMD Gibbs energies obtained in water and in *n*-octanol at 298.15 K to obtain the standard free energy associated with the transfer between these two solvents. The octanol/water partition coefficient was then calculated according to Eq (2), and following the procedure proposed by Ribeiro for chloroform/water partition coefficient calculations:[6]

5.2 All values (Table S11)

$$\log P = -\frac{\Delta G^{o}_{o/w}}{2.303RT} = -\frac{G^{o}_{o} - G^{o}_{w}}{2.303RT}$$

Compound	logP_{exp}	logP_{theor}
**D1**	0.76	1.19
**D2**	0.1	1.2
**D3**	0.29	1.18
**D4**	0.58	1.26
**D5**	0.98	1.52
**E2**	-0.05	0.95
**E3**	-0.12	0.97
**E4**	0.04	0.96
**E5**	0.24	1.13
**F2**	-0.73	0.02
**G1**[7]	0.88	1.40
**G2**	0.26	1.39
**G3**	0.17	1.21
**G4**[7]	0.34	1.23
**G7**[7]	-0.12	1.06
**G8**[7]	0.27	1.30

5.3 Conformational analysis: relative energies and populations

For compounds **G1, G4, G7** and **G8**, see ref.[7]

5.3.1 Table S12. Relative Gibbs energy and populations of the energetic minima of **D1** (2-methylpropan-1-ol) identified at the SMD/MN15/aug-cc-pVTZ//MN15/cc-pVTZ level of theory in water and octanol medium.

	**Water**				**Octanol**		
Conformer	ΔG	p_i	μ	Conformer	ΔG	p_i	μ
	kJ mol^{-1}	%	D		kJ mol^{-1}	%	D
**D1_2**	0.0	40.8%	2.33	**D1_1**	0.0	33.6%	2.30
**D1_1**	1.2	25.5%	2.49	**D1_3**	0.6	26.4%	1.98
**D1_3**	2.4	15.5%	2.16	**D1_2**	0.7	24.9%	2.16
**D1_5**	2.5	15.0%	2.46	**D1_5**	3.0	10.2%	2.27
**D1_5bis**	4.6	3.2%	2.34	**D1_5bis**	3.0	4.9%	2.14
			2.36				2.17

5.3.2 Table S13. Relative Gibbs energy and populations of the energetic minima of **D2** (2-fluoro-2-methylpropan-1-ol) identified at the SMD/MN15/aug-cc-pVTZ//MN15/cc-pVTZ level of theory in water and octanol medium.

	**Water**				**Octanol**		
Conformer	ΔG	p_i	μ	Conformer	ΔG	p_i	μ
	kJ mol^{-1}	%	D		kJ mol^{-1}	%	D
**D2_1**	0.0	41.7%	2.47	**D2_1**	0.0	55.4%	2.29
**D2_2**	1.7	21.2%	4.73	**D2_2**	2.4	21.0%	4.41
**D2_3**	0.8	29.9%	4.31	**D2_3**	3.5	13.5%	3.89
**D2_4**	5.8	2.0%	2.19	**D2_4**	4.8	3.9%	2.18
**D2_5**	5.2	5.0%	2.55	**D2_5**	5.5	6.1%	2.38
			3.50				2.95

5.3.3 Table S14. Relative Gibbs energy and populations of the energetic minima of **D3** (3-fluoro-2-methylpropan-1-ol) identified at the SMD/MN15/aug-cc-pVTZ//MN15/cc-pVTZ level of theory in water and octanol medium.

	Water				Octanol		
Conformer	Δ*G*	p_i	μ	Conformer	Δ*G*	p_i	μ
	kJ mol^{-1}	%	D		kJ mol^{-1}	%	D
**D3_9**	0.0	11.0%	4.42	**D3_16**	0.0	11.6%	4.06
**D3_25**	0.6	8.7%	2.56	**D3_14**	0.3	10.4%	2.11
**D3_27**	1.4	6.2%	1.73	**D3_18**	1.0	7.9%	2.07
**D3_23**	1.8	5.3%	4.41	**D3_9**	1.7	5.9%	4.13
**D3_2**	1.9	5.1%	4.27	**D3_7**	1.9	5.4%	2.38
**D3_7**	2.0	4.9%	2.46	**D3_11**	2.2	4.7%	2.04
**D3_18**	2.0	4.9%	2.22	**D3_23**	2.4	4.4%	4.13
**D3_17**	2.0	4.9%	4.54	**D3_26**	2.5	4.3%	3.89
**D3_5**	2.2	4.6%	1.78	**D3_20**	2.6	4.1%	1.90
**D3_26**	2.2	4.6%	4.16	**D3_2**	2.6	4.0%	4.01
**D3_11**	2.3	4.3%	2.21	**D3_5**	2.9	3.6%	1.59
**D3_14**	2.5	4.0%	2.24	**D3_27**	2.9	3.6%	1.59
**D3_16**	2.8	3.6%	4.28	**D3_25**	3.1	3.4%	2.41
**D3_20**	2.8	3.6%	2.10	**D3_10**	3.1	3.3%	3.83
**D3_3**	3.1	3.2%	1.88	**D3_3**	3.2	3.2%	1.64
**D3_10**	3.6	2.6%	4.05	**D3_12**	3.4	2.9%	1.60
**D3_22**	3.6	2.6%	2.65	**D3_22**	3.5	2.8%	2.57
**D3_19**	3.7	2.5%	1.93	**D3_13**	3.5	2.8%	4.02
**D3_21**	3.7	2.4%	4.20	**D3_19**	3.5	2.8%	1.88
**D3_12**	3.7	2.4%	1.81	**D3_21**	3.6	2.7%	3.91
**D3_6**	4.1	2.1%	4.14	**D3_4**	4.0	2.3%	2.24
**D3_13**	4.5	1.8%	4.21	**D3_1**	4.9	1.6%	2.17
**D3_1**	4.5	1.8%	2.29	**D3_6**	5.5	1.3%	3.83
**D3_8**	5.0	1.5%	3.93	**D3_8**	5.9	1.1%	3.64
**D3_4**	5.2	1.3%	2.35				
			3.13				2.85

5.3.4 Table S15. Relative Gibbs energy and populations of the energetic minima of **D4** (3-fluoro-2-methylpropan-1-ol) identified at the SMD/MN15/aug-cc-pVTZ//MN15/cc-pVTZ level of theory in water and octanol medium.

	Water				Octanol		
Conformer	Δ*G* kJ mol^{-1}	p_i %	μ D	Conformer	Δ*G* kJ mol^{-1}	p_i %	μ D
**D4_20**	0.0	9.4%	3.95	**D4_11**	0.0	15.8%	3.74
**D4_11**	0.2	8.8%	3.88	**D4_20**	0.7	12.0%	3.78
**D4_4**	0.3	8.2%	4.13	**D4_13**	0.7	12.0%	3.66
**D4_2**	0.5	7.6%	1.15	**D4_4**	2.2	6.5%	3.99
**D4_26**	1.0	6.3%	1.12	**D4_19**	2.8	5.1%	3.30
**D4_13**	1.5	5.2%	3.85	**D4_16**	2.9	4.8%	3.41
**D4_5**	1.7	4.6%	3.91	**D4_2**	3.0	4.8%	1.32
**D4_1**	1.8	4.5%	3.32	**D4_17**	3.2	4.3%	3.18
**D4_7**	1.9	4.4%	0.77	**D4_8**	3.7	3.6%	3.26
**D4_22**	2.0	4.2%	0.97	**D4_7**	3.8	3.4%	0.87
**D4_16**	2.4	3.6%	3.63	**D4_1**	3.9	3.3%	3.07
**D4_27**	2.6	3.3%	3.64	**D4_18**	4.1	3.1%	1.12
**D4_23**	2.8	3.1%	3.14	**D4_22**	4.2	3.0%	1.10
**D4_17**	2.8	3.1%	3.37	**D4_24**	4.3	2.7%	3.05
**D4_8**	2.9	2.9%	3.42	**D4_9**	4.4	2.7%	3.15
**D4_3**	3.0	2.8%	3.36	**D4_3**	4.5	2.6%	3.15
**D4_10**	3.1	2.7%	5.24	**D4_27**	5.2	1.9%	3.36
**D4_19**	3.2	2.6%	3.65	**D4_10**	5.2	1.9%	4.88
**D4_18**	3.3	2.5%	1.07	**D4_5**	5.4	1.8%	3.50
**D4_24**	3.6	2.2%	3.36	**D4_12**	5.5	1.7%	3.29
**D4_14**	3.8	2.0%	5.22	**D4_25**	5.6	1.7%	3.61
**D4_9**	3.8	2.0%	3.46	**D4_14**	5.9	1.4%	4.92
**D4_25**	4.2	1.8%	3.84				
**D4_12**	5.1	1.2%	3.87				
**D4_15**	5.6	1.0%	4.11				
			3.13				3.25

5.3.5 Table S16. Relative Gibbs energy and populations of the energetic minima of **D5** (3,3,3-trifluoro-2-methylpropan-1-ol) identified at the SMD/MN15/aug-cc-pVTZ//MN15/cc-pVTZ level of theory in water and octanol medium.

	**Water**				**Octanol**		
Conformer	ΔG kJ mol^{-1}	p_i %	μ D	Conformer	ΔG kJ mol^{-1}	p_i %	μ D
**D5_3**	0.0	16.7%	2.20	**D5_9**	0.0	18.4%	2.82
**D5_6**	0.3	15.0%	2.11	**D5_7**	0.2	16.9%	4.47
**D5_1**	0.8	12.4%	4.62	**D5_8**	0.6	14.7%	2.73
**D5_8**	1.1	10.9%	2.76	**D5_3**	1.3	10.9%	2.07
**D5_5**	1.2	10.2%	4.92	**D5_6**	1.8	8.9%	1.96
**D5_9**	1.3	9.9%	2.95	**D5_1**	2.0	8.1%	4.35
**D5_7**	1.3	9.7%	4.80	**D5_2**	2.2	7.7%	4.48
**D5_4**	1.7	8.5%	2.39	**D5_4**	2.2	7.7%	2.31
**D5_2**	2.3	6.7%	4.71	**D5_5**	2.5	6.7%	4.69
			3.33				3.26

5.3.6 Table S17. Relative Gibbs energy and populations of the energetic minima of **E2** (2-fluoro-2-*c*-propyl-ethanol) identified at the SMD/MN15/aug-cc-pVTZ//MN15/cc-pVTZ level of theory in water and octanol medium.

	**Water**				**Octanol**		
Conformer	ΔG kJ mol^{-1}	p_i %	μ D	Conformer	ΔG kJ mol^{-1}	p_i %	μ D
**E2_3**	0.0	42.4%	4.30	**E2_2**	0.0	52.5%	1.91
**E2_2**	0.2	38.5%	2.06	**E2_3**	1.9	24.4%	4.03
**E2_1**	2.3	16.8%	3.94	**E2_1**	2.5	19.0%	3.63
**E2_4**	8.1	1.6%	2.28	**E2_5**	7.1	1.5%	2.22
**E2_5**	8.5	0.7%	2.25	**E2_4**	7.5	2.6%	2.16
			3.33				2.77

5.3.7 Table S18. Relative Gibbs energy and populations of the energetic minima of **E3** (2-(2'-*syn*-fluoro-*c*-propyl)-ethanol) identified at the SMD/MN15/aug-cc-pVTZ//MN15/cc-pVTZ level of theory in water and octanol medium.

	Water				Octanol		
Conformer	ΔG	p_i	μ	Conformer	ΔG	p_i	μ
	kJ mol^{-1}	%	D		kJ mol^{-1}	%	D
**E3_8**	0.0	25.1%	4.70	**E3_7**	0.0	30.7%	2.95
**E3_7**	0.2	23.6%	3.14	**E3_8**	1.2	18.7%	4.42
**E3_9**	1.2	15.2%	3.20	**E3_9**	1.8	14.6%	2.92
**E3_3**	1.3	15.2%	2.84	**E3_3**	1.9	14.0%	2.70
**E3_2**	1.9	11.7%	3.78	**E3_2**	2.7	10.4%	3.61
**E3_1**	2.9	7.8%	1.10	**E3_1**	3.0	9.3%	0.99
**E3_4**	7.1	1.4%	3.35	**E3_4**	6.5	2.3%	3.09
			3.33				3.07

5.3.8 Table S19. Relative Gibbs energy and populations of the energetic minima of **E4** (2-(2'-*anti*-fluoro-*c*-propyl)-ethanol) identified at the SMD/MN15/aug-cc-pVTZ//MN15/cc-pVTZ level of theory in water and octanol medium.

	Water				Octanol		
Conformer	ΔG	p_i	μ	Conformer	ΔG	p_i	μ
	kJ mol^{-1}	%	D		kJ mol^{-1}	%	D
**E4_7**	0.0	22.0%	2.68	**E4_3**	0.0	19.5%	2.27
**E4_3**	0.4	18.7%	2.41	**E4_7**	0.0	19.1%	2.46
**E4_9**	0.8	15.8%	4.58	**E4_8**	0.4	16.8%	2.89
**E4_2**	1.2	13.8%	1.14	**E4_9**	0.9	13.6%	4.33
**E4_8**	1.3	13.2%	3.06	**E4_2**	1.4	10.9%	1.12
**E4_1**	2.3	8.6%	3.46	**E4_1**	1.7	10.0%	3.29
**E4_6**	4.4	3.7%	3.33	**E4_5**	3.4	5.0%	3.74
**E4_4**	5.7	2.2%	1.40	**E4_6**	4.9	2.7%	3.24
**E4_5**	6.0	1.9%	3.87	**E4_4**	5.2	2.4%	1.46
			2.85				2.75

5.3.9 Table S20. Relative Gibbs energy and populations of the energetic minima of **E5** (2-(2',2'-difluoro-*c*-propyl)-ethanol) identified at the SMD/MN15/aug-cc-pVTZ//MN15/cc-pVTZ level of theory in water and octanol medium.

	Water				Octanol		
Conformer	ΔG kJ mol^{-1}	p_i %	μ D	Conformer	ΔG kJ mol^{-1}	p_i %	μ D
**E5_7**	0.0	26.5%	2.92	**E5_7**	0.0	36.4%	2.74
**E5_9**	0.9	18.4%	4.93	**E5_9**	1.8	17.3%	4.61
**E5_8**	1.0	17.6%	4.92	**E5_8**	2.6	13.0%	4.63
**E5_3**	1.4	15.0%	2.38	**E5_1**	3.1	10.4%	2.53
**E5_1**	2.6	9.3%	2.56	**E5_3**	3.5	9.0%	2.32
**E5_2**	2.6	9.3%	2.86	**E5_2**	3.6	8.6%	2.80
**E5_4**	6.7	1.8%	2.04	**E5_4**	6.9	2.3%	1.92
**E5_6**	7.5	1.3%	4.94	**E5_5**	7.3	1.9%	3.99
**E5_5**	8.3	0.9%	4.45	**E5_6**	8.9	1.0%	4.67
			3.54				3.28

5.3.10 Table S21. Relative Gibbs energy and populations of the energetic minima of **F2** (2-fluoro-2-oxetanyl-ethanol) identified at the SMD/MN15/aug-cc-pVTZ//MN15/cc-pVTZ level of theory in water and octanol medium.

	Water				Octanol		
Conformer	ΔG kJ mol^{-1}	p_i %	μ D	Conformer	ΔG kJ mol^{-1}	p_i %	μ D
**F2_1**	0.0	40.9%	2.15	**F2_1**	0.0	53.3%	1.95
**F2_2**	1.6	21.8%	1.85	**F2_2**	2.8	17.0%	1.74
**F2_3**	1.2	25.3%	3.41	**F2_3**	2.3	21.0%	3.18
**F2_5**	4.3	3.6%	3.85	**F2_5**	4.9	3.7%	3.65
**F2_8**	3.9	8.3%	2.40	**F2_8**	5.9	5.0%	2.18
			2.48				2.25

5.3.11 Table S22. Relative Gibbs energy and populations of the energetic minima of **G2** (2-fluoro-butan-1-ol) identified at the SMD/MN15/aug-cc-pVTZ//MN15/cc-pVTZ level of theory in water and octanol medium.

	**Water**				**Octanol**		
Conformer	Δ*G*	*p*	μ	Conformer	Δ*G*	*p*	μ
	kJ mol^{-1}	%	D		kJ mol^{-1}	%	D
**G2_5**	0.0	15.0%	2.60	**G2_5**	0.0	24.6%	2.43
**G2_26**	1.0	10.3%	2.13	**G2_26**	2.3	9.8%	1.91
**G2_23**	1.3	9.0%	4.53	**G2_4**	2.5	9.0%	2.36
**G2_27**	1.6	7.8%	1.97	**G2_6**	2.6	8.7%	2.57
**G2_6**	1.7	7.6%	2.71	**G2_27**	3.1	7.1%	1.77
**G2_14**	1.7	7.6%	4.28	**G2_23**	3.2	6.7%	4.23
**G2_15**	2.3	6.1%	4.33	**G2_14**	3.3	6.5%	3.97
**G2_4**	2.3	6.1%	2.52	**G2_24**	3.6	5.7%	4.36
**G2_24**	2.6	5.3%	4.62	**G2_17**	4.4	4.2%	3.76
**G2_22**	2.7	5.0%	4.52	**G2_22**	4.6	3.8%	4.22
**G2_17**	3.3	3.9%	4.12	**G2_13**	5.6	2.5%	3.82
**G2_8**	3.5	3.7%	4.58	**G2_8**	5.7	2.4%	4.28
**G2_13**	3.7	3.4%	4.14	**G2_18**	6.0	2.2%	3.75
**G2_18**	4.0	3.0%	4.05	**G2_11**	6.1	2.1%	2.24
**G2_9**	4.6	2.3%	4.66	**G2_20**	6.2	2.0%	2.57
**G2_20**	5.4	1.7%	2.71	**G2_2**	6.5	1.8%	1.94
**G2_11**	6.4	1.1%	2.31	**G2_9**	7.9	1.0%	4.39
				**G2_5**	0.0	24.6%	2.43
			3.38				2.91

5.3.12 Table S23. Relative Gibbs energy and populations of the energetic minima of **G3** (3-fluoro-butan-1-ol) identified at the SMD/MN15/aug-cc-pVTZ//MN15/cc-pVTZ level of theory in water and octanol medium.

	Water				Octanol		
Conformer	ΔG	p_i	μ	Conformer	ΔG	p_i	μ
	kJ mol^{-1}	%	D		kJ mol^{-1}	%	D
**G3_8**	0.0	11.3%	4.46	**G3_11**	0.0	15.9%	1.87
**G3_20**	0.0	11.2%	1.96	**G3_2**	0.6	12.6%	4.17
**G3_2**	0.4	9.6%	4.47	**G3_8**	1.2	9.8%	4.17
**G3_11**	0.4	9.5%	2.02	**G3_14**	1.5	8.7%	1.95
**G3_5**	0.5	9.4%	2.33	**G3_20**	1.5	8.6%	1.78
**G3_14**	1.1	7.4%	2.14	**G3_5**	1.7	8.0%	2.13
**G3_23**	1.3	6.8%	4.48	**G3_23**	2.7	5.4%	4.21
**G3_18**	2.6	3.9%	4.37	**G3_18**	3.5	3.9%	4.16
**G3_27**	2.8	3.7%	1.97	**G3_27**	3.6	3.7%	2.01
**G3_6**	2.8	3.7%	2.22	**G3_15**	4.2	2.9%	4.26
**G3_24**	3.3	3.0%	2.55	**G3_24**	4.4	2.7%	2.44
**G3_26**	3.5	2.8%	4.11	**G3_6**	4.6	2.4%	2.10
**G3_15**	3.8	2.4%	4.51	**G3_12**	5.2	2.0%	4.10
**G3_4**	4.6	1.8%	4.60	**G3_3**	5.3	1.9%	1.92
**G3_3**	4.6	1.7%	1.85	**G3_17**	5.4	1.8%	4.30
**G3_17**	4.7	1.7%	4.68	**G3_4**	5.5	1.8%	4.33
**G3_9**	4.8	1.7%	2.81	**G3_22**	5.7	1.6%	1.99
**G3_13**	4.9	1.6%	2.98	**G3_13**	5.7	1.6%	2.76
**G3_22**	4.9	1.6%	2.20	**G3_16**	5.8	1.5%	1.57
**G3_12**	5.1	1.5%	4.31	**G3_26**	6.3	1.2%	3.75
**G3_19**	5.2	1.4%	4.48	**G3_19**	6.5	1.2%	4.21
**G3_25**	5.7	1.1%	3.92	**G3_9**	6.8	1.0%	2.67
**G3_7**	5.7	1.1%	2.24				
			3.18				2.92

6 Synthesis

6.1 General methods.

All chemical reagents were obtained from commercial sources and used without further purification. Anhydrous solvents were purchased from commercial sources. All glassware was flame-dried under vacuum and cooled under Ar prior to use. Water or air sensitive reactions were performed under inert atmosphere, using dry solvents. Reactions were monitored by TLC (Merck Kieselgel 60 F_{254}, aluminium sheet) and spots were visualized by UV and/or by exposure to a basic solution of $KMnO_4$, followed by brief heating. Flash column chromatography was performed on silica gel (Merck silica gel 60, particle size 40–63 µm). All reported solvent mixtures are volume measures. Nuclear magnetic resonance spectra were recorded using either a Bruker Ultrashield 400 MHz or 500 MHz spectrometer. The chemical shift (δ) is given in ppm using the residual solvent peak as an internal standard. The coupling constants (*J*) are given in Hertz (Hz). IR spectra were recorded on a Thermo Scientific™ Nicolet iS5 as films and absorption peaks are given in cm^{-1}. Low resolution electrospray mass spectra were recorded with a Waters Acquity TQD mass tandem quadrupole mass spectrometer. HRMS spectra were measured on a Bruker Daltonics MaXis time of flight (TOF) mass spectrometer or, for volatile compounds, a Thermo MAT900 XP double focusing sector mass spectrometer. All compounds subjected to biological assays were of >95% purity (liquid chromatography–UV).

6.2 Compounds available

Compounds **D1–D3**, **D5**, **F2** were commercially available.

6.3 Synthesis of novel compounds

6.3.1 Synthesis of 3,3-difluoro-2-methylpropan-1-ol (D4)

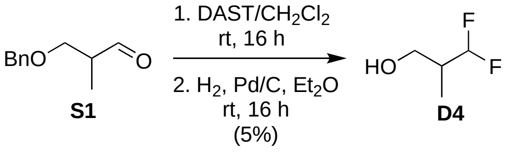

To a 250 mL two-neck round-bottom flask were added 3-benzyloxy-2-methylpropanal **S1**[8] (2.37 g, 13.3 mmol, 1.0 equiv) and anhydrous CH_2Cl_2 (80 mL). The resulting mixture was cooled to 0 °C, followed by slow addition of (diethylamino)sulfur trifluoride (5.27 mL, 39.9 mmol, 3.0 equiv) at this temperature. The reaction mixture was warmed to room temperature and stirred for 16 h. Upon completion as indicated by TLC analysis, the reaction mixture was slowly quenched with sat. aq. $NaHCO_3$ solution (150 mL), followed by extraction with CH_2Cl_2 (100 mL × 3). The combined organic layer was dried over Na_2SO_4, filtered and concentrated. The crude mixture was purified by flash chromatography (eluent: CH_2Cl_2/petroleum ether 20/80 – 25/75) to obtain the desired difluorinated

intermediate (not pure). This intermediate was treated with Pd/C (10wt% loading, 700 mg) in Et_2O (40 mL) under argon. The resulting mixture was degassed with H_2 gas and then stirred under H_2 at room temperature overnight. Upon completion was indicated by TLC analysis, the reaction mixture was loaded directly into a column for purification (eluent: pentane 100% – CH_2Cl_2/pentane 50/50 – Et_2O/CH_2Cl_2 10/90) to afford the desired product in solution. This solution was concentrated at 700 mbar/35 °C and further dried by slow evaporation at atmospheric pressure to obtain the desired product **D4** (72 mg, 0.65 mmol, 5%) as a colourless oil. The low yield was due to the volatility of the product. **^{1}H NMR** (400 MHz, $CDCl_3$) δ 5.86 (1H, td, *J* 56.7, 3.9 Hz, H-3), 3.63 – 3.76 (2H, m, H-1), 2.05 – 2.25 (1H, m, H-2), 1.64 (1H, t, *J* 4.2 Hz, O*H*), 1.05 (3H, d, *J* 7.1 Hz, C*H*$_3$) ppm; **^{13}C NMR** (101 MHz, $CDCl_3$) δ 118.0 (t, *J* 242.1 Hz, C-3), 62.6 (dd, *J* 6.2, 4.8 Hz, C-1), 39.8 (t, *J* 19.3 Hz, C-2), 9.3 (t, *J* 5.3 Hz, *C*H$_3$) ppm; **^{19}F NMR** (376 MHz, $CDCl_3$) δ -124.0 (1F, ddd, *J* 281.8, 56.8, 12.1 Hz, F-3), -126.8 (1F, ddd, *J* 282.6, 56.9, 18.2 Hz, F-3') ppm; **^{19}F{^{1}H} NMR** (376 MHz, $CDCl_3$) δ -124.0 (1F, d, *J* 281.8 Hz, F-3), -126.8 (1F, d, *J* 282.6 Hz, F-3') ppm; **IR** (neat) 3356 (br. m), 2982 (m), 2957 (w), 2896 (w), 1467 (m), 1399 (m), 1154 (m), 1086 (s), 1025 (s), 988 (s), 942 (m) cm^{-1}.

6.3.2 Synthesis of (1-fluorocyclopropyl)methanol (**E2**)

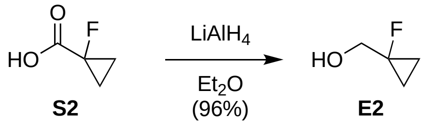

A solution of **S2** (250 mg, 1 equiv) in Et_2O (2 mL) was added to a slurry of $LiAlH_4$ (273 mg, 3 equiv) in Et_2O (6 mL) at 0 °C dropwise. The reaction was allowed to warm to room temperature and stirred for 16 h. It was then cooled to 0 °C and water (0.27 mL) was added dropwise, followed by aq. NaOH (15% wt., 0.27 mL) then water (0.81 mL). To this $MgSO_4$ was added and after stirring for 15 min the mixture was filtered and the resultant filtrate was carefully concentrated at 750 mbar/30 °C to afford **E2** as a colourless oil (208 mg, 96%). **^{1}H NMR** (400 MHz, $CDCl_3$) δ 3.84 (d, *J*=22.0 Hz, 2H, H1), 1.88 (br. s, 1H, OH), 1.17–1.04 (m, 2H, H3' + H4'), 0.75–0.67 (m, 2H, H3'' + H4'') ppm; **^{13}C NMR** (101 MHz, $CDCl_3$) δ 79.9 (d, *J*=216.4 Hz, C2), 66.1 (d, *J*=22.0 Hz, C1), 9.2 (d, *J*=11.7 Hz, C3 and C4 overlapped) ppm; **^{19}F NMR** (376 MHz, $CDCl_3$) δ -191.3 (ttt, *J*=22.0, 18.6, 8.7 Hz, 1F) ppm; **^{19}F {^{1}H} NMR** (376 MHz, $CDCl_3$) δ -191.3 (s, 1F) ppm; **IR** (neat) 3339 (br. w), 2931 (w), 2872 (w), 1416 (m), 1200 (m), 1038 (s), 1013 (s) cm^{-1}; **HRMS** (CI) for C_4H_8FO $(M+H)^+$, calculated 91.0554, found 91.0544. Data consistent with literature.[9]

6.3.3 Synthesis of (*rac*-(1*R*,2*R*)-2-fluorocyclopropyl)methanol (**E3**)

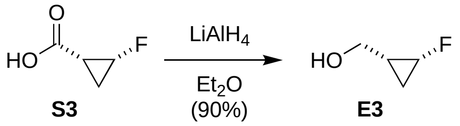

A solution of **S3** (250 mg, 1 equiv) in Et_2O (2 mL) was added to a slurry of $LiAlH_4$ (273 mg, 3 equiv) in Et_2O (6 mL) at 0 °C dropwise. The reaction was allowed to warm to room temperature and stirred for 16 h. It was then cooled to 0 °C and water (0.27 mL) was added dropwise, followed by aq. NaOH (15% wt., 0.27 mL) then water (0.81 mL). To this $MgSO_4$ was added and after stirring for 15 min the mixture was filtered and the resultant filtrate was carefully concentrated at 750 mbar/30 °C to afford **E3** as a colourless oil (195 mg, 90%). **^{1}H NMR** (500 MHz, $CDCl_3$) δ 4.74 (dtd, *J*=65.2, 5.9, 2.7 Hz, 1H, H4), 3.94 (br. d, *J*=4.3 Hz, 1H, H1'), 3.69–3.60 (m, 1H, H1''), 1.52 (br. s, 1H, OH), 1.34–1.15 (m, 1H, H2), 0.92–0.71 (m, 2H, H3) ppm; **^{13}C NMR** (126 MHz, $CDCl_3$) δ 72.3 (d, *J*=218.8 Hz, C4), 61.1 (d, *J*=9.2 Hz, C1), 18.7 (d, *J*=11.0 Hz, C2), 9.4 (d, *J*=10.1 Hz, C3) ppm; **^{19}F NMR** (471 MHz, $CDCl_3$) δ -227.4 (dddd, *J*=65.0, 23.4, 11.3, 5.2 Hz, 1F) ppm; **^{19}F {^{1}H} NMR** (471 MHz, $CDCl_3$) δ -227.4 (s, 1 F) ppm; **IR** (neat) 3334 (br. w), 2943 (w), 2888 (w), 1445 (m), 1200 (s), 1016 (s), 982 (s) cm^{-1}; **HRMS** (CI) for C_4H_8FO $(M+H)^+$, calculated 91.0554, found 91.0544.

6.3.4 Synthesis of (*rac*-(1*R*,2S)-2-fluorocyclopropyl)methanol (**E4**)

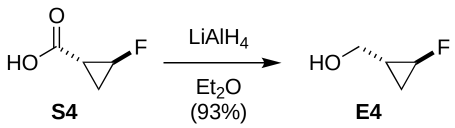

A solution of **S4** (250 mg, 1 equiv) in Et_2O (2 mL) was added to a slurry of $LiAlH_4$ (273 mg, 3 equiv) in Et_2O (6 mL) at 0 °C dropwise. The reaction was allowed to warm to room temperature and stirred for 16 h. It was then cooled to 0 °C and water (0.27 mL) was added dropwise, followed by aq. NaOH (15% wt., 0.27 mL) then water (0.81 mL). To this $MgSO_4$ was added and after stirring for 15 min the mixture was filtered and the resultant filtrate was carefully concentrated at 750 mbar/30 °C to afford **E4** as a colourless oil (201 mg, 93%). **^{1}H NMR** (400 MHz, $CDCl_3$) δ 4.47 (ddt, *J*=64.1, 6.2, 2.3 Hz, 1H, H4), 3.55–3.42 (m, 2H, H1), 1.63–1.51 (m, 1H, H2), 1.48 (br. t, *J*=4.5 Hz, 1H, OH), 1.15–1.04 (m, 1H, H3'), 0.62 (app. dq, *J*=10.3, 6.7 Hz, 1H, H3') ppm; **^{13}C NMR** (101 MHz, $CDCl_3$) δ 72.5 (d, *J*=220.6 Hz, C4), 62.7 (C1), 20.2 (d, *J*=10.1 Hz, C2), 9.7 (d, *J*=11.0 Hz, C3) ppm; **^{19}F NMR** (471 MHz, $CDCl_3$) δ -210.2 (dtdd, *J*=64.2, 20.8, 10.4, 1.7 Hz, 1F) ppm; **^{19}F {^{1}H} NMR** (471 MHz, $CDCl_3$) δ -210.2 (s, 1F) ppm; **IR** (neat) 3335 (br. w), 2930 (w), 2881 (w), 1453 (m), 1135 (s), 1031 (s), 982 (s) cm^{-1}; **HRMS** (CI) for C_4H_8FO $(M+H)^+$, calculated 91.0554, found 91.0545.

6.3.5 Synthesis of (2,2-difluorocyclopropyl)methanol (**E5**)

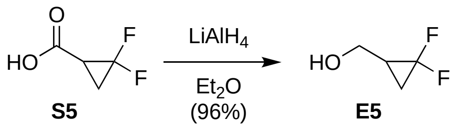

A solution of **S5** (250 mg, 1 equiv) in Et_2O (2 mL) was added to a slurry of $LiAlH_4$ (233 mg, 3 equiv) in Et_2O (6 mL) at 0 °C dropwise. The reaction was allowed to warm to room temperature and stirred

for 16 h. It was then cooled to 0 °C and water (0.23 mL) was added dropwise followed by aq. NaOH (15% wt., 0.23 mL) then water (0.69 mL). To this $MgSO_4$ was added and after stirring for 15 min the mixture was filtered and the resultant filtrate was carefully concentrated at 750 mbar/30 °C to afford **E5** as a colourless oil (214 mg, 97%). **^{1}H NMR** (400 MHz, $CDCl_3$) δ 3.98–3.54 (m, 2H, H1), 1.99–1.83 (m, 1H, H2), 1.48 (tdd, *J*=11.9, 7.6, 4.2 Hz, 1H, H3'), 1.18 (dtd, *J*=13.3, 7.6, 3.8 Hz, 1H, H3'') ppm; **^{13}C NMR** (101 MHz, $CDCl_3$) δ 113.6 (t, *J*=281.7 Hz, C4), 60.1 (d, *J*=5.9 Hz, C1), 24.3 (t, *J*=10.6 Hz, C2), 14.5 (t, *J*=11.4 Hz, C3) ppm; **^{19}F NMR** (376 MHz, $CDCl_3$) δ -128.8 (app. dtt, *J*=161.3, 12.1, 3.5 Hz, 1F, F4'), -144.5 (app. ddd, *J*=160.8, 13.4, 4.3 Hz, 1F, F4'') ppm; **^{19}F {^{1}H} NMR** (376 MHz, $CDCl_3$) δ -128.8 (d, *J*=161.3 Hz, 1F, F4''), -144.5 (d, *J*=161.3 Hz, 1F, F4'') ppm. Data consistent with literature.[10]

7 NMR spectra for novel compounds

7.1 3,3-Difluoro-2-methylpropan-1-ol (D4)

7.1.1 3,3-Difluoro-2-methylpropan-1-ol (**D4**) (^{1}H NMR, $CDCl_3$, 400 MHz)

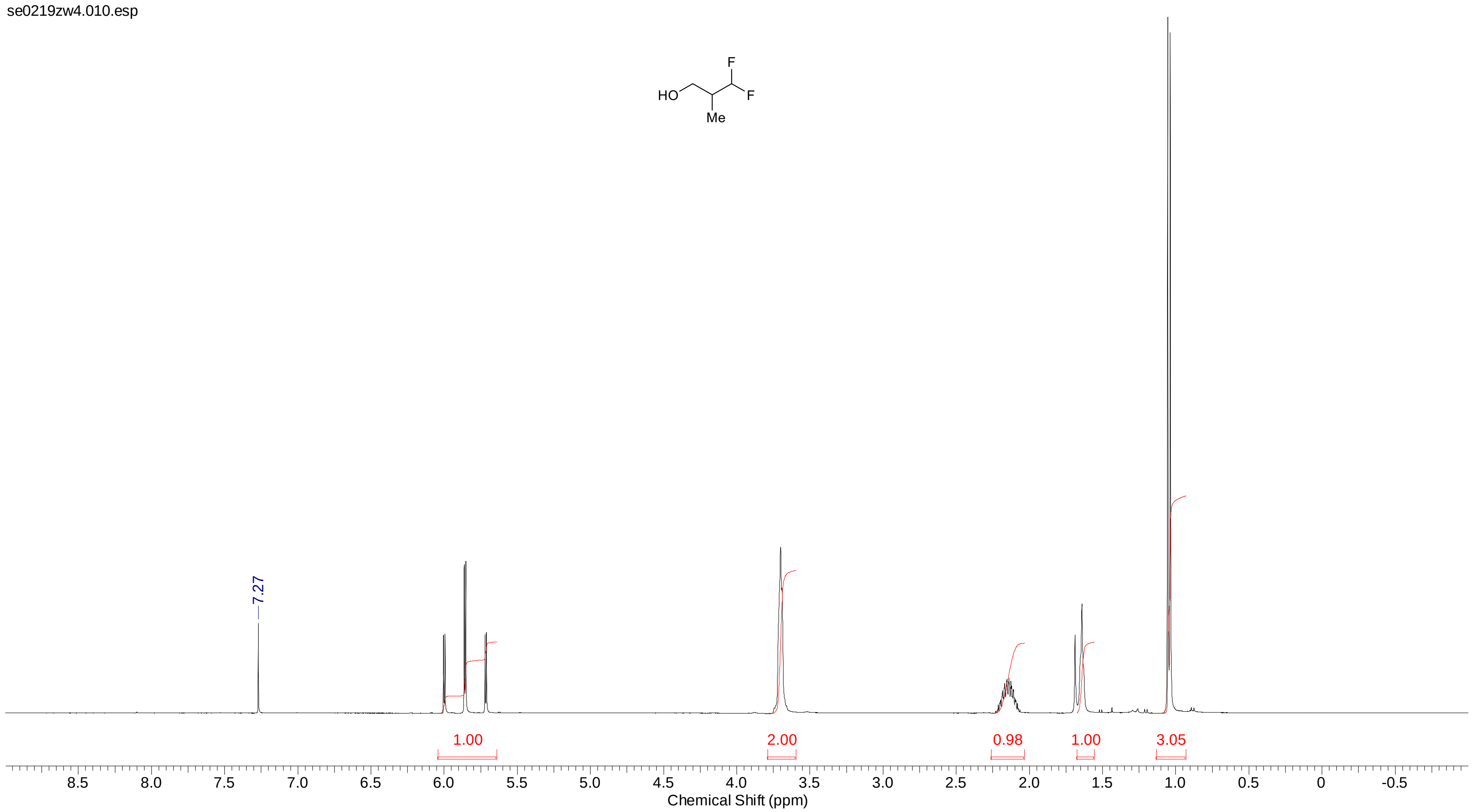

7.1.2 3,3-Difluoro-2-methylpropan-1-ol (**D4**) (^{19}F NMR, $CDCl_3$, 376 MHz)

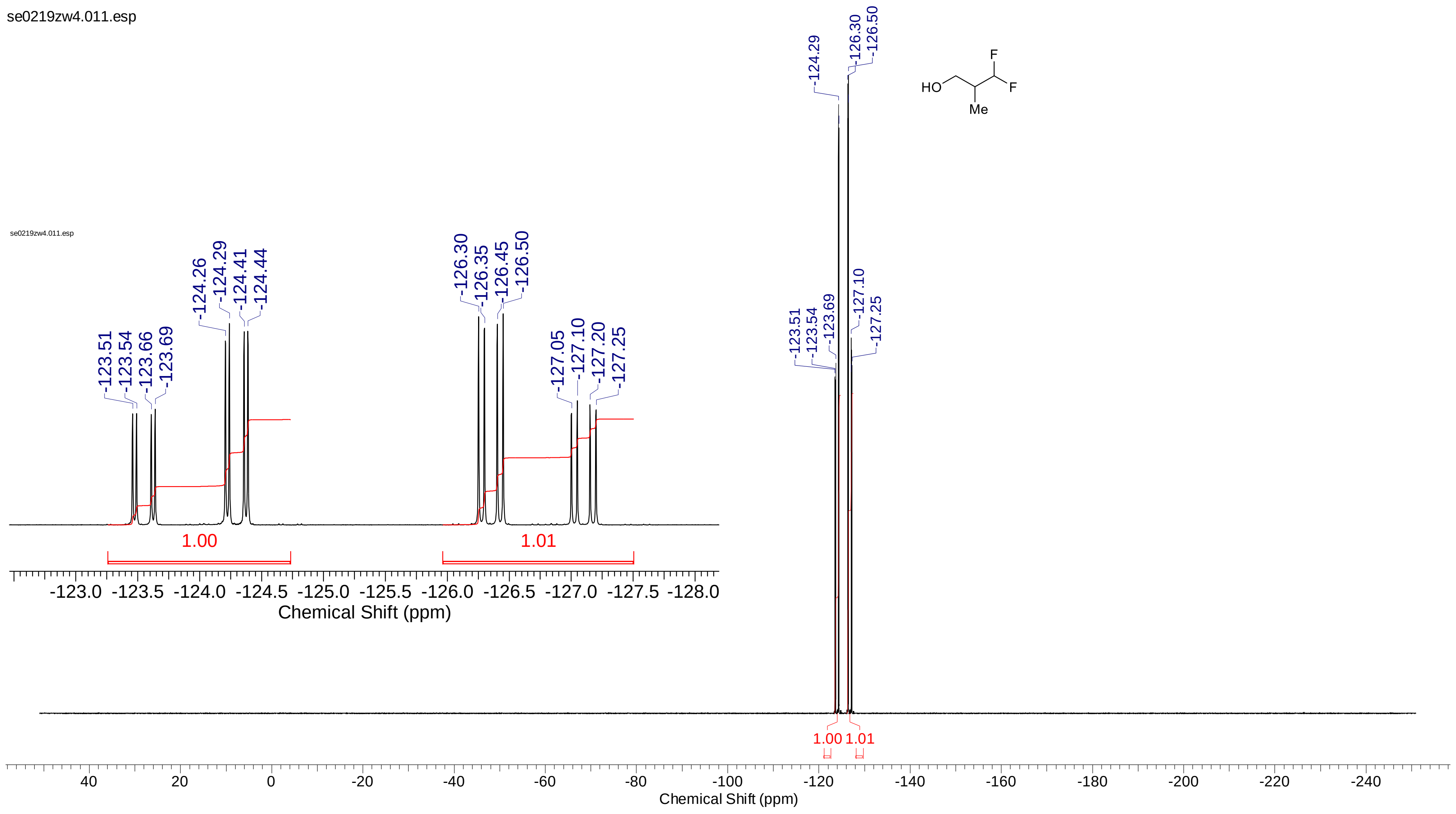

7.1.3 3,3-Difluoro-2-methylpropan-1-ol (**D4**) ($^{19}F\{^1H\}$ NMR, $CDCl_3$, 376 MHz)

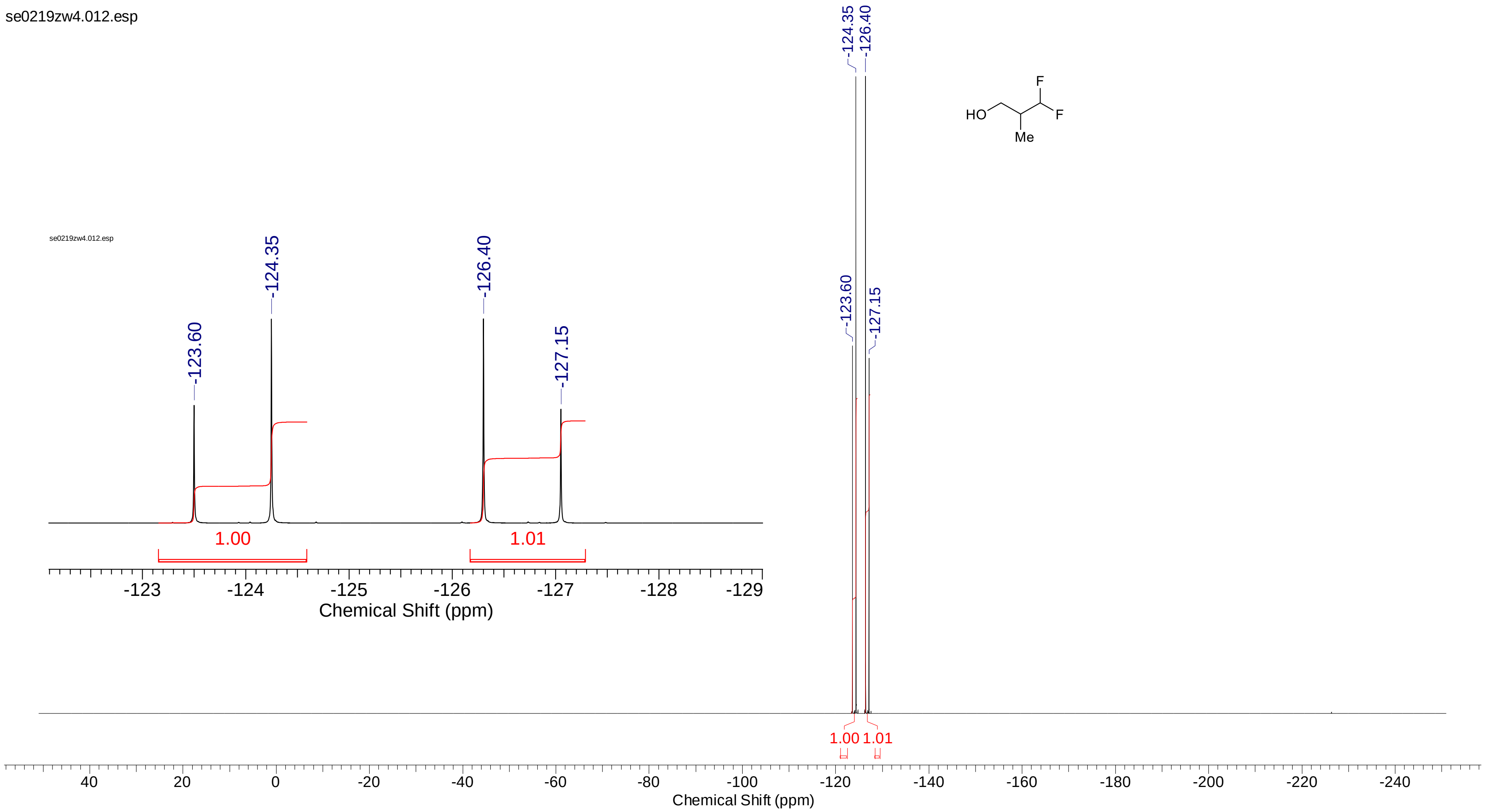

7.1.4 3,3-Difluoro-2-methylpropan-1-ol (**D4**) (^{13}C NMR, $CDCl_3$, 101 MHz)

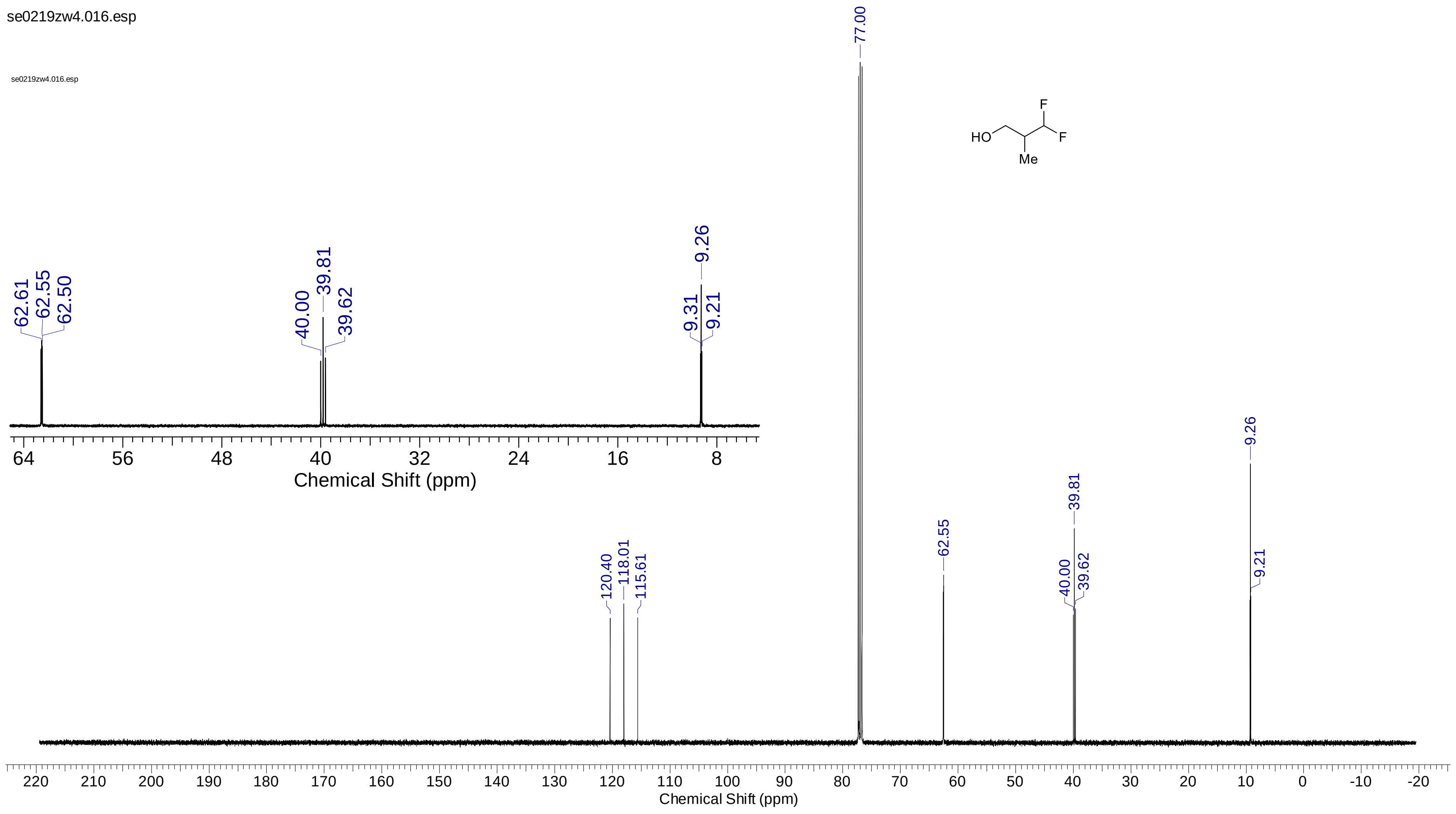

7.2 (1-Fluorocyclopropyl)methanol (E2)

7.2.1 (1-Fluorocyclopropyl)methanol (**E2**) (^{1}H NMR, $CDCl_3$, 400 MHz)

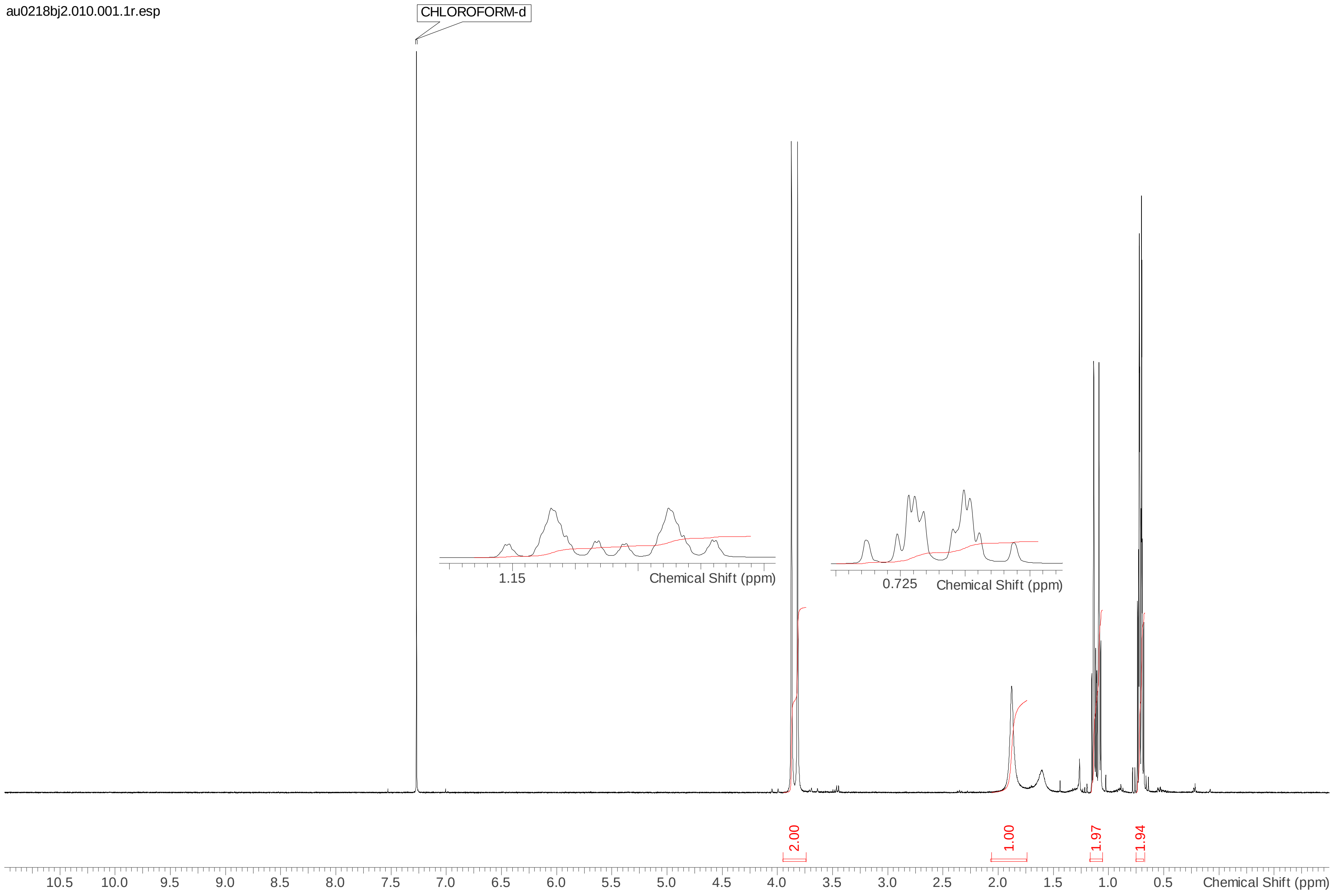

7.2.2 (1-Fluorocyclopropyl)methanol (**E2**) (^{13}C NMR, $CDCl_3$, 101 MHz)

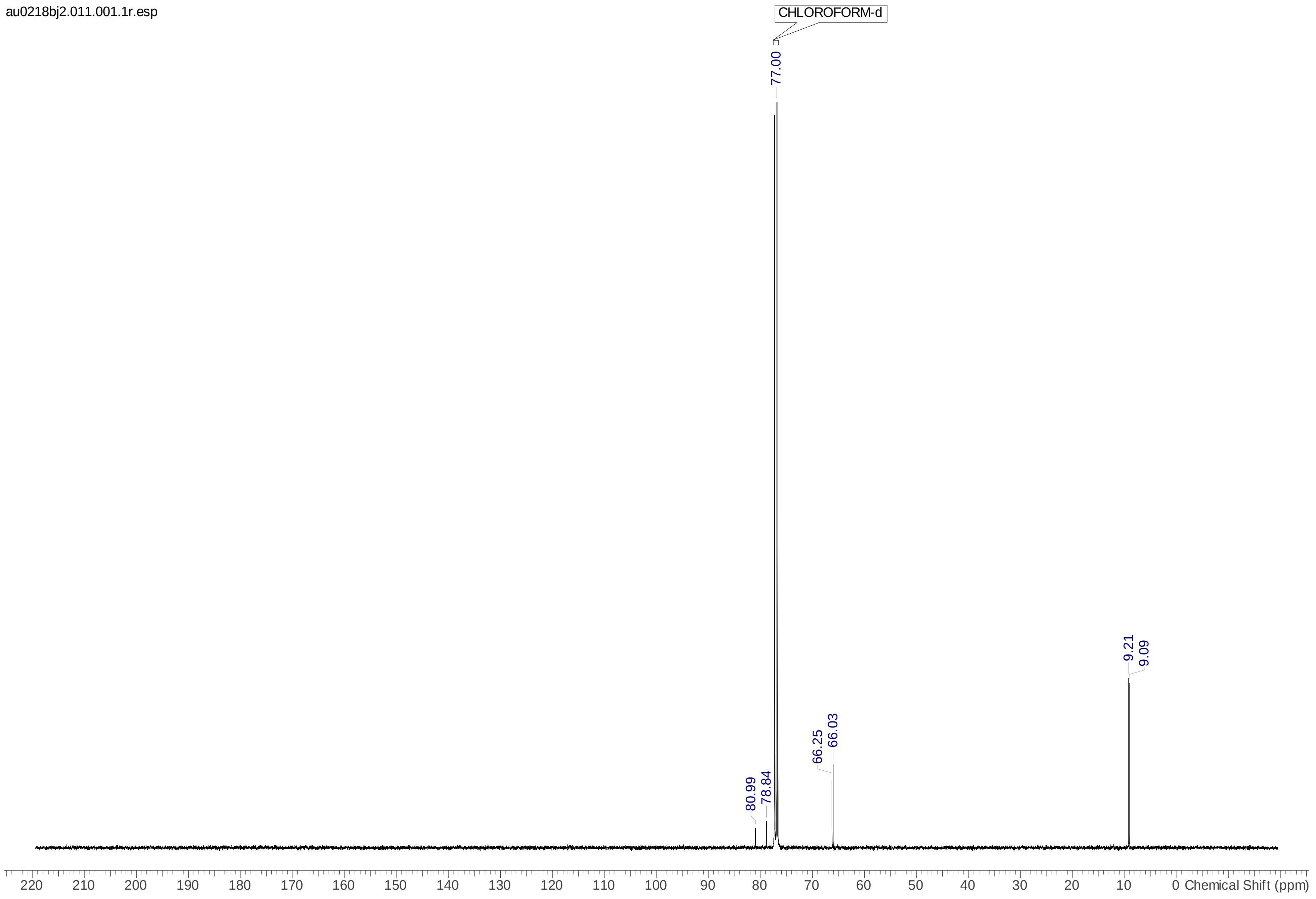

7.2.3 (1-Fluorocyclopropyl)methanol (**E2**) (^{19}F NMR, $CDCl_3$, 376 MHz)

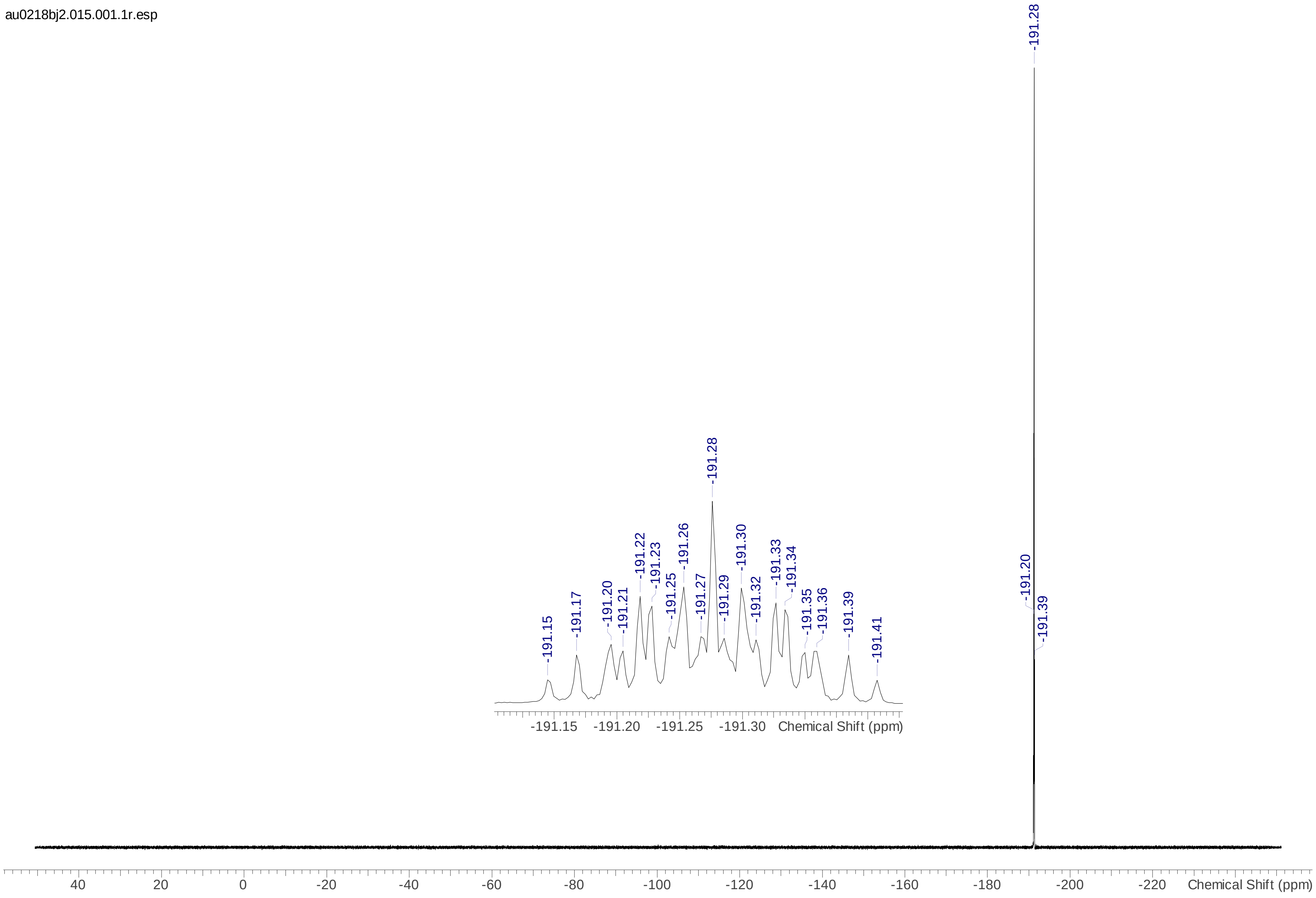

7.2.4 (1-Fluorocyclopropyl)methanol (**E2**) (^{19}F {1H} NMR, $CDCl_3$, 376 MHz)

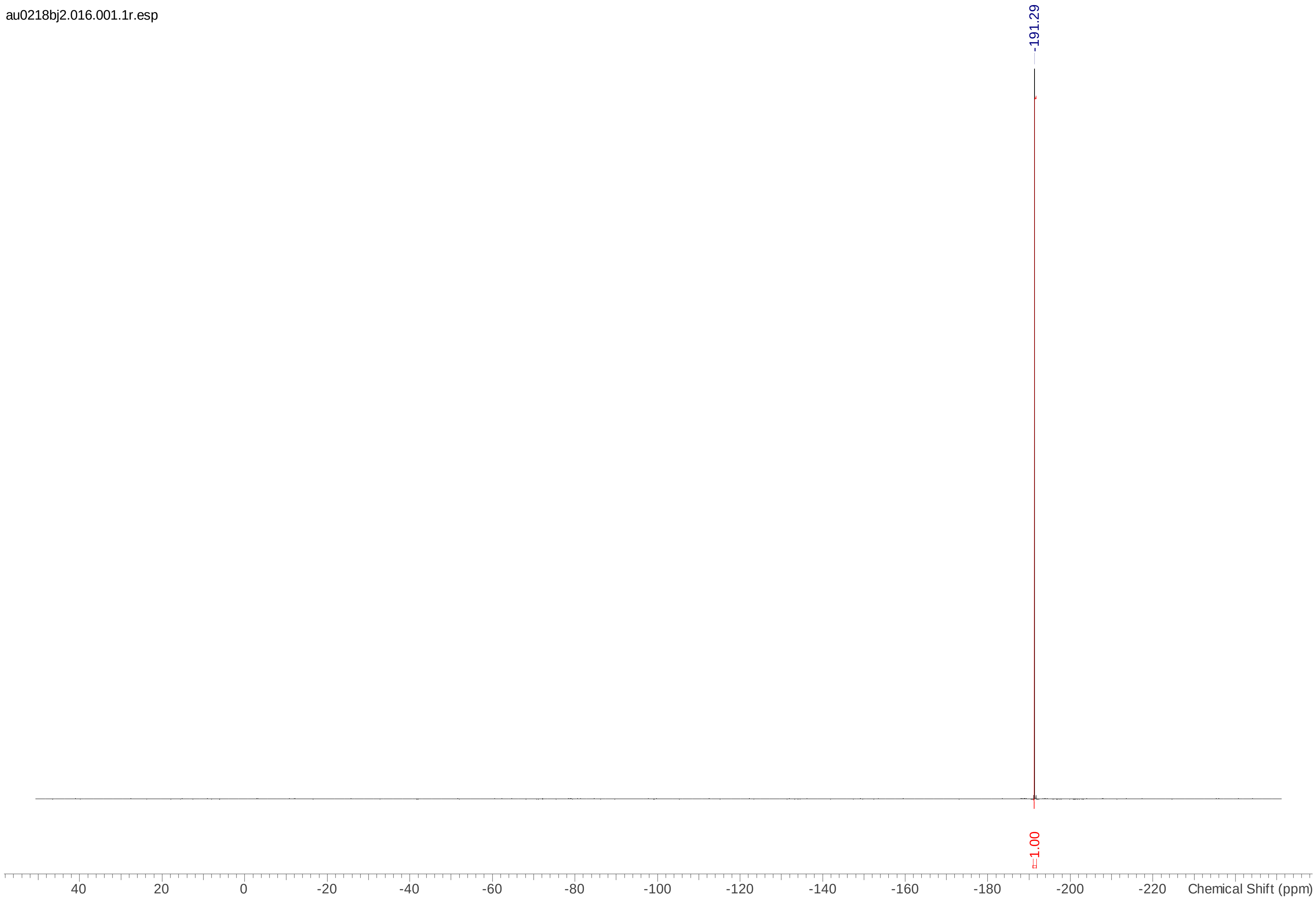

7.3 *rac*-(1*R*,2*R*)-(2-Fluorocyclopropyl)methanol (E3)

7.3.1 *rac*-(1*R*,2*R*)-2-Fluorocyclopropyl)methanol (**E3**) (^{1}H NMR, $CDCl_3$, 400 MHz)

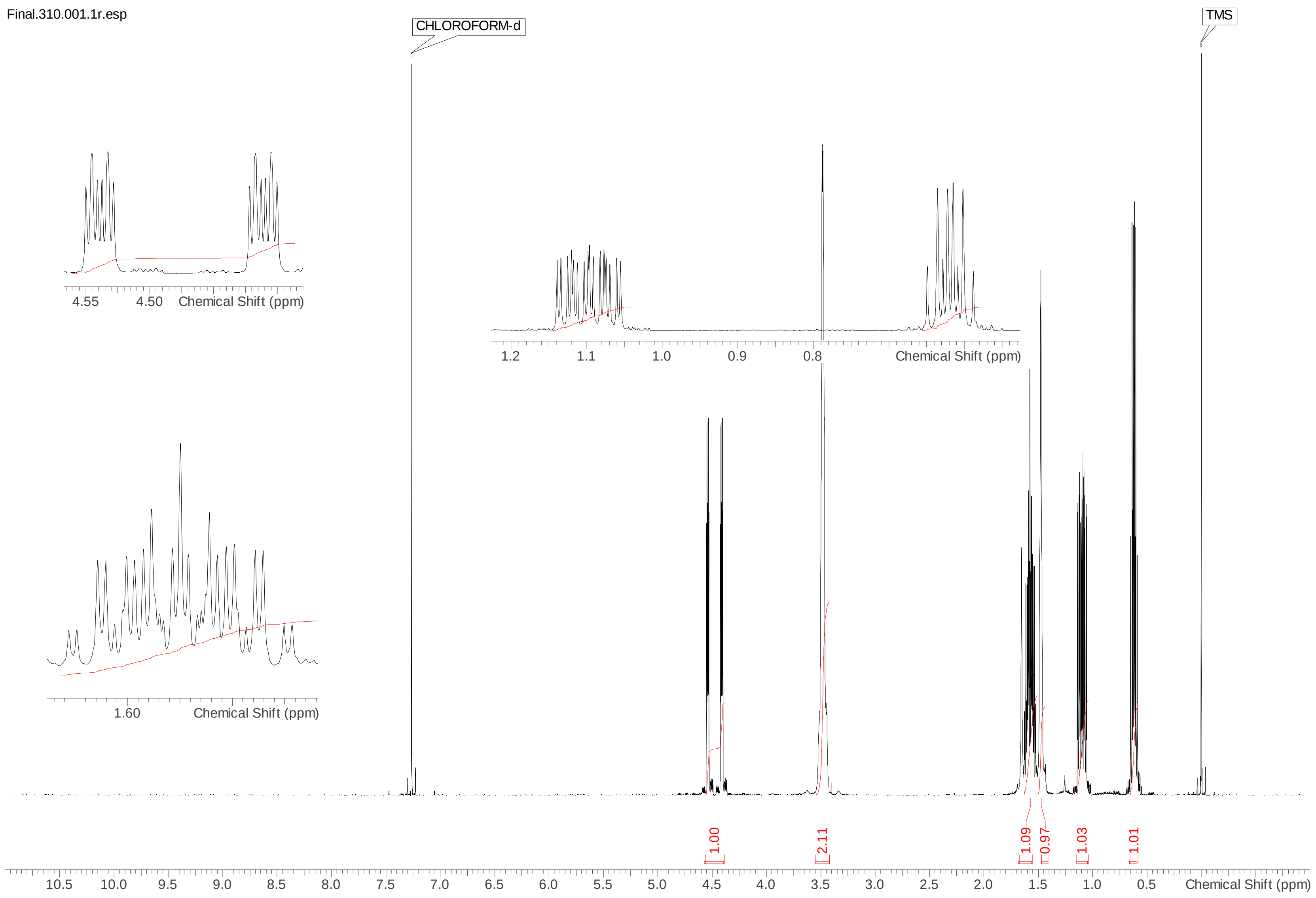

7.3.2 *rac*-(1*R*,2*R*)-(2-Fluorocyclopropyl)methanol (**E3**) (^{13}C NMR, $CDCl_3$, 126 MHz)

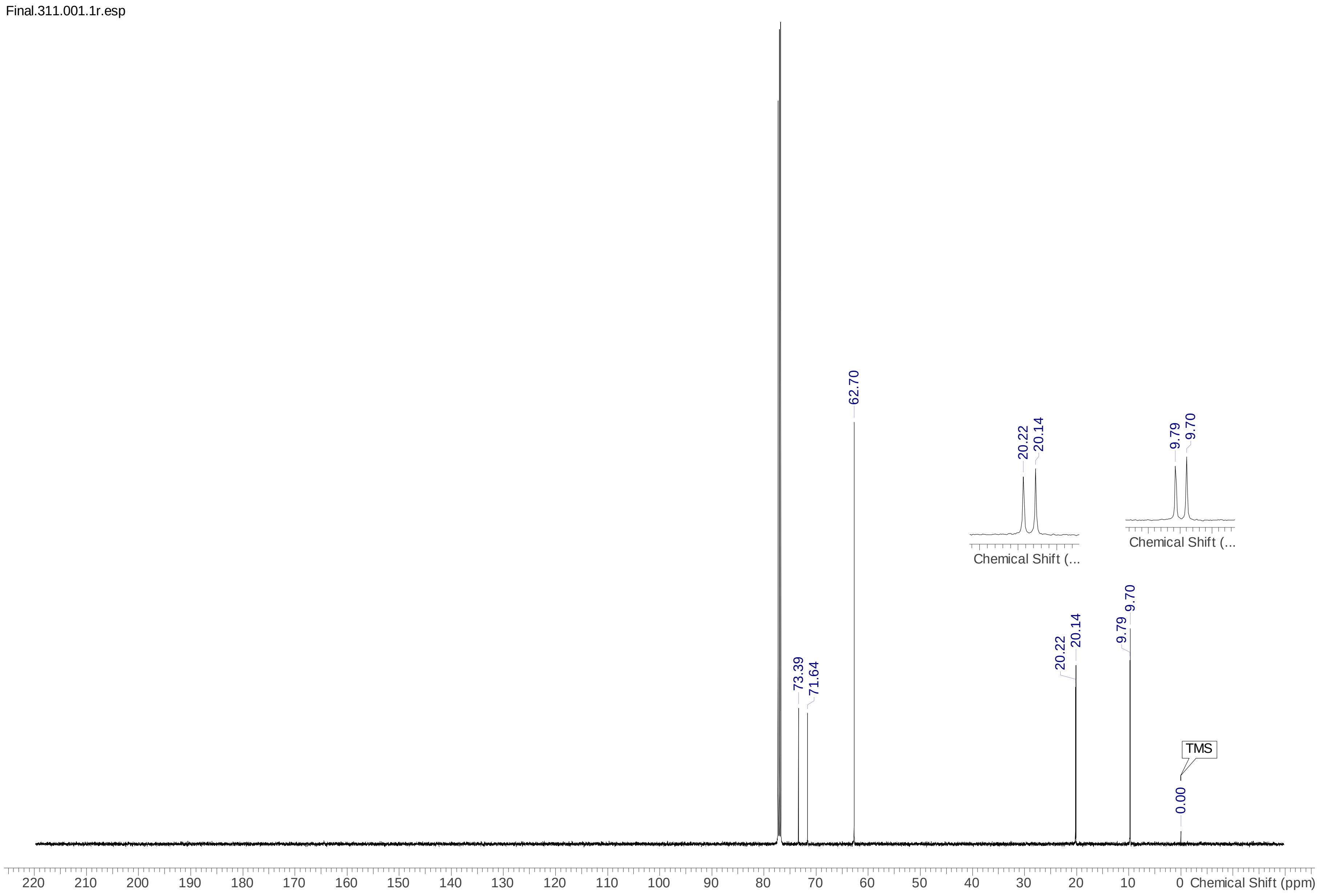

7.3.3 *rac*-(1*R*,2*R*)-(2-Fluorocyclopropyl)methanol (**E3**) (^{19}F NMR, $CDCl_3$, 471 MHz)

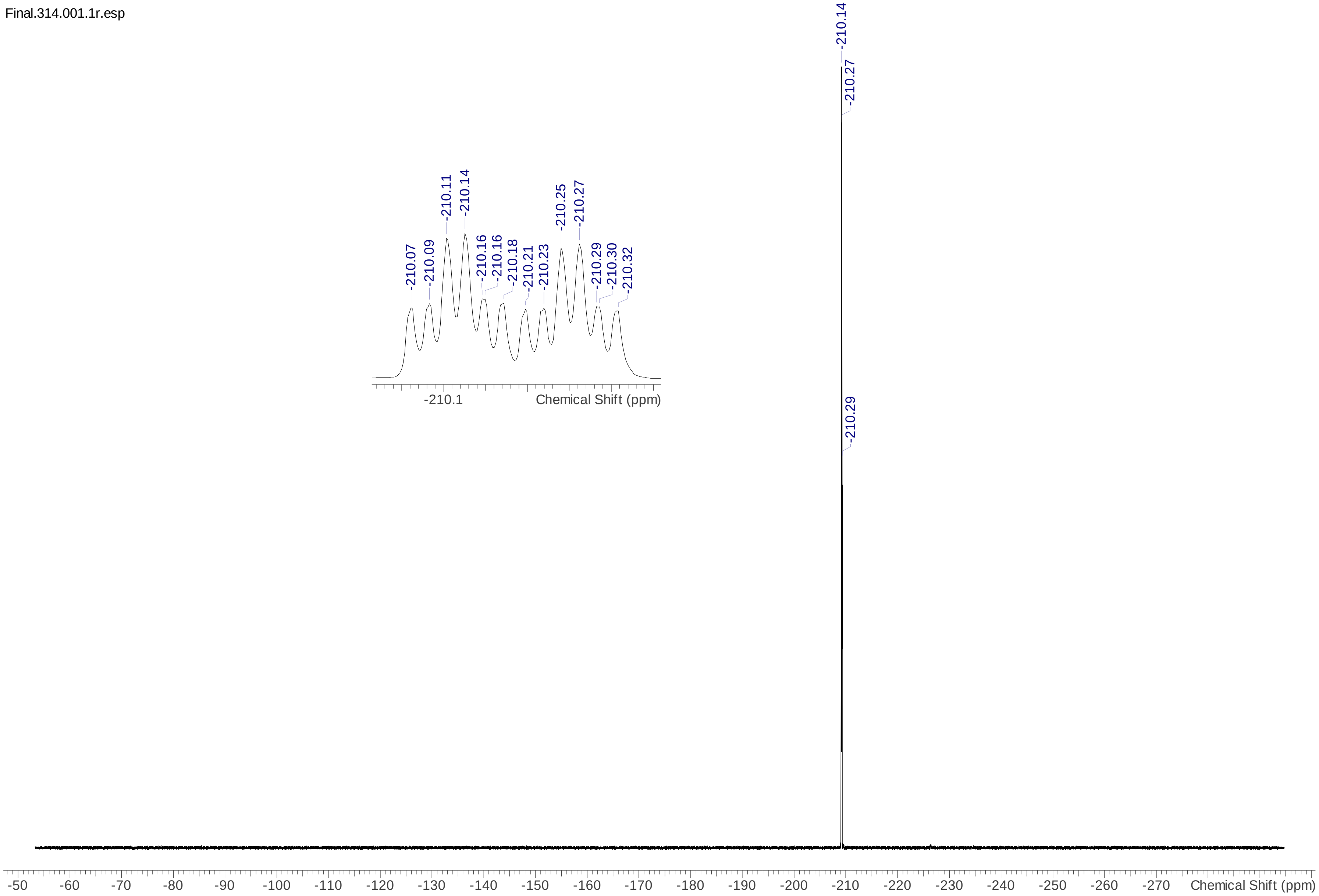

7.3.4 *rac*-(1*R*,2*R*)-(2-Fluorocyclopropyl)methanol (**E3**) (^{19}F {1H} NMR, $CDCl_3$, 471 MHz)

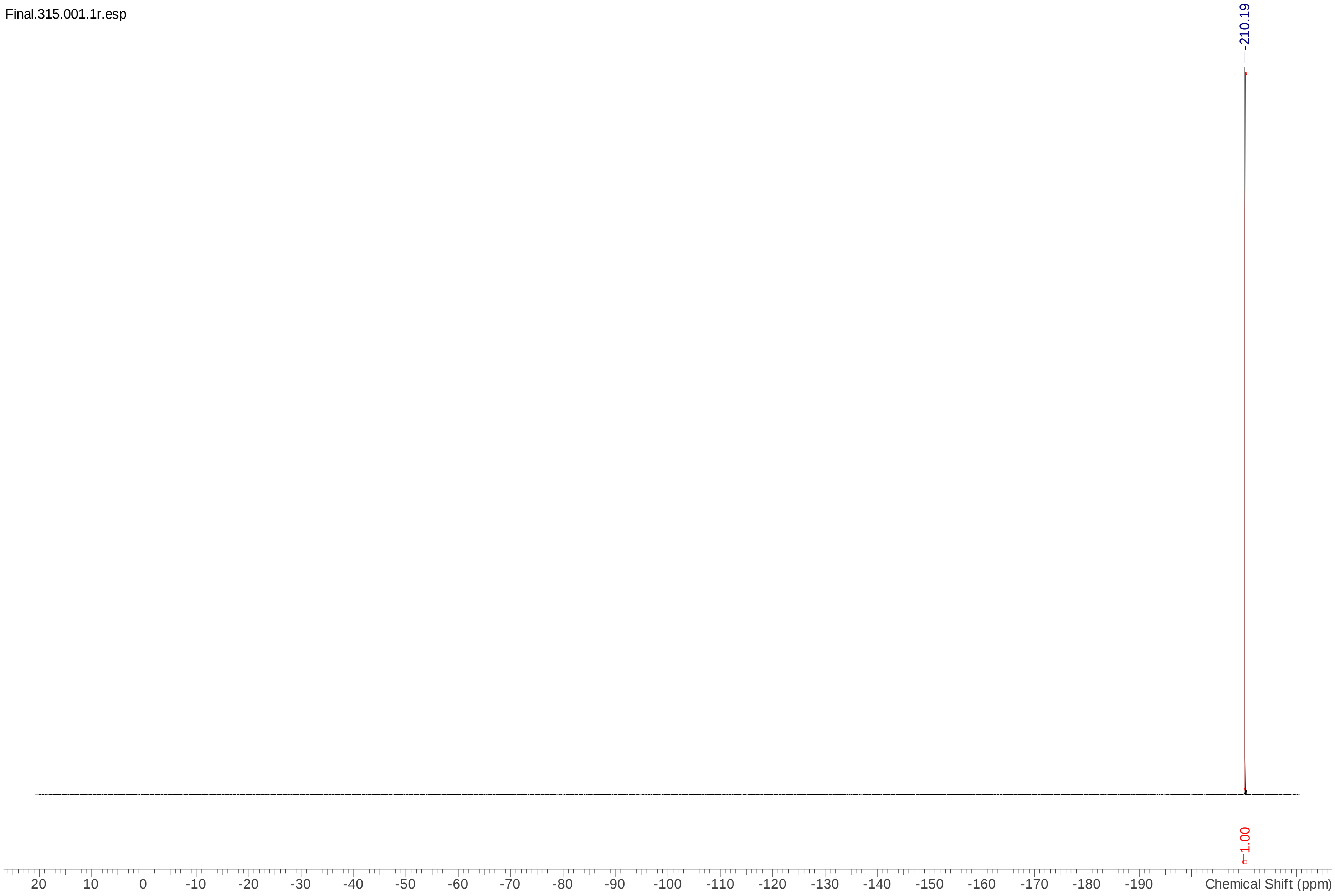

7.4 *rac*-(1*R*,2*S*)-(2-fluorocyclopropyl)methanol (E4)

7.4.1 *rac*-(1*R*,2*S*)-(2-fluorocyclopropyl)methanol (**E4**) (^{1}H NMR, $CDCl_3$, 400 MHz)

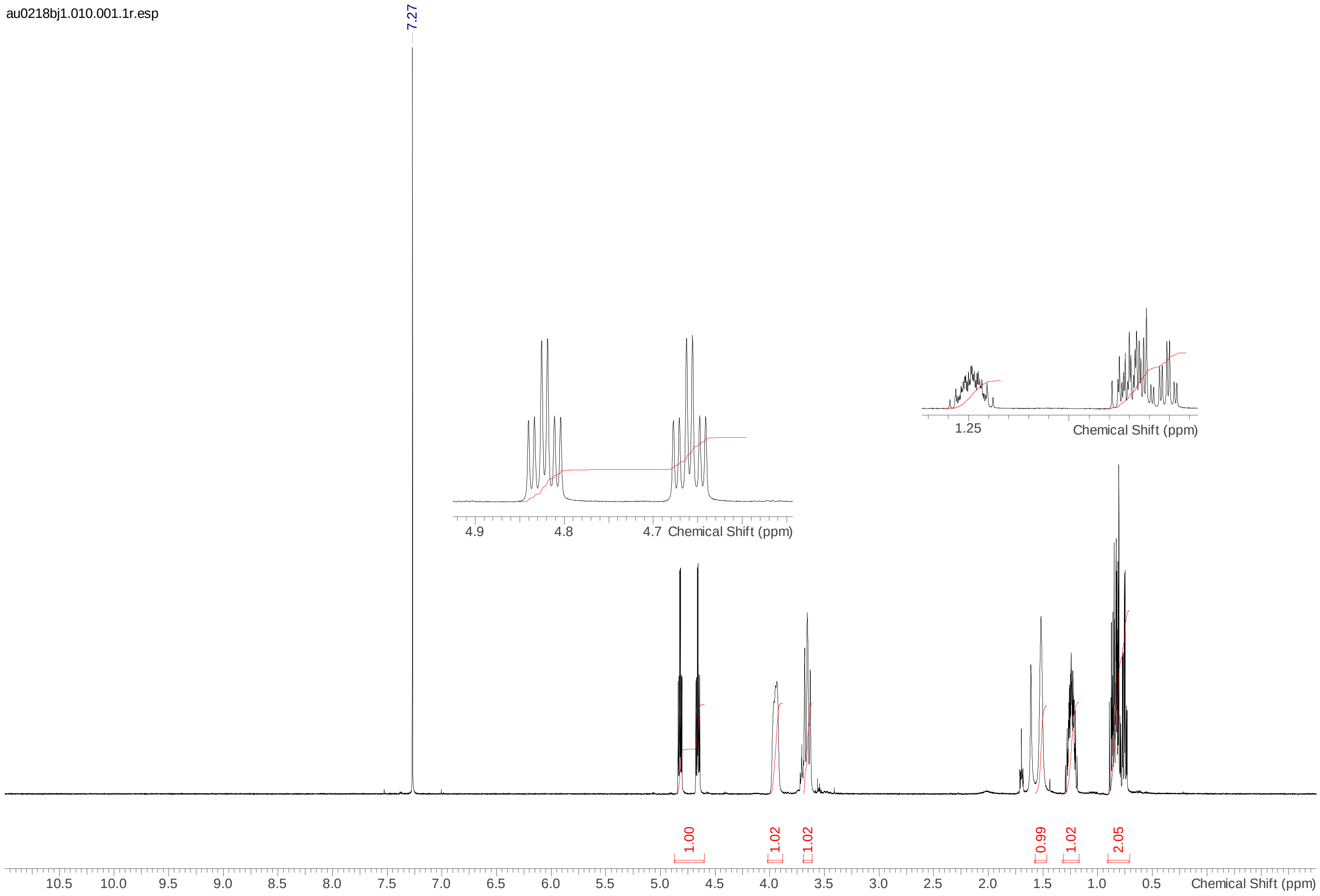

7.4.2 *rac*-(1*R*,2*S*)-(2-fluorocyclopropyl)methanol (**E4**) (^{13}C NMR, $CDCl_3$, 101 MHz)

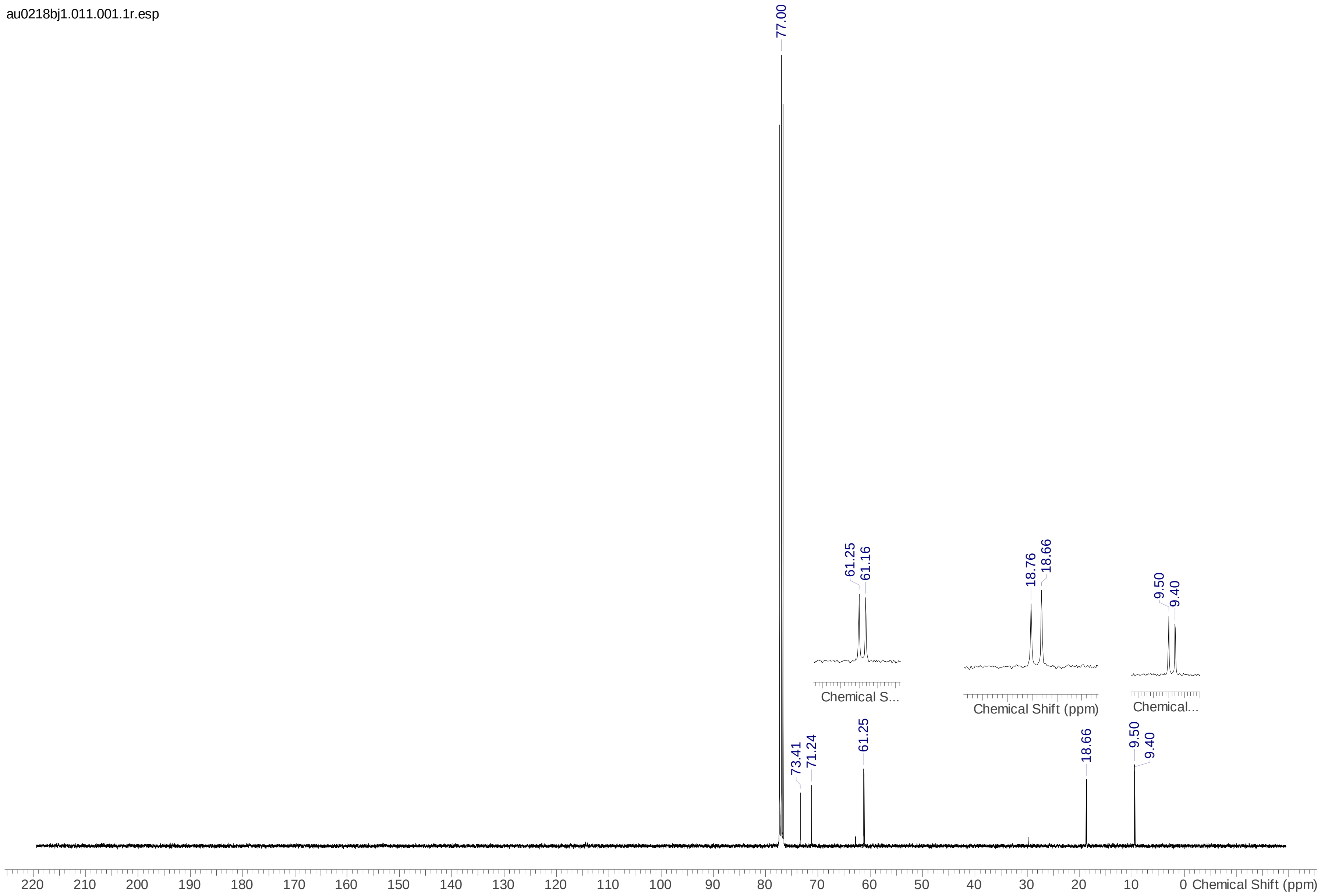

7.4.3 *rac*-(1*R*,2*S*)-(2-fluorocyclopropyl)methanol (**E4**) ^{19}F NMR, $CDCl_3$, 471 MHz

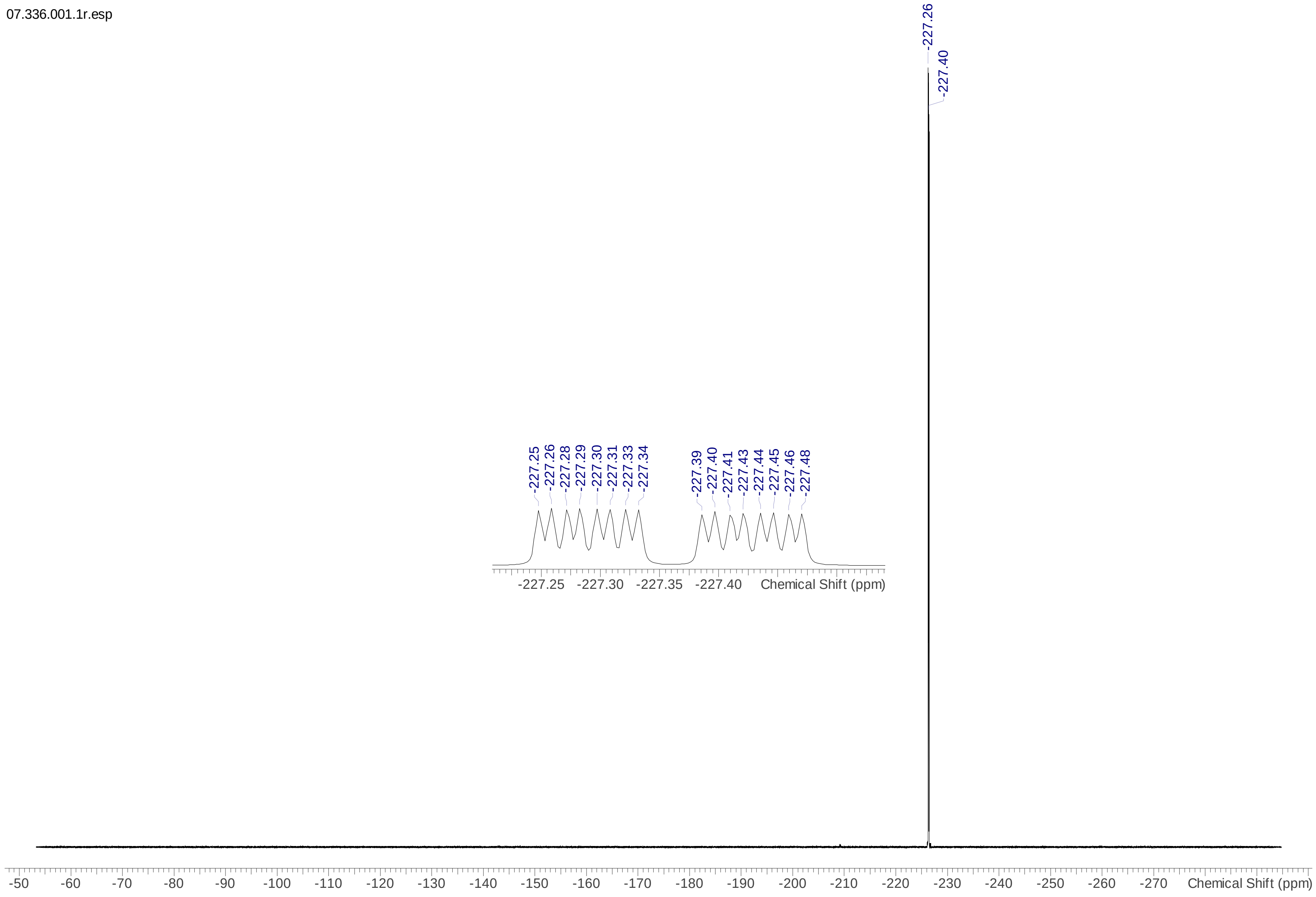

7.4.4 *rac*-(1*R*,2*S*)-(2-fluorocyclopropyl)methanol (**E4**) ^{19}F {1H} NMR, $CDCl_3$, 471 MHz

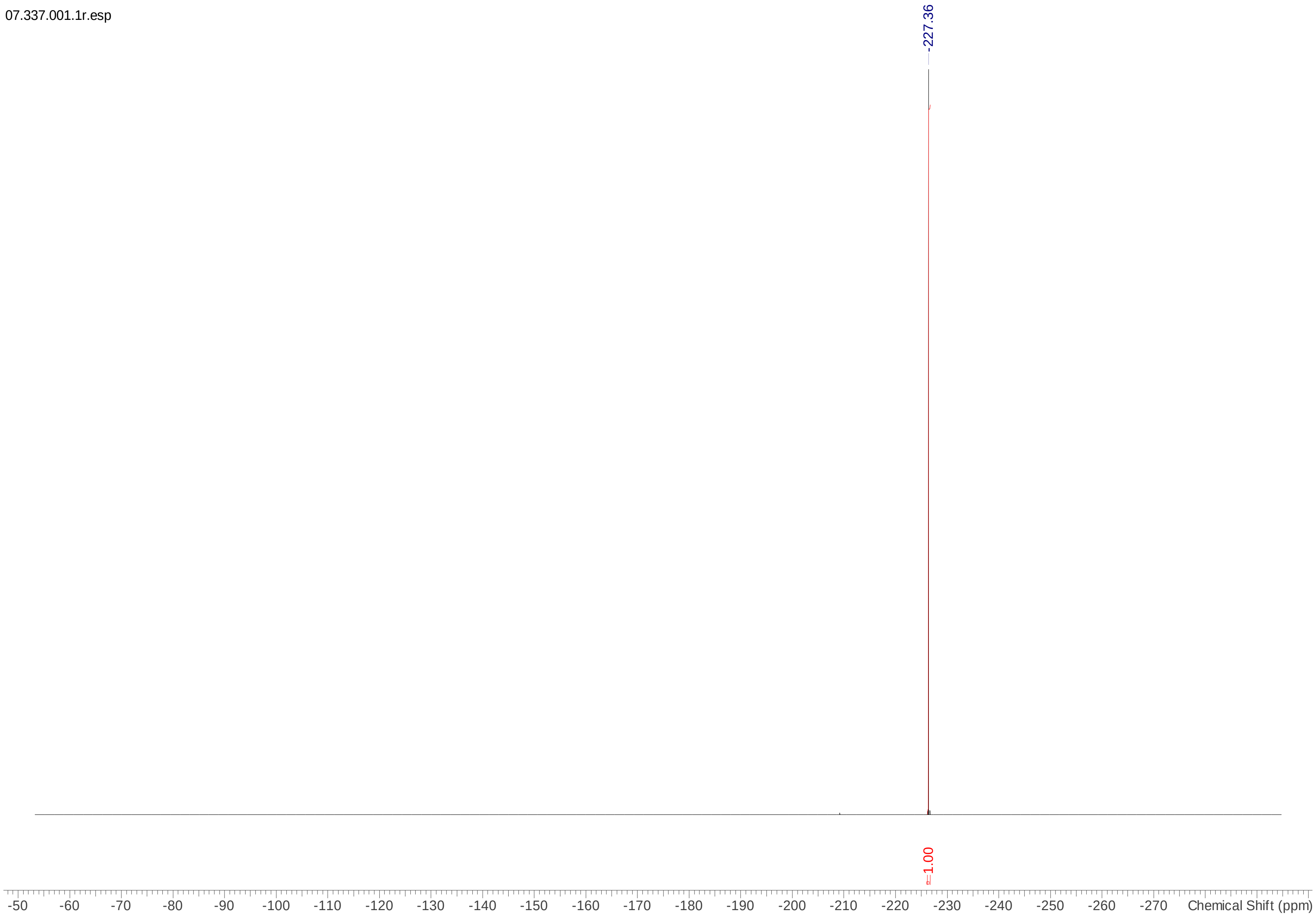

1. Linclau, B.; Wang, Z.; Compain, G.; Paumelle, V.; Fontenelle, C. Q.; Wells, N.; Weymouth-Wilson, A. Investigating the Influence of (Deoxy)fluorination on the Lipophilicity of Non-UV-Active Fluorinated Alkanols and Carbohydrates by a New log P Determination Method. *Angew Chem Int Ed Engl* **2016,** 55, 674-678.

2. Frisch, M. J.; Trucks, G. W.; Schlegel, H. B.; Scuseria, G. E.; Robb, M. A.; Cheeseman, J. R.; Scalmani, G.; Barone, V.; Petersson, G. A.; Nakatsuji, H.; Li, X.; Caricato, M.; Marenich, A. V.; Bloino, J.; Janesko, B. G.; Gomperts, R.; Mennucci, B.; Hratchian, H. P.; Ortiz, J. V.; Izmaylov, A. F.; Sonnenberg, J. L.; Williams; Ding, F.; Lipparini, F.; Egidi, F.; Goings, J.; Peng, B.; Petrone, A.; Henderson, T.; Ranasinghe, D.; Zakrzewski, V. G.; Gao, J.; Rega, N.; Zheng, G.; Liang, W.; Hada, M.; Ehara, M.; Toyota, K.; Fukuda, R.; Hasegawa, J.; Ishida, M.; Nakajima, T.; Honda, Y.; Kitao, O.; Nakai, H.; Vreven, T.; Throssell, K.; Montgomery Jr., J. A.; Peralta, J. E.; Ogliaro, F.; Bearpark, M. J.; Heyd, J. J.; Brothers, E. N.; Kudin, K. N.; Staroverov, V. N.; Keith, T. A.; Kobayashi, R.; Normand, J.; Raghavachari, K.; Rendell, A. P.; Burant, J. C.; Iyengar, S. S.; Tomasi, J.; Cossi, M.; Millam, J. M.; Klene, M.; Adamo, C.; Cammi, R.; Ochterski, J. W.; Martin, R. L.; Morokuma, K.; Farkas, O.; Foresman, J. B.; Fox, D. J. *Gaussian 16 Rev. B.01*, Wallingford, CT, 2016.

3. Yu, H. S.; He, X.; Truhlar, D. G. MN15-L: A New Local Exchange-Correlation Functional for Kohn-Sham Density Functional Theory with Broad Accuracy for Atoms, Molecules, and Solids. *J. Chem. Theory Comput.* **2016,** 12, 1280-1293.

4. Yu, H. Y. S.; He, X.; Li, S. H. L.; Truhlar, D. G. MN15: A Kohn-Sham Global-Hybrid Exchange-Correlation Density Functional with Broad Accuracy for Multi-Reference and Single-Reference Systems and Noncovalent Interactions. *Chem. Sci.* **2016,** 7, 6278-6279.

5. Marenich, A. V.; Cramer, C. J.; Truhlar, D. G. Universal Solvation Model Based on Solute Electron Density and on a Continuum Model of the Solvent Defined by the Bulk Dielectric Constant and Atomic Surface Tensions. *Journal of Physical Chemistry B* **2009,** 113, 6378-6396.

6. Ribeiro, R. F.; Marenich, A. V.; Cramer, C. J.; Truhlar, D. G. The Solvation, Partitioning, Hydrogen Bonding, and Dimerization of Nucleotide Bases: a Multifaceted Challenge for Quantum Chemistry. *Physical Chemistry Chemical Physics* **2011,** 13, 10908-10922.

7. Jeffries, B.; Wang, Z.; Felstead, H. R.; Le Questel, J. Y.; Scott, J. S.; Chiarparin, E.; Graton, J.; Linclau, B. A Systematic Investigation of Lipophilicity Modulation by Aliphatic Fluorination Motifs. *J Med Chem* **2020,** 63, 1002-1031.

8. Zampella, A.; Sorgente, M.; D'Auria, M. V. Synthetic studies on callipeltin A: stereoselective synthesis of (2R,3R,4S)-3-hydroxy-2,4,6-trimethylheptanoic acid. *Tetrahedron: Asymmetry* **2002,** 13, 681-685.

9. Mollendal, H.; Leonov, A.; de Meijere, A. Intramolecular hydrogen bonding in (1-fluorocyclopropyl)methanol as studied by microwave spectroscopy and quantum chemical calculations. *Journal of Molecular Structure* **2004,** 695, 163-169.

10. Battiste, M. A.; Tian, F.; Baker, J. M.; Bautista, O.; Villalobos, J.; Dolbier, W. R. Reactivity and regiochemical behavior in the solvolysis reactions of (2,2-difluorocyclopropyl)methyl tosylates. *Journal of Fluorine Chemistry* **2003,** 119, 39-51.
